# A whole transcriptomal linkage analysis of gene co-regulation in insecticide resistant house flies, *Musca domestica*

**DOI:** 10.1186/1471-2164-14-803

**Published:** 2013-11-19

**Authors:** Ming Li, William R Reid, Lee Zhang, Jeffery G Scott, Xiwu Gao, Michael Kristensen, Nannan Liu

**Affiliations:** 1 Department of Entomology and Plant Pathology, Auburn University, 301 Funchess Hall, Auburn, AL 36849, USA; 2 Genomics and Sequencing Laboratory, Auburn University, Auburn, AL 36849, USA; 3 Department of Entomology, Cornell University, Ithaca, NY 14853, USA; 4 Department of Entomology, China Agricultural University, Beijing, China; 5 Department of Agroecology, Aarhus University, Slagelse, Denmark

**Keywords:** Linkage analysis, Genetic crosses, House fly lines, Transcriptome, Insecticide resistance, Signaling pathways

## Abstract

**Background:**

Studies suggest that not only is insecticide resistance conferred via multiple gene up-regulation, but it is mediated through the interaction of regulatory factors. However, no regulatory factors in insecticide resistance have yet been identified, and there has been no examination of the regulatory interaction of resistance genes. Our current study generated the first reference transcriptome from the adult house fly and conducted a whole transcriptome analysis for the multiple insecticide resistant strain ALHF (wild-type) and two insecticide susceptible strains: aabys (with morphological recessive markers) and CS (wild type) to gain valuable insights into the gene interaction and complex regulation in insecticide resistance of house flies, *Musca domestica*.

**Results:**

Over 56 million reads were used to assemble the adult female *M. domestica* transcriptome reference and 14488 contigs were generated from the *de novo* transcriptome assembly. A total of 6159 (43%) of the contigs contained coding regions, among which 1316 genes were identified as being co-up-regulated in ALHF in comparison to both aabys and CS. The majority of these up-regulated genes fell within the SCOP categories of metabolism, general, intra-cellular processes, and regulation, and covered three key detailed function categories: redox detailed function category in metabolism, signal transduction and kinases/phosphatases in regulation, and proteases in intra-cellular processes. The redox group contained detoxification gene superfamilies, including cytochrome P450s, glutathione S-transferases, and esterases. The signal transduction and kinases/phosphatases groups contained gene families of rhodopsin-like GPCRs, adenylate and guanylate cyclases, protein kinases and phosphatases. The proteases group contained genes with digestive*,* catalytic, and proteinase activities. Genetic linkage analysis with house fly lines comparing different autosomal combinations from ALHF revealed that the up-regulation of gene expression in the three key SCOP detailed function categories occurred mainly through the co-regulation of factors among multiple autosomes, especially between autosomes 2 and 5, suggesting that signaling transduction cascades controlled by GPCRs, protein kinase/phosphates and proteases may be involved in the regulation of resistance P450 gene regulation.

**Conclusion:**

Taken together, our findings suggested that not only is insecticide resistance conferred via multi-resistance mechanisms or up-regulated genes, but it is mediated through the *trans* and/or *cis* co-regulations of resistance genes.

## Background

Insecticides have a major impact on agriculture, economy, and public health due to their outstanding contribution towards controlling agriculturally, medically, and economically important insect pests worldwide. Nevertheless, the development of resistance to insecticides in diverse insect pests is becoming a global problem in the insect pest control battle [1]. Resistance is thought to be a pre-adaptive phenomenon, in that prior to insecticide exposure rare individuals already exist who carry an altered genome that results in one or more possible mechanisms (factors) allowing survival from the selection pressure of insecticides [2,3] and overall, the rate of development of resistance in field populations of insects depends upon the levels of genetic variability in a population [4,5]. Efforts to characterize the genetic variation involved in insecticide resistance have thus focused on building a better fundamental understanding of the development of resistance and studying resistance mechanisms, both of which are vital for practical applications such as the design of novel strategies to prevent or minimize the spread and evolution of resistance development and the control of insect pests [6].

There is considerable evidence to suggest that the interaction of multiple insecticide resistance mechanisms or genes is responsible for the development of insecticide resistance [5,7-17]. While altering target site sensitivity to insecticides has been shown to reduce insects’ response to insecticides, transcriptional up-regulation of the detoxification machinery, increasing metabolism of insecticides into less harmful substances and facilitating insecticide excretion are all known to play a role in allowing insects to defend themselves against insecticides [14]. This detoxification machinery in insects has been mainly attributed to three enzyme systems, namely cytochrome P450s, esterases, and glutathione S-transferases (GSTs), the up-regulation of which underlies the development of insecticide resistance in many insect species. It has been suggested that new patterns of gene expression may arise via a variety of mechanisms involving changes to upstream regulators (change in *trans*) and mutations of the noncoding regulatory DNA sequences (e.g., enhancers) of a gene (change in *cis*) [18]. Indeed, many studies on the development of insecticide resistance in insects have demonstrated different patterns of gene expression between resistant and susceptible insect populations. Many studies have also found that the different patterns of gene expression in metabolic detoxification of insecticide-resistant insects are regulated by *trans* and/or *cis* factors [4,5,8,10]. Taken together, these studies suggest that not only is insecticide resistance conferred via multiple gene up-regulation, but it is mediated through the interaction of regulatory factors and resistance genes. However, no regulatory factors in insecticide resistance have yet been identified, and there has been no examination of the regulatory interaction of resistance genes. Recent advances in DNA sequencing technology have provided an opportunity for genome/whole transcriptome-wide gene discovery in organisms, including those genes suspected of involvement in insecticide resistance and the factors that may be involved in the regulation of resistance genes and mechanisms.

The house fly, *Musca domestica*, is a major domestic, medical and veterinary pest that causes more than 100 human and animal intestinal diseases, including bacterial infections such as salmonellosis, anthrax ophthalmia, shigellosis, typhoid fever, tuberculosis, cholera and infantile diarrhea; protozoan infections such as amebic dysentery; helminthic infections such as pinworms, roundworms, hookworms and tapeworms; and both viral and rickettsial infections [19-22]. Current approaches to control house flies rely primarily on source reduction and the application of insecticides, generally pyrethroids, organophosphates, neonicotinoids, as well as chitin synthesis inhibiting/disrupting larvicides. However, house flies have shown a remarkable ability to develop not only resistance to the insecticide used against them but also cross-resistance to unrelated classes of insecticides [11,20,23,24]. Because of this ability and the relatively rapid rate at which they develop resistance and cross-resistance to insecticides, their well described linkage map for five autosomes and two sex chromosomes (X and Y) [25-29], and their relatively well studied biochemistry and genetics of insecticide resistance, the house fly has proven to be a useful model for understanding and predicting resistance in other insect species.

The house fly strain ALHF has demonstrated the ability to develop resistance and/or cross-resistance to not only pyrethroids and organophosphates (OPs), but also relatively new insecticides such as fipronil and imidacloprid [11]. Genetic studies have linked pyrethroid resistance to autosomes 1, 2, 3 and 5 [5,30]. The major mechanisms governing pyrethroid resistance in this strain include increased detoxification mediated by P450 monooxygenases and decreased sensitivity of voltage-gated sodium channels (*kdr*) [15,31,32]. Previous genetic studies of ALHF have linked pyrethroid resistance primarily to autosomes 2, 3 and 5, with a minor role played by factor(s) on autosome 1 [5,30]. Furthermore, multiple P450 genes, *CYP6A5, CYP6A5v2, CYP6A36, CYP6A37, CYP4D4v2* and *CYP6A38*, that are known to be overexpressed in ALHF have been located on autosome 5 and the regulation of these P450 genes have been linked to autosomes 1 and 2 [31,32]. However, the precise nature of the interaction between the regulatory factors and resistance genes such as P450s is unclear. In an effort to better understand the genetic variation relation to resistance and gain valuable insights into the gene interaction and regulation involved in the development of permethrin resistance in the house fly, the current study generated the first adult transcriptome of the house fly *M. domestica* using Illumina RNA-Seq. Whole transcriptome comparative analyses were conducted for the resistant ALHF strain, susceptible CS and aabys strains, which enabled us to investigate the complete transcriptome of *M. domestica* and identify the genes that are most likely to be involved in pyrethroid resistance and their autosomal interactions.

## Methods

### House fly strains and lines

Three house fly strains were used in this study. ALHF, a multi-insecticide resistant strain [11] collected from a poultry farm in Alabama in 1998. This strain was further selected with permethrin in the laboratory for six generations after collection, reaching to a high level of resistance [5,30]. This strain has been maintained under biannual selection with permethrin. CS is a wild type insecticide-susceptible strain kept in laboratory breeding for more than five decades. aabys is an insecticide-susceptible strain with recessive morphological markers ali-curve (*ac*), aristapedia (*ar*), brown body (*bwb*), yellow eyes (*ye*), and snipped wings (*snp*) on autosomes 1, 2, 3, 4, and 5, respectively. Both CS and aabys were obtained from Dr. J. G. Scott (Cornell University).

**Figure 1:**
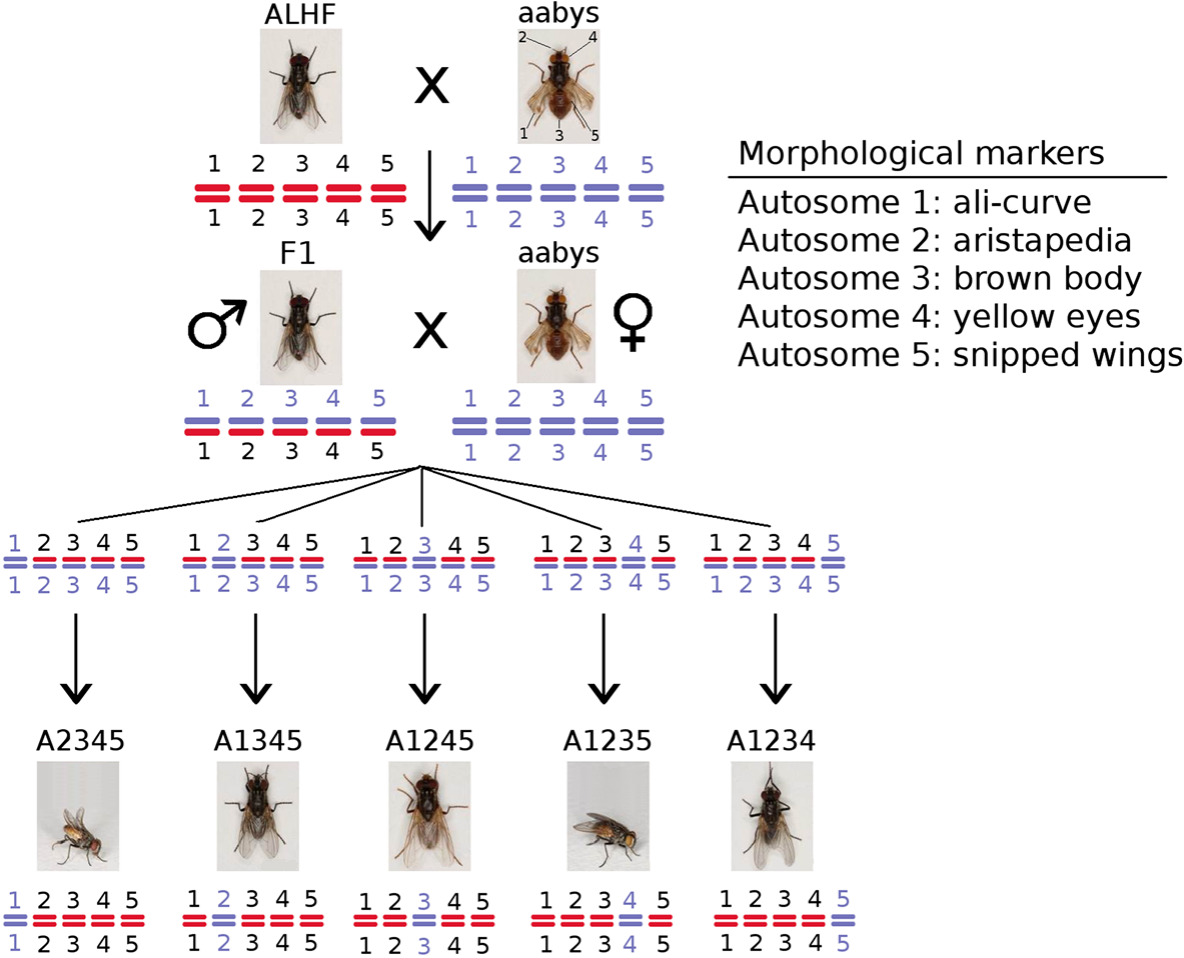
**Schematic for the generation of the *M. domestica *combination strains used in our study.** Strain ALHF is a highly insecticide-resistant strain while the aabys strain is an insecticide-susceptible strain that possesses five recessive morphological markers, with each morphological marker being uniquely present on one autosome. The images along the bottom row show the recessive morphological markers unique to each of the combination strains.

A cross of ALHF female and aabys male was performed with each of ~400 flies. The F1 males (~400 flies) were then backcrossed to aabys female (Figure 1). Five back-cross (BC_1_) lines with the following genotypes were isolated: *ac/ac*, +/*ar*, +/*bwb*, +/*ye*, +/*snp* (A2345); +/*ac*, *ar/ar*, +/*bwb*, +/*ye*, +/*snp* (A1345); +/*ac*, +/*ar*, *bwb/bwb*, +/*ye*, +/*snp* (A1245); +/*ac*, +/*ar*, +/*bwb*, *ye/ye*, +/*snp* (A1235); and +/*ac*, +/*ar*, +/*bwb*, +/*ye*, *snp/snp* (A1234). Homozygous lines (+/+, +/+, *bwb*/*bwb*, +/+, +/+ (A1245); +/+, + /+, +/+, *ye*/*ye*, +/+ (A1235); +/+, +/+, +/+, +/+, *snp*/*snp* (A1234); +/+, *ar* /*ar*, +/+, +/+,+/+ (A1345); and *ac*/*ac*, +/+, +/+, +/+, +/+ (A2345)) were accomplished by sorting for appropriate phenotypic markers and selecting with permethrin at a corresponding dose that caused ~70% mortality for each of lines for three generations. One hundred single-pair crossing (n = 100) of each of lines for the desired phenotype and genotype were then set up [5,30]. The name of each line indicates which of its autosomes bear wild-type markers from ALHF. For instance, the A2345 strain has wild-type markers on autosomes 2, 3, 4, 5 from ALHF and the mutant marker on autosome 1 from aabys. A1235 strain (flies with a recessive mutant marker on autosome 4 from aabys) showed no significant differences in resistance levels compared with ALHF based on the overlapping 95% confidence intervals for the two strains [5,30]. A2345, A1345, A1245, or A1234 house fly lines with recessive morphological markers on autosomes 1, 2, 3 or 5, respectively, from aabys had significantly decreased levels of permethrin resistance compared with ALHF, implying that factors on autosomes 1, 2, 3 and 5 play important roles in pyrethroid resistance in ALHF [30].

### RNA extraction

A total of 20 3-day old adult female house flies from each of three house fly strains (ALHF, aabys and CS) and five house fly lines (A2345, A1345, A1245, A1235, and A1234) were flash frozen on dry ice and immediately processed for RNA extraction. Total RNA was extracted using the hot acid phenol extraction method as outlined by Chomczynski and Sacchi (1987) [33]. The RNA extraction from each strain or line was performed three times with different fly samples on different days to provide biological replications for the RNA-Seq experiments (ALHF) or, later, as the replications of qRT-PCR experiments for the validation of the up-regulated genes. A total of 30 μg of RNA was subsequently treated with DNase I using the DNA-Free kit from Ambion (Austin, TX) to remove any remaining DNA and then extracted over two successive acid phenol: chloroform (1:1) steps, followed by a final chloroform extraction to remove any residual phenol. The RNA was then precipitated over ethanol at −80°C overnight, pelleted, dried, and suspended in sterile distilled water, after which a 1 μg aliquot of RNA was visually inspected for quality and for DNA contamination on a 1% agarose gel. Total RNA was sent for RNA-Seq analysis to Hudson Alpha Institute of Biotechnology (HAIB), in Huntsville, Alabama.

### RNA library preparation and RNA-Seq

RNA quality for each sample of house fly strains and lines was assessed using a Qubit fluorimeter and an Agilent 2100 Bioanalyzer at HAIB. Libraries were then prepared using Illumina Tru-Seq RNA Sample Prep Kits for mRNA-Seq using a 3' poly A selection method. Samples for the mRNA-Seq were run using the PE-50 module (HAIB) on an Illumina HiSeq 2000 instrument to generate 50 nucleotide paired end libraries. Base calling and barcode parsing were also conducted at HAIB. Data were processed to remove any reads not passing the Chastity filter and then further trimmed for the adapter using Trimmomatic [34]. Two biological replications of RNA-Seq sequencing were conducted on independent samples of the resistant ALHF strain to validate the gene expression values. All sequence traces have been submitted to the National Center for Biotechnology Information (NCBI) Short Read Archive (SRA) as accessions [NCBI:SRR521286], [NCBI:SRR521288], [NCBI:SRR521289], and [NCBI:SRR521290], and are part of Bioproject #170716 with additional information in the NCBI Gene Expression Omnibus [NCBI:GSE39327].

### Contig generation, gene annotation, gene expression determination, and data analysis

**Figure 2:**
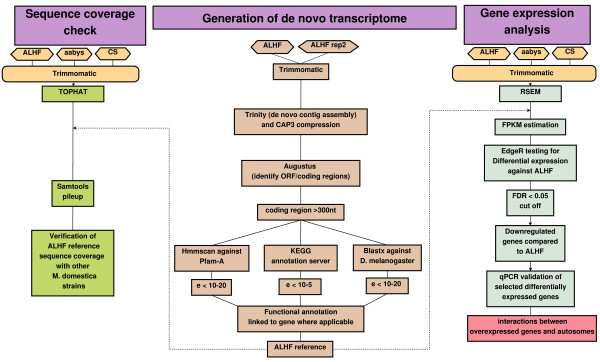
**Data analysis pipeline for the generation of the *Musca domestica *predicted gene set and differential gene expression testing.** Hexagons represent the raw data collected for this study, while terms within boxes represent either the programs, or the filtering steps used in the data analysis. The directions of the arrows indicate the data processing flow.

The bioinformatic analysis of the *M. domestica* transcriptomic data generated in this study was performed as illustrated in the flowchart in Figure 2. To generate the *M. domestica* reference transcriptome, the raw data for the two ALHF samples were pooled and then assembled *de novo* using Trinity version r2012-05-18 [35]. The standard program settings were modified to increase the Java memory to 20 GB. The contigs obtained from the Trinity build were then compressed, to reduce redundancy, using CAP3 [36] at a 95% similarity level. Compressed sequences that were <500 nt in length were discarded and the remaining contigs were further annotated to predict the gene coding region within each transcript using Augustus [37]. Within the Augustus program (Augustus version 2.5.5 [37]), the species model “FLY” based on *Drosophila melanogaster*’s genome was selected as the reference species due to the relatively close phylogenetic relationship of *D. melanogaster* and *M. domestica*. All predicted coding regions that were ≥300 nt in length were retained as the *M. domestica* ALHF strain reference transcriptome and were used for further gene expression comparisons with all of the *M. domestica* strains in our study. Within the ALHF reference transcriptome, 90% of the contigs with coding region were predicted to consist of full-length ORFs, and 10% expressed sequence tags (EST) that were missing either the 5' or the 3' ends of the predicted sequence. The functional annotations of the sequences within the ALHF reference transcriptome were then predicted using HMMScan in HMMER (v 3.0) at an e-value cut off of 10^-20^ against the Pfam-A (v26.0) hidden Markov model (HMM) database from the Wellcome Trust Sanger Institute, which is a manually-curated database of known protein domains that can be used to predict the function of an unknown protein by homology [38,39]. In addition, the ALHF reference sequences were annotated using blastx [40] against the *D. melanogaster* proteome (v. r5.46) [41] at an e-value cut off of 10^-20^, and enzyme functions were annotated by the Kyoto Encyclopedia of Genes and Genomes (KEGG) automatic annotation server (KAAS) at an e-value cut off of 10^-5^ (http://www.genome.jp/tools/kaas/; [42]). All predicted sequences have been submitted to the TSA of NCBI as accession numbers [NCBI:KA644422] through [NCBI:KA650580].

The *de novo* ALHF reference transcriptome was then used as the common reference for the estimation of the gene expression values for each of the strains. The paired end reads within each strain were mapped as paired end mate pairs using RSEM [43] to estimate the fragments per kilo base of reference gene length per million reads mapped (FPKM). The differential gene expression was then determined using EdgeR from Bioconductor at the α = 0.05 (0.05%) false discovery rate (FDR) [44-46]. To examine sequence coverage and to verify that the sequences of the ALHF reference transcriptome were present within each of the strains tested, the reads from each strain were mapped against the ALHF transcriptome reference using TopHat, with the no-novel-juncs option, to estimate the percentage of gene coverage within each strain [47] and the resulting alignment files were converted to nucleotide sequences using Samtools pileup (v0.1.13) [48] and Seqret in EMBOSS [49]. The percentage of gene coverage for each ALHF predicted gene for each house fly strain tested was then determined using faCount (http://hgwdev.cse.ucsc.edu/~kent/src/unzipped/utils/faCount/).

### Real-time quantitative RT-PCR validation of RNA-Seq data

A total of 70 genes that were differentially expressed among the different house fly strains/lines were chosen for the validation study using real-time quantitative PCR, with primers designed according to the RNA-Seq sequencing data (Additional file 1: Table S1). Total RNA was extracted from samples of 20 3-day old post-eclosion female *M. domestica* as previously described. The total RNA (0.5 μg/per sample) from each house fly sample was reverse-transcribed using SuperScript II reverse transcriptase (Stratagene) in a total volume of 20 μL. The quantity of cDNAs was measured using a spectrophotometer prior to qRT-PCR, which was performed with the SYBR Green master mix Kit and ABI 7500 Real Time PCR system (Applied Biosystems). Each qRT-PCR reaction (15 μL final volumes) contained 1× SYBR Green master mix, 1 μL of cDNA, and a gene specific primer pair at a final concentration of 0.3–0.5 μM. A 'no-template' negative control and all samples were performed in triplicate. Relative expression levels for specific genes were calculated by the 2^-ΔΔCt^ method using SDS RQ software [50]. The β-actin gene, an endogenous control, was used for internal normalization in the qRT-PCR assays [51]. Preliminary qRT-PCR experiments with the primer pair for the β-actin gene (Additional file 1: Table S1) designed according to the sequences of the β-actin gene had revealed that the β-actin gene expression remained constant in the house fly strains, so the β-actin gene was used. Each experiment was performed three times with different preparations of RNA samples. The statistical significance of the gene expressions was calculated using a Welch's t-test for all pairwise sample comparisons against the ALHF strain at a value of α = 0.05 [52].

### Genetic linkage analysis of up-regulated genes

BC_1_ lines of *ac/ac*, +/*ar*, +/*bwb*, +/*ye*, +/*snp* (A2345); +/*ac*, *ar/ar*, +/*bwb*, +/*ye*, +/*snp* (A1345); +/*ac*, +/*ar*, *bwb/bwb*, +/*ye*, +/*snp* (A1245); +/*ac*, +/*ar*, +/*bwb*, *ye/ye*, +/*snp* (A1235); and +/*ac*, +/*ar*, +/*bwb*, +/*ye*, *snp/snp* (A1234) were used to determine genetic linkage of up-regulated genes. Briefly, allele specific PCR was conducted using the cDNA from five BC_1_ house fly lines [5,31,32] for genetic mapping of the genes [10]. Two rounds of PCR were conducted. For the first PCR reaction, the allele-independent primer pairs (Additional file 1: Table S1) were designed for generating P450 (ALHF_04445.g2939 (*CYP6A36*), [31]) and ALHF_04553.g3033, carboxylesterase (ALHF_03407.g2111), adenylate cyclase (ALHF_01050.g580), protein kinase (ALHF_10712.g5974), G-protein coupled receptor (ALHF_06811.g4468), and peptidase (ALHF_07511.g4836 and ALHF_05334.g3663) cDNA fragments, respectively. The first PCR solution with cDNA template and an allele-independent primer pair was heated to 95°C for 3 min, followed by 35 cycles of 95°C for 30 s, 60°C for 30 s, and 72°C for 1 min, then 72°C for 10 min. The PCR product from this reaction was then used as the template to determine autosomal linkage. The second PCR was employed with 0.5 μL of the first round PCR reaction solution and the allele specific primer pair (Additional file 1: Table S1). The second PCR reaction was heated to 95°C for 3 min, followed by 35 cycles of 95°C for 30 s, 58°C for 30 s, and 72°C for 30 s, then 72°C for 10 min. One of each allele specific primer pair was designed based on the specific sequence of the genes from ALHF by placing a specific nucleotide polymorphism at the 3’ end of the primer to permit preferential amplification of the allele from ALHF. Each experiment was repeated three times with different mRNAs to ensure that the same autosomal linkage could be consistently repeated. The PCR products were sequenced at least once for each gene to confirm the consistency of the tested gene fragments.

## Results

### Illumina sequencing, transcriptome assembling and annotation of ALHF house flies

**Figure 3:**
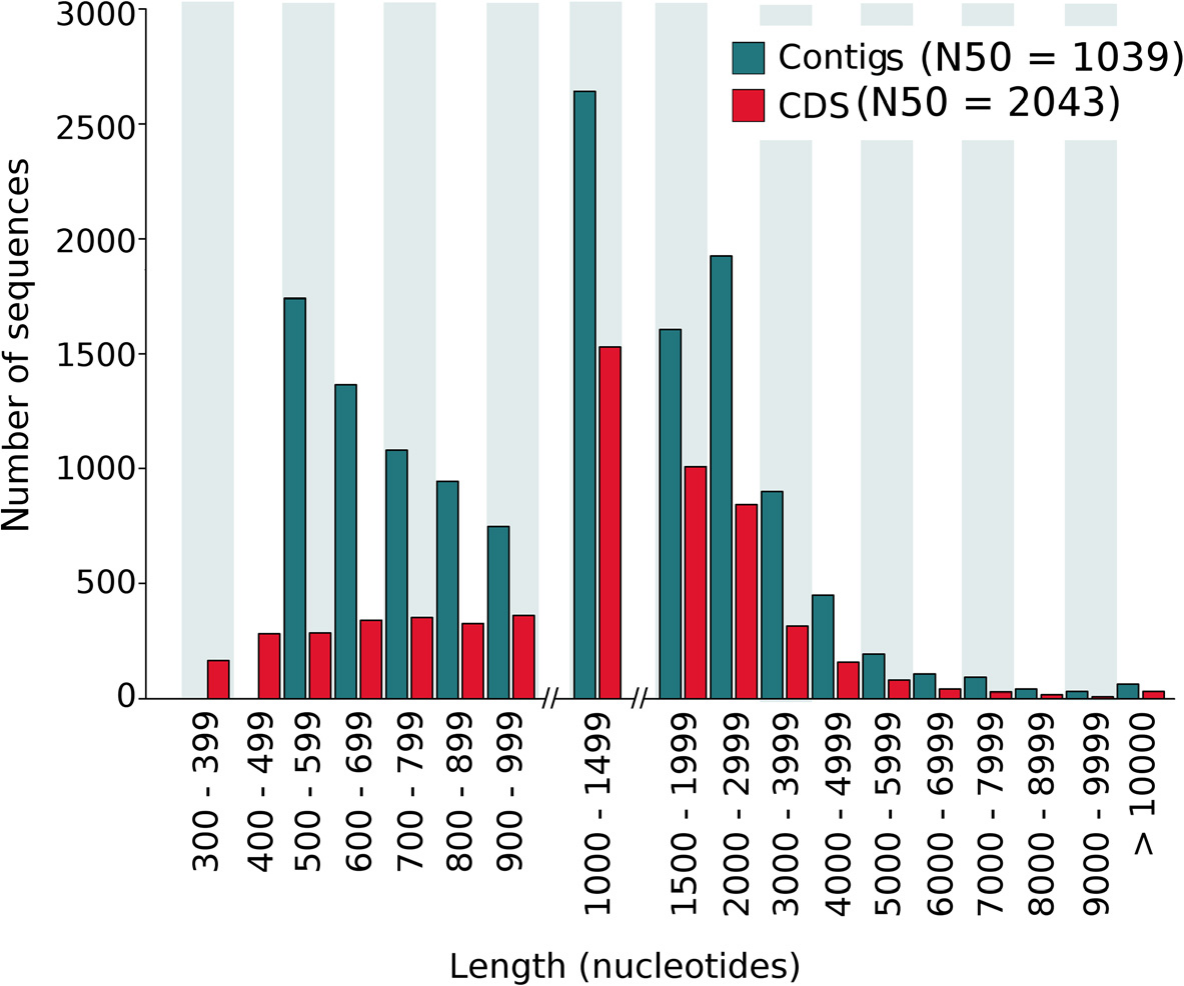
**Nucleotide length distributions for the *Musca domestica *ALHF strain raw assembled contigs and predicted coding regions (CDS).** Coding region lengths were predicted using Augustus (version 2.5.5) under the “fly” model and include both complete (5469 sequences) open reading frames (ORFs) and partial ORFs (690 sequences). A partial ORF means any sequence that is predicted to be missing either the start or the stop codon, but not both.

**Figure 4:**
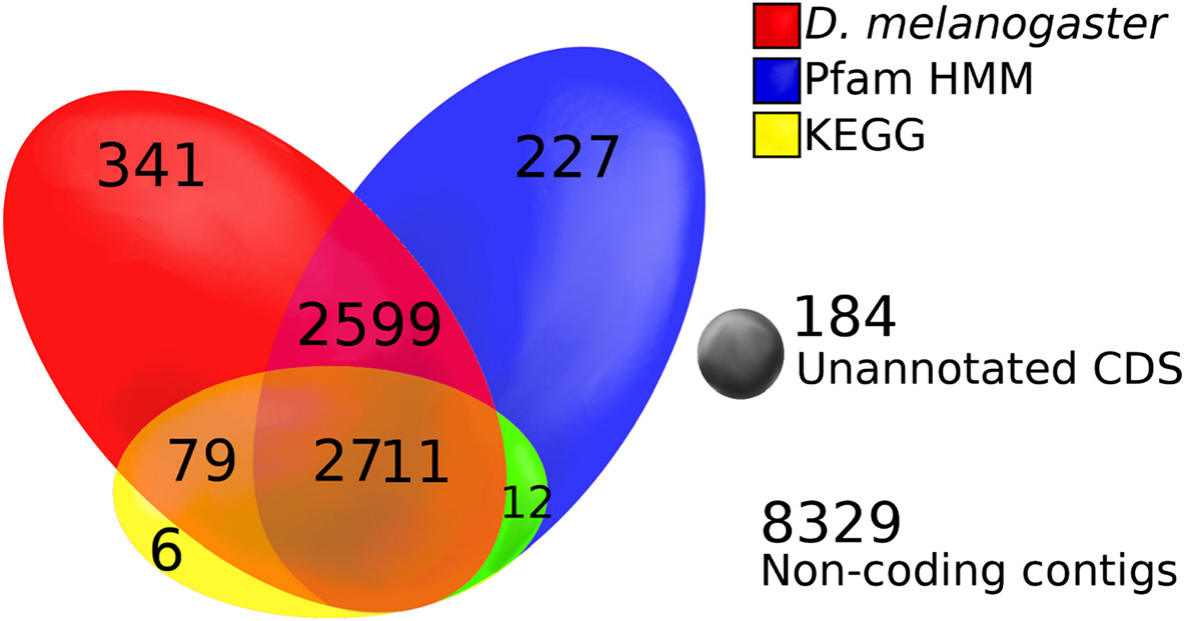
**Venn diagram for the annotation obtained for the *Musca domestica *ALHF strain predicted gene set.** Overlapping ellipses represent predicted genes from the Pfam-A (v26.0), the *Drosophila melanogaster* proteome (v. r5.46), and the Kyoto Encyclopedia of Genes and Genomes (KEGG) automatic annotation server (KAAS) that could be annotated in two or more of the databases used to predict gene function. An e-value threshold for homology detection was fixed at 10^-20^ for the Pfam and blastx analyses and at 10^-5^ for KEGG. The circles excluded from the overlapping ellipses represent sequences which contained the coding region, but had no homologs in any of the three databases used for gene prediction.

The number of paired end reads for each of the house fly strains ranged from 25–37 million, with an average of 7% of the reads discarded due to low quality (Table 1). The two RNA-Seq biological replicates from the ALHF strain ([NCBI:SRR521288] and [NCBI:SRR521289]) had sequence depths of 25 and 28 million reads, respectively. After the sequence cleaning steps, the two RNA-Seq sequences from ALHF were pooled, resulting in 53 million reads that could then be used to assemble the ALHF transcriptome reference. After the Trinity *de novo* transcriptome assembling [35] and CAP3 processing steps [36], 14488 contigs were generated from the adult female ALHF *M. domestica de novo* transcriptome assembly (Table 2). The majority of the contigs ranged from 500 to 1500 nt in length (Figure 3). The N50, the central tendency of the contig length, was 1039 nt, indicating that half of the total number of nucleotides used for the entire transcriptome assembly were contained within contigs with ≥1039 nt in length [53]. Within the 14488 contigs, a total of 6159 (43%) of them contained coding regions with >500 nt length, 5469 of which had complete putative open reading frames (ORFs). The nucleotide sequence information for house flies has been submitted to the NCBI Transcriptome Shotgun Assembly (TSA) *(*http://www.ncbi.nlm.nih.gov/genbank/tsa/). The complete annotation spreadsheet for the *M. domestica* predicted gene set is provided in Additional file 2: Table S2, including the predicted gene name, nucleotide length, the TSA accession number from NCBI, the *D. melanogaster* blastx homology, the Superfamily general and detailed function annotations, the Pfam-A HMM homology, the KEGG homology, and the putative GO terms based on the *M. domestica* predicted gene homology to the *D. melanogaster* proteome. The predicted gene set had an N50 of 2043 nt and the majority of the genes ranged in length from 1000 to 3000 nt (Figure 3). Among the 6159 annotated sequences, 5975 had significant hits to three different databases. A total of 5549 sequences had significant hits for the Pfam-A HMM library (v26.0), representing 2147 gene families [38,39], while 5730 sequences had significant hits for *D. melanogaster* (v. r5.46) [41] (Figure 4). Since ~93% of the sequences had significant (e-value < 10^-20^) matches to *D. melanogaster*, we used the bioinformatic information available for the *D. melanogaster* Structural Classification of Proteins (SCOP) functional annotation (http://supfam.cs.bris.ac.uk/SUPERFAMILY/) as a reference for the *M. domestica* transcriptome. To provide a general overview of the gene discovery in the adult *M. domestica* transcriptome, the predicted genes in *M. domestica* were thus classified according to their sequence homology to the functional categories of *D. melanogaster* in the SCOP database (Table 3) (http://supfam.cs.bris.ac.uk/SUPERFAMILY/). The SCOP functional category annotation sorts the genes from *D. melanogaster* into eight general function categories, which are then divided into detailed functional categories. A total of 1963 genes, which was approximately one-third of the 6159 genes, were placed into the non-annotated category of the SCOP general function category, and represented the largest SCOP general function category. The second most abundant general function category was the metabolism category, encompassing 17% of all predicted genes, followed by general function (15%), regulation (15%), intracellular processes (13%), information (4%), extracellular processes (2%), and “other” (1%). The Kyoto Encyclopedia of Genes and Genomes (KEGG) was also used for gene annotation in order to identify those genes with putative enzymatic function. Taken together, the use of multiple databases for the functional annotation of the predicted genes in the *M. domestica* adult transcriptome allowed us to categorize the *M. domestica* genes into higher (general and detailed function) and lower (family and gene function) levels of annotation.

**Table 1:** **Total Illumina reads for each of the *Musca domestica *** **strains/lines tested**

**Sample**	SRA^* ^**accession**	Total PE^** ^**reads passing chastity filter**	**Discarded**^**‡**^	**Reads used**
aabys	SRR521286	40284931	2794419	37490512
CS	SRR521290	34589399	2561540	32027859
ALHF	SRR521289	30329576	2208815	28120761
ALHF- replicate	SRR521288	26151304	1406309	24744995

^*^Sequence Read Archive, National Center for Biotechnology Information (http://www.ncbi.nlm.nih.gov/sra/).

^**^51 nt Paired-end reads with an average insert size of 200 nt.

^‡^Discarded reads were removed using Trimmomatic based on a sliding window quality cut off of (4:15) and a minimum length of 36 nt after adapter removal. In addition all mate pairs were excluded from analysis if one of the mate pairs was rejected by Trimmomatic.

**Table 2:** **Results of the homology testing for the transcriptome assembly for the adult female *Musca domestica *** **ALHF strain**

**Assembly results**	**Total number**	**Unique annotation**
Contigs generated		
Non annotated contigs^†^	8229	-
Predicted ORFs	5469	-
5' Partial ESTs	519	-
3' Partial ESTs	171	
Contigs annotated		
No annotation^*^	184	-
*D. melanogaster*^‡^	5730	4265
Pfam family^**^	5549	2148
KEGG^††^	2795	1967

^†^"Non annotated contig" indicates that no ORF was identified in the contig.

^*^"No annotation" indicates the presence of an ORF, a start, and a stop codon, but no homology to the Pfam family HMM search, *Drosophila melanogaster*, or KEGG.

^**^Pfam HMM A (v26.0) Nov 2011. http:// pfam.sanger.ac.uk/*.*

^‡^*version r5.46 20 Feb, 2009.*http://www.flybase.org/*.*

^††^*Release 62.0 April 1, 2012.*http://www.genome.jp/kegg/*.*

**Table 3:** **Higher level SCOP annotation for the predicted genes from the adult *Musca domestica *** **transcriptome based on sequence homology to *Drosophila melanogaster***^**§**^

**General function**	**Detailed function**	**Superfamilies**	**Predicted genes**
Metabolism	Amino acids metabolism /transport	6	17
	Carbohydrate metabolism /transport	11	94
	Coenzyme metabolism /transport	13	45
	Electron transfer	7	28
	Energy	23	45
	Lipid metabolism /transport	4	18
	Nitrogen metabolism /transport	1	2
	Nucleotide metabolism /transport	14	74
	Other enzymes	51	344
	Photosynthesis	1	2
	Polysaccharide metabolism /transport	2	19
	Redox	28	180
	Secondary metabolism	6	68
	Transferases	14	143
Regulation	DNA-binding	32	218
	Kinases/phosphatases	7	245
	Other regulatory function	5	12
	Receptor activity	2	5
	RNA binding, metabolism /transport	12	113
	Signal transduction	30	301
Information	Chromatin structure	4	4
	DNA replication/repair	15	141
	RNA processing	6	25
	Transcription	7	17
	Translation	44	77
Extra-cellular processes	Blood clotting	1	13
	Cell adhesion	18	101
	Immune response	4	22
	Toxins/defense	2	5
Intra-cellular processes	Cell cycle, Apoptosis	10	26
	Cell motility	11	38
	Ion metabolism /transport	13	173
	Phospholipid metabolism /transport	4	36
	Proteases	21	304
	Protein modification	15	105
	Transport	22	118
General	General	12	348
	Ion binding	1	6
	Ligand binding	1	4
	Protein interaction	19	178
	Small molecule binding	11	403
Other	Unknown function	26	75
	Viral proteins	1	4
NONA	not annotated	1	1963
	TOTAL	537	6159

^§^SCOP Superfamily annotation for *D. melanogaster* (v. 66_539) http://supfam.cs.bris.ac.uk/SUPERFAMILY/cgi-bin/gen_list.cgi?genome=dd.

### House fly transcriptome reference and gene expression profiles

**Figure 5:**
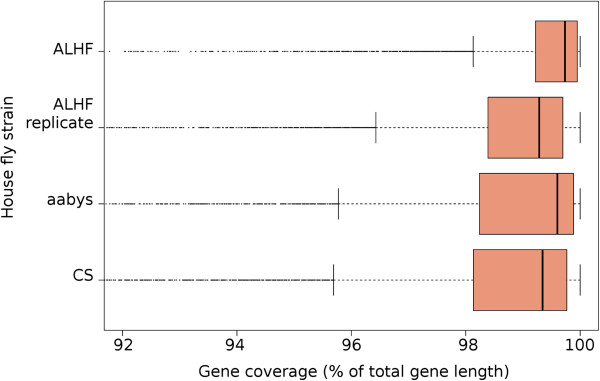
**Box and whisker plots representing the interquartile ranges (IQR) for the nucleotide coverage of each of the ALHF strain predicted genes in each of the *Musca domestica *strains tested.** The dependent axis has been broken to make the IQR and median values discernible. The solid line within each of the boxes represents the median value for the gene coverage for each house fly strain.

The ALHF transcriptome was used as the reference for the comparison of gene expression between the resistant ALHF and the susceptible aabys and CS strains of *M. domestica*. To verify that the predicted genes within the ALHF transcriptome also provided good coverage for the other *M. domestica* strains tested, we independently mapped the raw Illumina reads from each of the *M. domestica* strains to the ALHF reference transcriptome using Tophat and then determined the percentage of gene coverage for each of the genes within each *M. domestica* strain. The results showed that the median nucleotide coverage for the 6159 genes within the ALHF transcriptome was >99% for all of the *M. domestica* strains tested (Figure 5), demonstrating that the transcriptome from ALHF was indeed a suitable reference for the determination of gene expression levels for all the strains.

**Figure 6:**
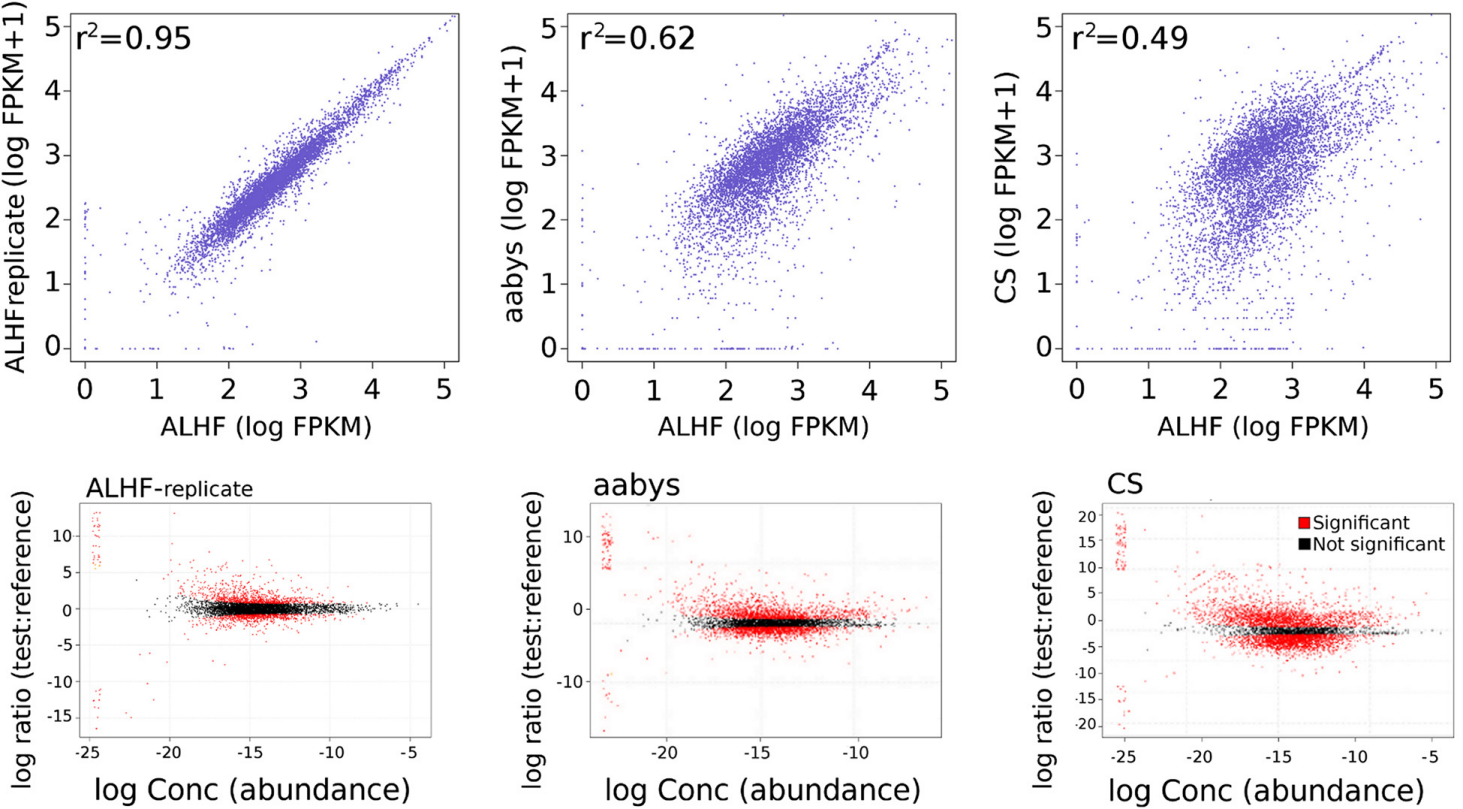
**Correlation of the gene expression levels (FPKM) for all of the *Musca domestica *strains tested versus the ALHF pyrethroid-resistant strain (upper panels).** Scatterplots represent the differential gene expression compared to the ALHF strain (lower panels). In the upper panels, the points closest to the 1:1 line represent genes with the same gene expression value as the ALHF strain and the tested *M. domestica* strain. In the lower panels, each point represents a gene, with red points below the central axis indicating the genes that were down-regulated in the tested *M. domestica* strain compared to the ALHF strain, thus the red points below the horizontal axis on the lower panels represent the genes that were up-regulated in the pyrethroid-resistant ALHF strain and putatively linked to insecticide resistance.

The program RSEM [43] was therefore used to estimate the gene expression values (FPKM) for all the *M. domestica* strains using the ALHF transcriptome as the reference. The gene expression values from the susceptible aabys and CS strains were then compared with the gene expression values of the ALHF strain to determine differential gene expression using EdgeR [44] with a 0.05 false discovery rate (α = 0.05, [45,46]). In addition to testing the two susceptible strains of *M. domestica* for differential gene expression, we further tested the gene expression values of the ALHF strain against an additional biologically-independent sample of the ALHF strain to ensure that the gene expression values were reproducible. When the gene expression values of the two ALHF samples were compared, the results showed a strong 1:1 correlation (r^2^ = 0.95); the correlation coefficients for the aabys and CS strains were 0.62 and 0.49, respectively (Figure 6). In addition, <10% (606) of the genes tested as differentially expressed between the two ALHF replicates, while the aabys and CS strains had 3428 and 4792 genes that were differentially expressed, respectively (Figure 6). Since gene over-expression has been linked to insecticide resistance [4,54-57], the genes identified as differentially up-regulated in the ALHF strain when compared to both the insecticide-susceptible aabys and CS strains represent the genes putatively involved in pyrethroid resistance.

Overall, a total of 1316 genes were identified as being co-up-regulated in ALHF in comparison to both aabys and CS (Additional file 3: Table S3). While one-third of these genes (452 genes) were distributed within the SCOP general function category of “no annotation” (Additional file 3: Table S3), the majority (777 genes) fell within the SCOP categories of metabolism, general, intra-cellular processes, and regulation, containing 275, 178, 174, and 150 genes, respectively (Additional file 3: Table S3). The metabolism SCOP general function category had the greatest number of detailed function groups (13 groups), among which the redox detailed function group contained the second largest number of genes and detoxification enzymes such as cytochrome P450s, glutathione-S-transferases (GSTs) and esterases (Additional file 3: Table S3). Interestingly, within the regulation category the detailed function groups with the greatest number of genes were involved with signal transduction, kinases/phosphatases, and DNA binding, while in the intra-cellular processes category the proteases were the most abundant detailed function group. A total of 1440 genes were identified as down-regulated in the ALHF strain when compared to the susceptible aabys and CS strains (Additional file 3: Table S3), among which one-third (458) of the genes had no SCOP annotation. The rest of the down-regulated genes were distributed within the SCOP general function category of regulation (254 genes), general (242 genes), metabolism (194 genes), intra-cellular processes (152 genes), information (105 genes), other (22 genes), and extra-cellular processes (13 genes).

### Validation of the expression of up-regulated genes in house fly strains/lines

**Figure 7:**
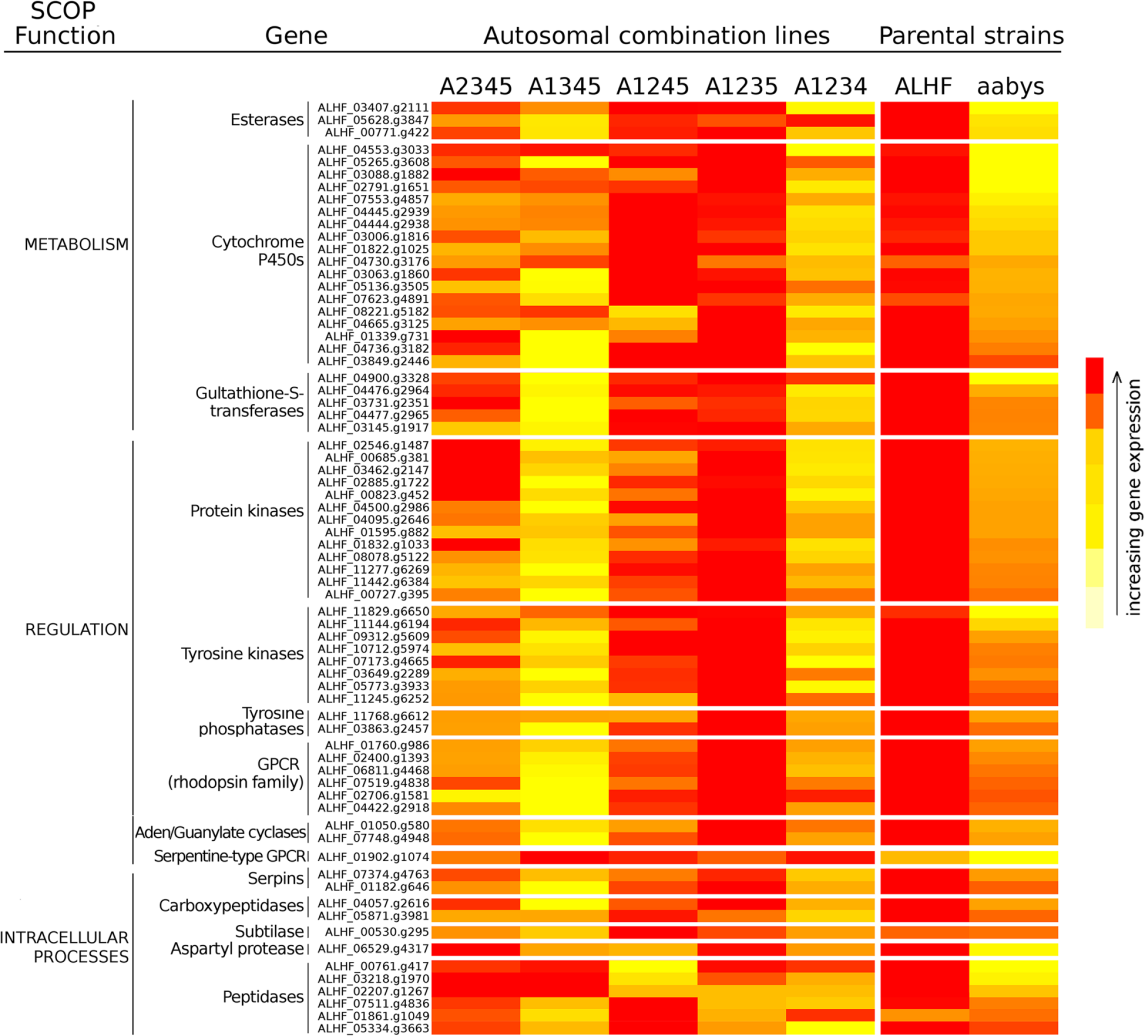
**Heat map of the gene expression values (within gene) relative to aabys for each of the genes tested by qPCR to validate the gene expression levels within the different *Musca domestica *lines and the parental ALHF and aabys strains.** Colors scaled from yellow to red indicate low to higher gene expression, respectively, relative to aabys.

A total of 70 genes were selected from the predominant groups of up-regulated genes identified by RNA-Seq, including multiple cytochrome P450s, GSTs, and esterases in metabolism; kinases/phosphatases, 7 transmembrane receptors (rhodopsin-like G-protein coupled receptor (GPCR) family), adenylate and guanylate cyclases in regulation; and serpins and carboxypeptidases in intracellular processes for further validation by qRT-PCR. We examined the expression of these 70 genes in resistant ALHF, susceptible aabys and five house fly homozygous lines A2345, A1345, A1245, A1235, and A1234, the lines represent the ALHF strain where autosomes 1, 2, 3, 4, and 5, respectively, have been replaced by the autosome from aabys. Overall, the biological replication of qRT-PCR results showed that the expression of the majority of genes (81%) was consistent with the RNA-Seq data, being highly expressed in resistant ALHF compared with the susceptible aabys (Table 4). We also examined the expression levels of the up-regulated genes in ALHF for the five house fly lines to determine the effects of factors from the different autosomes of ALHF on the up-regulation of the genes. Clear changes in the gene expression levels were identified when each autosome in ALHF was replaced by the corresponding aabys autosome, i.e., lines A2345, A1345, A1245, A1235, and A1234 (Figure 7, Table 4). In general, no significant change in the level of expression was observed for most of the selected genes, when autosome 4 of ALHF (i.e., line A1235, Figure 7, Table 4) was replaced with that from aabys except for four protease genes. Previous research by Tian et al., [30] identified that when autosome 4 in ALHF was replaced by the one from aabys, there was no significant decrease in resistance. Liu and Scott (1995) also demonstrated that replacement of autosome 4 in the resistant LPR house flies with the one from aabys, the resistance level was not changed [4]. Liu and Yue (2001) reported the similar results in house flies [5]. Taken together, these results strongly revealed that factors/genes on autosome 4 do not have a major role in the up-regulation of genes in ALHF, although further investigation of the up-regulated protease genes in resistance is needed. The majority of the selected up-regulated genes exhibited no change in expression when autosome 1 or 3 in ALHF was replaced by the corresponding autosome from aabys (i.e., lines A2345 or A1245, respectively). However, significant changes in the gene expression for most of the selected up-regulated genes (>90%) were observed when autosome 2 or 5 in ALHF was replaced by the corresponding autosome from aabys (i.e., lines A1345 or A1234 respectively). These results suggest the importance of factors on autosome 2 and/or 5 for the expression of up-regulated genes in ALHF and/or that several of the up-regulated loci reside on the replaced chromosomes.

**Table 4:** **Gene expression values and the predicted autosomal interactions for the selected genes linked to pyrethroid resistance in *Musca domestica *** **as assayed by qPCR**

**SCOP**^**† **^**functional annotation**		**Predicted gene function**	**Accession number**	**Relative gene expression ± SE**					
**General**	**Detailed**	**Pfam annotation**^**§**^	**Gene**	**ALHF**	**A2345**	**A1345**	**A1245**	**A1235**	**A1234**
Metabolism	Other	Carboxylesterase	ALHF_03407.g2111	3.1 ± 0.2	2.4 ± 0.1	1.8 ± 0.12*	3.0 ± 0.1	2.9 ± 0.1	1.1 ± 0.11*
			ALHF_05628.g3847	6.6 ± 1.3	2.1 ± 0.3*	1.0 ± 0.1*	4.0 ± 0.4	3.2 ± 0.1	4.4 ± 0.8
			ALHF_00771.g422	26 ± 5.5	14 ± 1.2	0.24 ± 0.07*	18 ± 1.5	26 ± 2	3.1 ± 0.3*
	Redox	Cytochrome P450	ALHF_04553.g3033	2.7 ± 0.09	2.5 ± 0.1	2.7 ± 0.2	2.5 ± 0.08	3.0 ± 0.2	0.8 ± 0.03*
			ALHF_05265.g3608	510 ± 28	300 ± 5.0	15 ± 2.6*	530 ± 16	500 ± 18	300 ± 4.3
			ALHF_03088.g1882	310 ± 9.3	310 ± 10	190 ± 9.0	150 ± 4.4	310 ± 18	120 ± 6.4*
			ALHF_02791.g1651	12 ± 0.2	7.4 ± 0.7	7.9 ± 0.04	8.8 ± 0.2	13 ± 0.9	2.4 ± 0.09*
			ALHF_07553.g4857	6.2 ± 0.6	2.6 ± 0.4*	3.1 ± 0.6*	9.5 ± 2.3	6.2 ± 0.5	2.6 ± 0.3*
			ALHF_04445.g2939	3.0 ± 0.1	1.6 ± 0.2*	1.8 ± 0.4*	4.0 ± 0.4	3.0 ± 0.04	1.0 ± 0.2*
			ALHF_04444.g2938	3.6 ± 0.06	1.9 ± 0.1	2.1 ± 0.09	5.6 ± 2.1	3.6 ± 0.1	1.0 ± 0.2*
			ALHF_03006.g1816	2.3 ± 0.09	1.9 ± 0.3	1.1 ± 0.08*	3.8 ± 0.4	2.1 ± 0.06	0.9 ± 0.1*
			ALHF_01822.g1025	4.3 ± 1.0	1.3 ± 0.2*	1.9 ± 0.4*	5.6 ± 1.2	4.2 ± 0.2	0.6 ± 0.1*
			ALHF_04730.g3176	2.1 ± 0.02	1.2 ± 0.2	2.6 ± 0.3	7.1 ± 2.7	1.8 ± 0.09	0. 7 ± 0.09*
			ALHF_03063.g1860^††^	-	-	-	-	-	-
			ALHF_05136.g3505	2.4 ± 0.3	0.9 ± 0.1*	0.4 ±0.2*	3.2 ± 0.4	2.5 ± 0.2	1.5 ± 0.1
			ALHF_07623.g4891	1.9 ± 0.2	1.8 ± 0.2	0.4 ± 0.2*	4.6 ± 1.2	2.2 ± 0.1	1.0 ± 0.1 *
			ALHF_08221.g5182	2.6 ± 0.2	1.6 ± 0.04	1.8 ± 0.3	0.6 ±0.04	2.9 ± 0.04	0.6 ± 0.05*
			ALHF_04665.g3125	2.9 ± 0.08	1.0 ± 0.05*	1.1 ± 0.06*	0.8 ±0.03*	2.8 ± 0.09	1.0 ± 0.03*
			ALHF_01339.g731	2.0 ± 0.08	2.3 ± 0.07	0.2 ± 0.03*	1.1 ± 0.03	2.1 ± 0.07	0.8 ± 0.04*
			ALHF_04736.g3182^††^	-	-	-	-	-	-
			ALHF_03849.g2446^††^	-	-	-	-	-	-
		Glutathione-S-transferase	ALHF_04900.g3328	2.8 ± 0.4	2.1 ± 0.2	0.9 ± 0.08*	2.3 ± 0.2	2.8 ± 0.2	2.2 ± 0.2
			ALHF_04476.g2964	2.4 ± 0.2	1.7 ± 0.2	0.6 ±0.07*	1.9 ± 0.2	1.8 ± 0.08	0.7 ± 0.02*
			ALHF_03731.g2351	2.4 ± 0.3	1.8 ± 0.3	0.4 ± 0.03*	1.2 ± 0.08	1.5 ± 0.07	0.6 ± 0.08*
			ALHF_04477.g2965	1.5 ± 0.02	1.1 ± 0.11	0.6 ± 0.07*	1.7 ± 0.1	1.3 ± 0.06	0.8 ± 0.1*
			ALHF_03145.g1917	1.9 ± 0.2	0.7 ± 0.06*	0.5 ± 0.01*	1.6 ± 0.2	1.8 ± 0.06	0.9 ± 0.02*
Regulation	Kinase / phosphatase	Protein kinase domain	ALHF_02546.g1487	2.9 ± 0.09	3.8 ± 0.75	0.4 ± 0.04*	2.2 ± 0.3	2.6 ± 0.2	0.5 ± 0.08*
			ALHF_00685.g381	3.6 ± 0.4	3.2 ± 0.7	0.8 ±0.2*	1.1 ± 0.09*	3.3 ± 0.3	0.5 ± 0.04*
			ALHF_03462.g2147	3.7 ± 0.7	4.1 ± 0.4	0.5 ± 0.01*	1.5 ± 0.2*	3.5 ± 0.2	0.2 ± 0.2*
			ALHF_02885.g1722	1.8 ± 0.2	1.8 ± 0.2	0.6 ±0.2*	1.5 ± 0.8	1.6 ± 0.1	0.8 ±0.1*
			ALHF_00823.g452	2.0 ± 0.2	1.8 ± 0.2	0.8 ± 0.2*	1.2 ± 0.09	1.8 ± 0.2	0.7 ± 0.1 *
			ALHF_04500.g2986	1.7 ± 0.09	1.1 ± 0.04	0.7 ± 0.2*	1.5 ± 0.1	1.7 ± 0.04	0.9 ± 0.06*
			ALHF_04095.g2646	1.7 ± 0.1	1.1 ± 0.06	0.9 ±0.2	1.0 ± 0.08	1.5 ± 0.03	1.0 ± 0.2
			ALHF_01595.g882	2.3 ± 0.2	0.8 ± 0.06*	0.8 ± 0.1*	1.4 ± 0.2	2.5 ± 0.2	1.0 ± 0.1*
			ALHF_01832.g1033	2.3 ± 0.2	2.8 ± 0.6	0.4 ± 0.09*	1.0 ± 0.06*	1.9 ± 0.1	0.4 ± 0.06*
			ALHF_08078.g5122	2.6 ± 0.7	1.0 ± 0.04	0.5 ± 0.03*	1.7 ± 0.1	2.4 ± 0.2	0.6 ± 0.06*
			ALHF_11277.g6269	2.3 ± 0.1	0.7 ± 0.05*	0.2 ± 0.02*	1.8 ± 0.1	2.1 ± 0.2	0.8 ± 0.2*
			ALHF_11442.g6384^††^	-	-	-	-	-	-
			ALHF_00727.g395^††^	-	-	-	-	-	-
		Protein tyrosine kinase	ALHF_11829.g6650	1.7 ± 0.08	1.3 ± 0.09	1.5 ± 0.17	2.2 ± 0.4	1.9 ± 0.1	1.3 ± 0.1
			ALHF_11144.g6194	4.0 ± 0.8	3.2 ± 0.2	1.4 ± 0.2*	2.5 ± 0.7	5.1 ± 0.2	0.8 ± 0.04*
			ALHF_09312.g5609	1.9 ± 0.1	1.3 ± 0.1	0.7 ± 0.1*	1.7 ± 0.2	1.7 ± 0.06	0.7 ± 0.1*
			ALHF_10712.g5974	1.8 ± 0.1	0.7 ± 0.04*	0.6 ± 0.2*	1.7 ± 0.3	1.8 ± 0.08	0.7 ± 0.1*
			ALHF_07173.g4665	1.5 ± 0.1	1.3 ± 0.1	0.8 ± 0.05*	1.2 ± 0.2	1.6 ± 0.07	0.5 ± 0.1*
			ALHF_03649.g2289	2.1 ± 0.1	0.9 ± 0.07*	0.4 ± 0.2*	1.5 ± 0.2	2.1 ± 0.2	1.1 ± 0.06*
			ALHF_05773.g3933^††^	-	-	-	-	-	-
			ALHF_11245.g6252	1.7 ± 0.07	0.6 ± 0.03*	0.2 ± 0.06*	0.5 ± 0.2*	1.9 ± 0.09	0.9 ± 0.09*
		Protein-tyrosine phosphatase	ALHF_11768.g6612	39 ± 4.9	1.6 ± 0.07*	0.6 ± 0.05*	0.4 ± 0.1*	36 ± 3.8	0.5 ± 0.03*
			ALHF_03863.g2457	1.5 ± 0.06	0.9 ± 0.09	0.5 ± 0.04*	1.2 ± 0.2	1.7 ± 0.06	0.9 ± 0.08*
	Signal transduction	GPCR (rhodopsin family)	ALHF_01760.g986	1.6 ± 0.09	1.0 ± 0.08	0.9 ± 0.1*	1.1 ± 0.03	1.7 ± 0.1	1.0 ± 0.1
			ALHF_02400.g1393	1.7 ± 0.07	0.9 ± 0.07	0.7 ± 0.05*	1.2 ± 0.2	1.6 ± 0.1	0.9 ± 0.1*
			ALHF_06811.g4468	1.7 ± 0.1	0.9 ± 0.03*	0.6 ± 0.07*	1.2 ± 0.2	1.7 ± 0.1	0.8 ± 0.08*
			ALHF_07519.g4838	1.7 ± 0.07	1.1 ± 0.09	0.2 ± 0.06*	0.9 ± 0.2	1.6 ± 0.06	0.9 ± 0.04
			ALHF_02706.g1581	1.4 ± 0.1	0.52 ± 0.1*	0.37 ± 0.1*	1.2 ± 0.3	1.3 ± 0.04	1.2 ± 0.11
			ALHF_04422.g2918^††^	-	-	-	-	-	-
		Adenylate and Guanylate cyclase catalytic domain	ALHF_01050.g580	2.6 ± 0.3	1.3 ± 0.1	0.8 ± 0.09*	1.1 ± 0.2*	2.2 ± 0.21	1.3 ± 0.3*
			ALHF_07748.g4948	2.1 ± 0.2	1.2 ± 0.1	0.6 ± 0.05*	1.3 ± 0.2	1.8 ± 0.05	1.0 ± 0.2*
		Serpentine type 7TM GPCR chemoreceptor Srw	ALHF_01902.g1074	-	-	-	-	-	-
Intra-cellular processes	Proteases	Serpins	ALHF_07374.g4763	4.5 ± 0.8	1.8 ± 0.2	0.6 ± 0.2*	1.3 ± 0.1*	2.3 ± 0.2	0.5 ± 0.08*
			ALHF_01182.g646	1.7 ± 0.1	0.8 ± 0.07	0.3 ± 0.07*	1.1 ± 0.08	1.8 ± 0.1	0.7 ± 0.08*
		Carboxypeptidases	ALHF_04057.g2616	2.2 ± 0.3	1.5 ± 0.1	0.5 ± 0.1*	1.3 ± 0.2	1.8 ± 0.07	0.9 ± 0.1*
			ALHF_05871.g3981^††^	-	-	-	-	-	-
		Subtilase	ALHF_00530.g295^††^	-	-	-	-	-	-
		Aspartyl protease	ALHF_06529.g4317	2.5 ± 0.1	2.3 ± 0.2	1.4 ± 0.2*	1.3 ± 0.1*	2.1 ± 0.1	1.4 ± 0.01*
		Peptidases	ALHF_00761.g417	780 ± 50	530 ± 100	630 ± 60	23 ± 2*	650 ± 110	530 ± 20
			ALHF_03218.g1970	29 ± 2.1	30 ± 3	35 ± 5	2.6 ± 0.4*	17 ± 3*	8.2 ± 1*
			ALHF_02207.g1267	145 ± 10	150 ± 20	120 ± 3	5.0 ± 0.3*	4.6 ± 2*	4.9 ± 0.6*
			ALHF_07511.g4836	2.1 ± 0.1	1.5 ± 0.2	0.52 ± 0.04*	3.2 ± 0.2	0.52 ± 0.07*	0.41 ± 0.08*
			ALHF_01861.g1049^††^	-	-	-	-	-	-
			ALHF_05334.g3663	1.4 ± 0.04	1.0 ± 0.1	0.66 ± 0.06*	1.5 ± 0.1	0.75 ± 0.07*	0.40 ± 0.1*

^†^Structural Classification of Proteins (SCOP) http://supfam.cs.bris.ac.uk/SUPERFAMILY/.

^††^Autosomal interactions are named after the combination of the autosomes donated by the aabys strain in each case where a *M. domestica* autosomal combination line had a level of gene expression significantly lower than the ALHF strain at the α = 0.05 level of significance.

^††^Gene was not differentially-expressed between the aabys and ALHF strains.

^§^Gene predicted function as evidenced by the Pfam HMM-A homology (http://pfam.sanger.ac.uk/).

*Indicates that the gene expression value within a given *M. domestica* autosomal line was significantly lower than the expression in the parental ALHF strain at the α = 0.05 level of significance.

### Autosome co-regulation in up-regulation gene expression in resistant house flies

**Figure 8:**
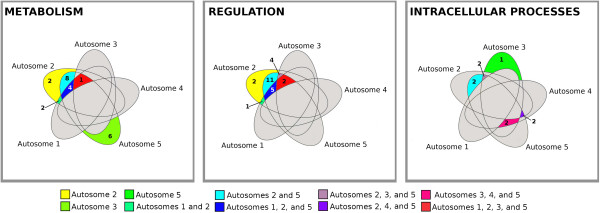
**Linkage of genes up-regulated in the ALHF strain of *Musca domestica.*** The overlapping areas between the ellipses indicate the autosomal interaction for those genes that were up-regulated in the ALHF strain for two or more of autosomes.

We next examined the autosomal linkage of factors from different autosomes on the 70 up-regulated genes that have been validated by qRT-PCR to determine the effects of the co-regulation on the expression of the up-regulated genes among five house fly lines of A2345, A1345, A1245, A1235 and A1234. Analyzing the gene expression changes resulting from autosome replacement in ALHF enabled us to evaluate the role of genes or factors on each autosome plays in gene overexpression in ALHF. We conducted Venn diagram analyses on the autosome interaction for the expression of genes in each of the SCOP general function categories of metabolism, regulation, and intracellular processes (Figure 8). The results revealed that apart from the 11 genes up-regulated solely by factor(s) on a single autosome (four in autosome 2, six in autosome 5 and one in autosome 3), the expression of the rest of the up-regulated genes were all linked to factors on more than one autosome (Figure 8). This result suggests that factors on different autosomes are capable of co-regulation of some genes. This was most commonly observed for autosomes 2 and 5. Almost one-third of the tested genes (n = 21 genes) were up-regulated by co-regulation of factors on autosome 2 and 5 only, including cytochrome P450s, GSTs, and esterases in metabolism; kinases/phosphatases, 7 transmembrane receptors (rhodopsin-like GPCR family), adenylate and guanylate cyclases in regulation; and serpin and carboxypeptidases in intracellular processes (Figure 8, Table 4). Nine genes were co-up-regulated by factors on autosomes 1, 2, and 5, with the functions of these genes being linked to metabolism and regulation categories, suggesting that factors on autosome 1, besides those on autosomes 2 and 5, were also involved in the regulation of some of the gene expression in metabolism and regulation. Six genes were up-regulated by the interaction of factors on autosomes 2, 3, and 5 and these genes were mainly located in the regulation and protease categories and none of the metabolic genes were involved in the interactions by factors among autosomes 2, 3, and 5, suggesting that, besides the factors on autosomes 2 and 5, factors on autosome 3 might have a role in the functions of regulation and proteolysis. A few genes were co-regulated by factors on autosomes 1 and 2 (including one P450 gene, one carboxylesterase gene, and one GPCR gene), or autosomes 1, 2, 3, and 5 (including one P450 gene and two protein kinase genes) (Table 4). No gene interactions between 1 and 3; 1 and 5; 2 and 3; 3 and 5; 1, 2 and 3; 1, 3 and 5; 2. 3, and 4 or 2, 3, 4 and 5 were observed. None of the genes were found to be up-regulated solely by factors on autosome 1 or 4 alone.

**Figure 9:**
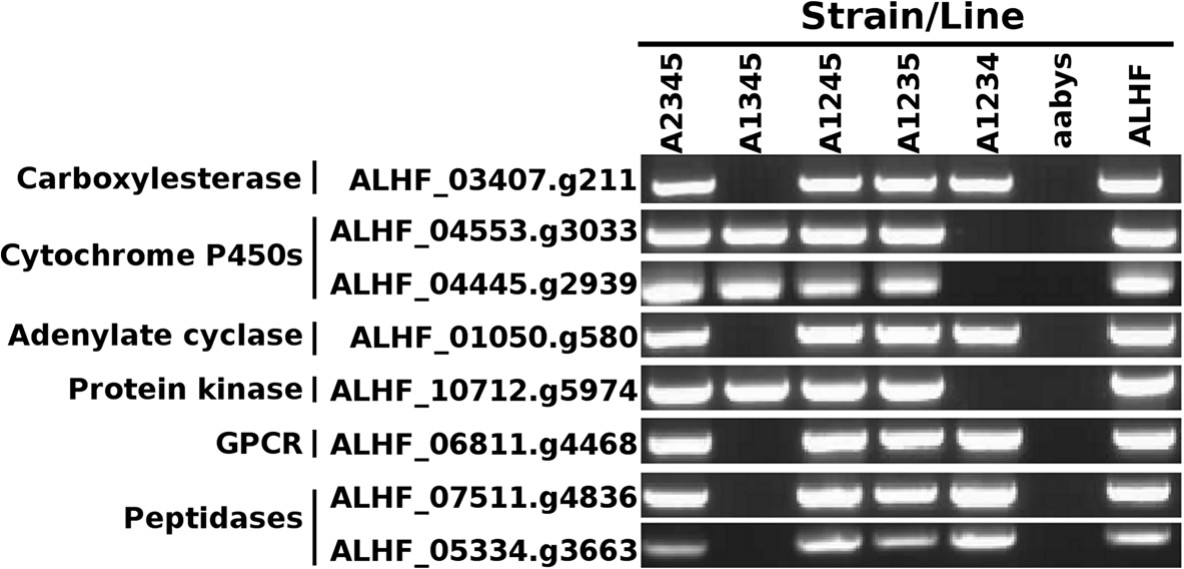
**Allele-specific RT-PCR autosomal mapping of the *Musca domestica *genes.** PCR fragments were generated using the allele-specific primer set according to the sequence of each gene from ALHF. The absence of a PCR product in a house fly line indicated that the gene was located on the corresponding autosome from aabys (i.e. the absence of a band in the A1234 line indicates that the gene was present on autosome 5).

To better understand the *cis/trans* regulation of the up-regulated genes in resistant house flies, autosomal location analyses were conducted for eight up-regulated genes scattered among all three important functional categories. An allele specific PCR (AS-PCR) determination was performed to examine the autosomal location of the genes with five house fly lines. The ALHF allele specific primer pair was designed based on the specific sequence of the genes from ALHF by placing a specific nucleotide polymorphism at the 3’ end of each primer to permit preferential amplification of specific alleles from ALHF. Our results showed that the ALHF allele-specific primer sets for P450 genes of ALHF_04445.g2939 (*CYP6A36*) and ALHF_04553.g3033 and protein kinase gene ALHF_10712.g5974 amplified specific DNA fragments only in flies having the autosome 5 wild-type marker from ALHF (Figure 9), which demonstrated that these three genes were located on autosome 5. Whereas, carboxylesterase gene ALHF_03407.g2111, adenylate cyclase gene ALHF_01050.g580, G-protein coupled receptor gene ALHF_06811.g4468, and peptidase genes ALHF_07511.g4836 and ALHF_05334.g3663 were located on autosome 2. These results were consistent with our autosomal linkage map (Figure 7, Table 4).

## Discussion

The central hypothesis guiding this research is that normal biological and physiological pathways and gene expression signatures are varied in resistant insects through changes in multiple gene expression, thus enabling resistant house flies to adapt to environmental or insecticidal stress, and that these changes are controlled by a regulatory network and perhaps by signaling transduction. This hypothesis is grounded in evidence from the considerable body of research that has been done in this field. Results from previous studies by ourselves [14,17,58-61], and others [8,10,12,16,62-73] all indicate that the interaction of multiple genes and complex mechanisms are responsible for the development of insecticide resistance in insects. Indeed, many studies have demonstrated different patterns of gene expression between resistant and susceptible insect populations and the up-regulation of P450 and GST genes in resistant insects. Many studies have also found that the overexpression of resistant metabolic genes is regulated by *trans* and/or *cis* factors in insecticide-resistant insects [8,74-83]. The up-regulation of a GST gene (GST-2) in the mosquito *Aedes aegypti* is controlled by a *trans*-acting factor [74], while the up-regulation of two P450 genes, *CYP6A1* and *CYP6D1*, in the house fly *M. domestica* are known to be *trans*-regulated by one or more factors on autosome 2 [10,75,81,82]. The up-regulation of *CYP6A2* and *CYP6A8* in the fruit fly *D. melanogaster* is transcriptionally regulated by *trans*-regulatory factors [76,77]. The up-regulation of *CYP6G1* and *CYP6D1* is controlled by *cis/trans* regulatory factors [81-84].

Taken together, these findings suggest that not only is insecticide resistance conferred via multi-resistance mechanisms or genes, but it is mediated through the interaction of regulatory genes and resistance genes such as P450s, esterases and GSTs. However, a global understanding of the complex processes resulting from gene interaction and regulation remains elusive. None of the regulatory factors responsible for insecticide resistance have yet been identified, and no regulation pathways have been examined. Nevertheless, these gaps will soon be filled following the availability of whole transcriptome analyses, which have begun to provide new ways of assessing how insects respond to the environment and insecticides [85].

To define the key genes and their *trans/cis*- or co-regulation involved in insecticide resistance, and thus gain fresh insights into the overall picture of how molecular mechanisms in resistant house flies function, we began by assembling and annotating the adult house fly transcriptome, providing the first reference transcriptome for adult house flies. Using the house fly transcriptome as a reference, our RNA-Seq of the resistant ALHF strain revealed a set of 1316 genes that were up-regulated relative to the susceptible aabys and CS strains, and a total of 1440 genes that were down-regulated. These results may not only reveal equally dynamic changes in abundance for both the increases and decreases in the total gene expression for different categories in resistant house flies, but also indicate an important feature of resistance gene regulation by both activators (the up-regulated genes) and perhaps, repressors (those down regulated genes). Several hypotheses have been proposed for the harmonizing of up- and down-regulation, e.g., homeostatic responses for protecting the cell from the harmful effects of oxidizing species from metabolic enzymes [86,87]; homeostatic responses to provocative processes [88]; and/or an essential for the tissue to utilize its transcriptional machinery and energy for the synthesis of other components involved in the inflammatory response [89]. Whether the down-regulated genes identified in the resistant house flies by our study reflects a regulation feature or homeostatic response of mosquitoes to insecticides needs to be further studied.

Deciphering the up-regulated genes among the SCOP general categories into detailed functions uncovered three key SCOP detailed function categories, namely the redox detailed function category in metabolism, signal transduction and kinases/phosphatases in regulation, and proteases in intra-cellular processes. The redox detailed function group contained a number of superfamilies that have been linked to detoxification, including multiple cytochrome P450s, glutathione S-transferases, and esterases. The signal transduction and kinases/phosphatases detailed function groups were found to contain several gene families with signal transduction and regulation functions, including 7 transmembrane receptors (rhodopsin-like GPCR family), adenylate and guanylate cyclases, protein kinases and phosphatases. The proteases detailed function group contained genes with digestive*,* catalytic, and proteinase activities. Since co-regulation provides valuable insights into altered categories/pathways, thereby aiding functional interpretation [90], this finding suggests that these co-up-regulated functional groups of genes may share co-regulation features. Among the three key detailed function categories, the roles of the detoxification superfamilies of P450s, GSTs and esterase in insecticide resistance have been extensively studied and up-regulation of their expression has been demonstrated to be associated with enhanced metabolic detoxification of insecticides, resulting in the development of insecticide resistance in insects [1,16,31,32,55,58,59,75],[82,91-99]. In contrast to the well-known role played by the detoxification system in insecticide resistance, the functions of genes in two other key detailed function categories, the signaling transduction system and proteases/serine proteases, such as GPCRs, protein kinase/phosphatases and proteases, in insecticide resistance are less well understood, although a few studies have reported the up-regulation of protease genes in insecticide resistant insects [16,61,100-103]. Nevertheless, the genes in these two key categories are well known as key intracellular signaling regulators and share common functions in the signaling pathway, playing an important role in transmitting information from extracellular polypeptide signals to target gene promoters in the nucleus and in the regulation of gene expression, activation/termination intracellular signaling transduction, and regulating numerous diverse cellular and biological/physiological processes [102,104-119].

To test the co-regulation of the up-regulated genes in these three key categories in resistant house flies, a novel approach was applied in the study, in which the gene expression profile in the house fly genetic lines was characterized in terms of different autosome combinations from the resistant ALHF strain, thus illustrating the co-regulation of autosomes in the expression of individual genes. This research approach not only provides a catalog of genes and information about their potential functions in insecticide resistance [120], but also serves as a stepping stone towards filling important gaps in our knowledge of transcriptional interaction and the regulation networks that are involved in insecticide resistance. Our gene co-regulation analysis revealed that the up-regulated gene expression in resistant ALHF house flies occurred primarily as a result of the co-regulation of factors between autosomes 2 and 5, although a few genes had their expression regulated by factors among autosomes 1, 2, and 5, or among autosomes 2, 3, and 5. These findings strongly suggest that multiple factor/autosome co-regulation, especially those related to autosomes 2 and 5, are key determinants for individual gene expression in resistant house flies. Among the up-regulated genes, cytochrome P450s, GSTs, and esterases in metabolism; kinases/phosphatases, 7 transmembrane receptors (rhodopsin-like GPCR family), adenylate and guanylate cyclases in regulation; and serpin and carboxypeptidases in intracellular processes as major groups of genes were up-regulated by the interactions of factors on autosomes 2 and 5 (Table 4). Our genetic mapping study further located two P450 genes and a protein kinase gene on autosome 5, and mapped a carboxylesterase gene, an adenylate cyclase gene, a G-protein coupled receptor gene and two peptidase genes on autosome 2. With the exception of one P450 gene, whose up-regulation was controlled by *cis* factor(s) on the same autosome on which the gene was located, all the genes tested in the genetic mapping study showed their expression being controlled by *cis* and *trans* factors. i.e., factors not only on the autosomes on which the genes were located but also other autosomes as well. Taken together, our findings suggested that that not only is insecticide resistance conferred via multi-resistance mechanisms or up-regulated genes, but it is mediated through the *trans* and/or *cis* co-regulations of resistance genes. Whether the signaling transduction cascades controlled by GPCRs, protein kinase/phosphatases and proteases are indeed involved in the regulation of resistance P450 genes and of resistance development remains an urgent topic for investigation.

## Conclusion

This study not only provides a catalog of genes that are co-up-regulated and information about their potential functions, but may also ultimately lead to a deeper understanding of transcriptional regulation and the interconnection of co-regulated genes, including metabolic genes, and those with catalytic activities, proteolytic activities and, perhaps, functions related to the regulation, signaling transduction, and protection of cells and tissues in resistant house flies. It has been suggested that co-overexpressed genes are frequently co-regulated. Therefore, characterizing these co-regulated genes as a whole will represent a good starting point for characterizing the transcriptional regulatory network and pathways in insecticide resistance, and improve our understanding of the dynamic, interconnected network of genes and their products that are responsible for processing environmental input, including the response to insecticide pressure and the regulation of the phenotypic output, in this case the insecticide resistance of insects. The new information presented here will provide fundamental new insights into the precise mechanisms by which insecticide resistance is regulated and how the genes involved are interconnected and regulated in resistance.

## Availability of supporting data

All sequence traces have been submitted to the National Center for Biotechnology Information (NCBI) Short Read Archive (SRA) as accessions [NCBI:SRR521286] (http://www.ncbi.nlm.nih.gov/sra/?term=SRR521286), [NCBI:SRR521288] (http://www.ncbi.nlm.nih.gov/sra/?term=SRR521288), [NCBI:SRR521289] (http://www.ncbi.nlm.nih.gov/sra/?term=SRR521289), and [NCBI:SRR521290] (http://www.ncbi.nlm.nih.gov/sra/?term=SRR521290), and are part of Bioproject #170716 (http://www.ncbi.nlm.nih.gov/bioproject/?term=170716) with additional information in the NCBI Gene Expression Omnibus [NCBI:GSE39327] (http://www.ncbi.nlm.nih.gov/geo/query/acc.cgi?acc=GSE39327). The nucleotide sequence information for house flies has been submitted to the NCBI Transcriptome Shotgun Assembly (TSA) (http://www.ncbi.nlm.nih.gov/genbank/tsa/) with accession numbers [NCBI:KA644422] through [NCBI:KA650580] (http://www.ncbi.nlm.nih.gov/nuccore/).

## Competing interests

The authors declared that they have no competing financial interests.

## Authors’ contributions

NL and LZ participated in the design and coordination of the study. ML, WRR, and LZ carried put the experiments and performed the data analysis. NL, LZ, JGS, and XG contributed reagents/materials/analysis tools. WRR, ML, NL, LZ, JGS, and MK wrote the paper. All authors read and approved the final manuscript.

## Supplementary Material

Additional file 1: Table S1 List and sequences of the primers used. Click here for file

Additional file 2: Table S2 The complete annotation spreadsheet for the *M. domestica* predicted gene set. Click here for file

Additional file 3: Table S3 Complete list of up-regulated and down-regulated genes in the ALHF *M. domestica* strain when compared to the pyrethroid-susceptible *M. domestica* strains aabys and CS. Click here for file
