# Supplementary material for: A whole transcriptomal linkage analysis of gene co-regulation in insecticide resistant house flies, Musca domestica
Source: BMC Genomics. 2013 Nov 19;14:803. doi: 10.1186/1471-2164-14-803 (PMC3870961; doi:10.1186/1471-2164-14-803)
Supplement: Additional file 1: Table S1 — List and sequences of the primers used. [file 1471-2164-14-803-S1.doc]

Table S1. List and sequences of the primers used.

| GENE | Forward primer (5'-3') | Reverse primer (5'-3') |
| --- | --- | --- |
| *Primers used for qPCR* | | |
| ALHF_03407.g2111 | GGCTTTTAGTACATCTGCTCGG | GCACTGGGTAGTGGAAGATTAG |
| ALHF_05628.g3847 | AAGACACGTCACTGCTAACC | CCAGGCCGAAGAAAGGTATT |
| ALHF_00771.g422 | AAGCTTGACGAGGCTTTAGG | CAGGATCACCTGCCGTAAAT |
| ALHF_04553.g3033 | ATGTGATTGGTCGCTGTGCCTTTG | TACTTCTCCACATCGGGCATGGTT |
| ALHF_05265.g3608 | GGCTTTCGATGTGACCTATGCCAA | TTGATGACACATTGCACACCAGCC |
| ALHF_03088.g1882 | TACATACCCGTGCCCGTCTTGAAT | CCTTGCAACGACTGGCTTCAAAGT |
| ALHF_02791.g1651 | TTGGCTTTGCATCAGGAGGTTCAG | AATCTTCGAGGGCGTGACGATTCA |
| ALHF_07553.g4857 | ACTGCTCGCAAATATCCTGGCCTA | TCCGGCTTATAATCCATGGGCTGA |
| ALHF_04445.g2939 | CCGAATACATTCAATCCCGATAAC | ATGATTCCTTGCTATAGGTCAGGG |
| ALHF_04444.g2938 | CGGATGTTTTTAATCCCGAACA | AAATCCTCTCCACACGCAAAT |
| ALHF_03006.g1816 | TATTGTACGCGAAACGGTGGCCTA | ATGATGAGGTCTCAAAGCCACCCA |
| ALHF_01822.g1025 | AATCGCCAAGCTGTCGAAGACTAC | TCCGGATTGAAGACATTGGGTTGG |
| ALHF_04730.g3176 | ATGCCATACACCATGATCCCGAGT | TAGTGTCGCAGCAGGGATACCAAA |
| ALHF_03063.g1860 | GTGCAGGATAAAGTGCGCCAAGAA | CTTTGACACACATGCGCACCAGAT |
| ALHF_05136.g3505 | TGACTGCTGGCTTTGAGACATCCT | ATTTCTCGACGCAAACGTTCCTGC |
| ALHF_07623.g4891 | TGCTCTCTATGAATTGGCCCGGAA | AGGACGGCATATTTGCGTAGGGTT |
| ALHF_08221.g5182 | TTAAGACCACCCTGCTGTGGATCA | GTGGCCAATGGTACAATGCTGGAA |
| ALHF_04665.g3125 | TTTCCCGAACCGGAGAAGTTCGAT | GCATTTCAGCTCCAACATGGCGAA |
| ALHF_01339.g731 | ATACAACCGCTGCTGGATCTTCGT | ACCGGTGGATACATTCTCAGCGTT |
| ALHF_04736.g3182 | AGTCTCTTCGCATTCATCCCTT | CATTCTCCGTGGGATAGTCAAA |
| ALHF_03849.g2446 | TACACCACCGAAGTTGTCAGCGAT | TCATCACCTTCTTTAGGCTGGGCA |
| ALHF_04900.g3328 | GATGTCCATAAACGTGCCAAAG | CCTCGCCGACATGGATTAAA |
| ALHF_04476.g2964 | ACCTCGTTGGTGGCATTT | ACCTGCTCCATTAGCTTCTTC |
| ALHF_03731.g2351 | CGTAGCCAGTACGGATTACTTC | GTTGTTTAGCAGCTCCGTTTAC |
| ALHF_04477.g2965 | GGCGGTCTACGCAACATTA | CCAGGAAACCATAACTCTCTACAA |
| ALHF_03145.g1917 | GGACCTGCAAGAACGTAGAAA | TCTCTGTGACTGTTGGCATTC |
| ALHF_02546.g1487 | GTACTGCATGTCTGGCATTTG | CAACGATATGGGCGAGATGA |
| ALHF_00685.g381 | AACTCCAAGGGCCGTAAAC | CCCATTCCAGACAACGATAGAG |
| ALHF_03462.g2147 | GTCGTCGACATCATCACACA | GTTAGAATACCGCCAGGGTTAG |
| ALHF_02885.g1722 | GGCCGATACACTTAAGGAAAGA | CCGCCTCACATAGCGTAAATA |
| ALHF_00823.g452 | CGATTGTGGCATAGAGACCAA | CTCCTCATCCTCCTCGGTAATA |
| ALHF_04500.g2986 | GAATGAGGAGAGTGGGTCTTTG | GTTCCTCCCAGCGTTCTATG |
| ALHF_04095.g2646 | CTATCGTGCACCCGAATTGA | ACCAGAGTCACCAGGGAATA |
| ALHF_01595.g882 | GCGATACCTTCTTCCTCATCTC | CAGGGTGCGTATTTCCTTCT |
| ALHF_01832.g1033 | CTGATGAGATACGCCTCCTAGA | GATTGAGCAAACGCACGATAC |
| ALHF_08078.g5122 | AGGGTACTTTGGTGCATAAGG | CAGGGCAGACAATGGAATCT |
| ALHF_11277.g6269 | GAGTGCGATTGTGGTTGTTTG | GCCAGTACTGCTGCTGTTATTA |
| ALHF_11442.g6384 | GCCAAAGTCAGACCAAAGATATG | TTGCGTTCGGCCAATAGA |
| ALHF_00727.g395 | TGAGAAGACGGCACGATTTAT | CTCCGGTTTCAAGTCTCTGTAG |
| ALHF_11829.g6650 | CCCGATGATCAGGGTCAATATAA | CCCAATGTAAGGCCAGGAATA |
| ALHF_11144.g6194 | GTAATGGCGGTAGTCCACATAAA | GCCATCCAACGTATGGGTAAA |
| ALHF_09312.g5609 | GCGAGGTCTATGAGGGTTTG | CCTTGCGGAGGGTCTTTATT |
| ALHF_10712.g5974 | CTGCCAGAAACGTGCTAGT | GCCTCGTATTCATCCTCCTTTAT |
| ALHF_07173.g4665 | GGTAAAGGTCACCGATGAGATAC | CACTGGCTCCCTTGACAATAA |
| ALHF_03649.g2289 | CGGAAACCGGAAGTAGAGATAC | CGACACCATTGCCACTATCTA |
| ALHF_05773.g3933 | TGCGCCTGAGATCATACTTTATC | CGGTTGGCCTACTAGCATTT |
| ALHF_11245.g6252 | GGTGGTGGTAGTCCACATAAA | CCATCCAACGTATTGGCAAAG |
| ALHF_11768.g6612 | TGTGCCCAGTTCAGCTATG | GCCCATGTATCTCCCAATGAT |
| ALHF_03863.g2457 | CAGTCCGACATCGACAACAA | TACCAGTGGCCGTATCTCTATC |
| ALHF_01760.g986 | CGCTTGCGGTACGGATTATT | GTAGACAGCGGTGTCAGATAGA |
| ALHF_02400.g1393 | AACCAGACCCGACCTCATA | GACTGCTCACAGAACGACATAG |
| ALHF_06811.g4468 | CTCTCCTTCCTGCCCATATTC | CGAACTGGAGATGACACAGTAG |
| ALHF_07519.g4838 | TATGCTACGGTGTCGCTATTG | GGGATCCAAGCAGGAAACTAA |
| ALHF_02706.g1581 | GACTGGAATCCACGTACCTATTT | GCTTTCTCGTGAGCAGATACA |
| ALHF_04422.g2918 | CCACGTTCCTACTTGATCTCATAC | CAGAGACTGCAGCGATGATAAA |
| ALHF_01050.g580 | CCTGACTGGCATCTCGAATATC | CTGATGGCACAACAGAAGAGA |
| ALHF_07748.g4948 | CTGTGGTCTTGGCCCTATTT | CACTGGCCAGCATTACAATTTC |
| ALHF_01902.g1074 | CAATTTCCTCTCCGCCCTAAA | TCCCGTTATCAGCTCCCTAA |
| ALHF_07374.g4763 | GCAGCCGGAAGACTTTAAGA | GCATTGACTCCGAACGAGAT |
| ALHF_01182.g646 | CGTTCGGGTCTGTACGATTT | CCCGAGCATCCATTTCCTTTA |
| ALHF_04057.g2616 | TTTGCTCACAGCTACCCATAC | CCGGCACAGCAGTCTTAAT |
| ALHF_05871.g3981 | ATTGCTGACCTCCACAGATG | GTGGTGTGGGTGTAGACAAA |
| ALHF_00530.g295 | GCAAAACTGTGGATGGACCT | GAGCGTTTTGACCATCGTTT |
| ALHF_06529.g4317 | GATCCCTTGGCCTCCTCTAC | CATGCCAAATATGCCATCAA |
| ALHF_00761.g417 | CATGGCCACTACTCTGCTCA | ACTTGGAGCTGCATGGTTTC |
| ALHF_03218.g1970 | GCAAGAAATGCCAAAAATCG | TTCAATTGCAAATCGGCATA |
| ALHF_02207.g1267 | AAGTTGTGGGCGTACATTCC | ACAAATGGGTGGCCATAAAA |
| ALHF_07511.g4836 | GGCGAAAACATAGCCGATAA | ATCGGTGATGGTGACCAAAT |
| ALHF_01861.g1049 | TGTTCAACGCGTCTTCTACG | TCGATTTGAACCAACGATGA |
| ALHF_05334.g3663 | GGTGGCATTATGGCTGGTAT | TGCCAACACGTACACGAACT |
| ALHF_04132.g2678 (Actin) | ATGAGGCTCAGAGCAAACGTGGTA | AGTCATCTTCTCGCGATTGGCCTT |
| *Primers used for autosome mapping* | | |
| ALHF_03407.g2111 | F1: TTGAAATGTCCCAATTTGGA | R: AGAGCATATCCCAAACTATAATC |
|  | F2: TTATGACTCGGCATCCAAGA |  |
| ALHF_04553.g3033 | F1: ATGTGATTGGTCGCTGTGCCTTTG | R: CATACCCTTGCGTATGACATA |
|  | F2: CCCAAATCGGAATTCCGC |  |
| ALHF_04445.g2939 | F1:TTTAAGGGGTCTCTCGACGAGT | R: CAATGGAAACATGGCCTTCAT |
|  | F2:TTATGAGAAATATAGAAATTCCGCC |  |
| ALHF_01050.g580 | F1: CCTGTGCTGTGTTTAGTGTGA | R: CGTGAGCGGGTAGAATGTT |
|  | F2: CTGCAGATAGCTCCGATATATCTC |  |
| ALHF_10712.g5974 | F1: TTATGAAGCACGGTTCGC | R: GTGCCAGCATTCCAACAT |
|  | F2: GGCTTACTTGGAATCCCAG |  |
| ALHF_06811.g4468 | F1: CGGCATCATCATCACCAT | R: ACATCGGCATCGTAGGACA |
|  | F2: GCAACAAGAAGTACAACAAAATGTT |  |
| ALHF_07511.g4836 | F1: TCCTTTGGGACGATGTCT | R: CTCGCACACGAACTTCTTC |
|  | F2: GATGCCAAGGGCAATCTT |  |
| ALHF_05334.g3663 | F1: GCACAGTCCAAGTGGCTAA | R: GAGCGACAAATCTTCCAGA |
|  | F2: TTAAATTTGCCAGTGGTCTATTCC |  |
