# Supplementary material for: A whole transcriptomal linkage analysis of gene co-regulation in insecticide resistant house flies, Musca domestica
Source: BMC Genomics. 2013 Nov 19;14:803. doi: 10.1186/1471-2164-14-803 (PMC3870961; doi:10.1186/1471-2164-14-803)
Supplement: Additional file 3: Table S3 — Complete list of up-regulated and down-regulated genes in the ALHF M. domestica strain when compared to the pyrethroid-susceptible M. domestica strains aabys and CS. [file 1471-2164-14-803-S3.doc]

Table S3. Complete list of up-regulated and down-regulated genes in the ALHF *M. domestica* strain when compared to the pyrethroid-susceptible *M. domestica* strains aabys and CS.

| **SCOP^†^ Functional annotation** | | ***M. domestica* predicted gene function and gene accession number** | | **Expression level (FPKM)*** | | |
| --- | --- | --- | --- | --- | --- | --- |
| **General** | **Detailed** | **Pfam annotation^§^** | **Gene** | **ALHF** | **aabys** | **CS** |
| UP-REGULATED GENES | | |  |  |  |  |
| Extra-cellular processes | Blood clotting | Fibrinogen beta and gamma chains, C-terminal globular domain | ALHF_00373.g191 | 1190 | 989 | 420 |
|  |  |  | ALHF_00712.g389 | 100 | 23 | 16 |
|  |  |  | ALHF_04391.g2892 | 258 | 32 | 28 |
|  |  |  | ALHF_05495.g3771 | 765 | 525 | 71 |
|  |  |  | ALHF_11206.g6230 | 25 | 12 | 9 |
|  | Cell adhesion | Cell adhesion | ALHF_07013.g4576 | 128 | 89 | 97 |
|  |  |  | ALHF_08050.g5104 | 480 | 154 | 34 |
|  |  | Calcium-binding EGF domain | ALHF_08641.g5372 | 100 | 45 | 22 |
|  |  | CD80-like C2-set immunoglobulin domain | ALHF_04802.g3242 | 327 | 284 | 96 |
|  |  | CHRD domain | ALHF_04671.g3131 | 1061 | 916 | 349 |
|  |  | Collagen triple helix repeat (20 copies) | ALHF_04908.g3335 | 4613 | 1248 | 64 |
|  |  |  | ALHF_05207.g3564 | 15044 | 14178 | 3417 |
|  |  |  | ALHF_05305.g3639 | 11237 | 9026 | 2342 |
|  |  | E2 domain of amyloid precursor protein | ALHF_02050.g1163 | 991 | 666 | 196 |
|  |  | F-box-like | ALHF_04909.g3336 | 2486 | 1086 | 291 |
|  |  | Fasciclin domain | ALHF_03954.g2538 | 460 | 413 | 136 |
|  |  |  | ALHF_04731.g3177 | 152 | 0 | 14 |
|  |  |  | ALHF_04732.g3178 | 288 | 0 | 121 |
|  |  |  | ALHF_11730.g6583 | 152 | 27 | 7 |
|  |  | Fibrillar collagen C-terminal domain | ALHF_03829.g2429 | 434 | 126 | 7 |
|  |  | Fibronectin type III domain | ALHF_04515.g3000 | 1712 | 657 | 247 |
|  |  |  | ALHF_09563.g5685 | 160 | 127 | 48 |
|  |  | GCC2 and GCC3 | ALHF_04376.g2879 | 5793 | 1577 | 246 |
|  |  | Immunoglobulin I-set domain | ALHF_06499.g4301 | 763 | 744 | 139 |
|  |  |  | ALHF_03799.g2405 | 7639 | 2762 | 1493 |
|  |  |  | ALHF_03834.g2434 | 13571 | 9042 | 6558 |
|  |  |  | ALHF_03835.g2435 | 7151 | 2900 | 684 |
|  |  | Integrin alpha | ALHF_11380.g6347 | 201 | 169 | 17 |
|  |  | Lectin C-type domain | ALHF_05247.g3595 | 236 | 16 | 5 |
|  |  |  | ALHF_05269.g3612 | 1474 | 754 | 163 |
|  |  | Leucine Rich repeat | ALHF_00420.g222 | 470 | 395 | 137 |
|  |  |  | ALHF_03525.g2187 | 260 | 247 | 130 |
|  |  |  | ALHF_05020.g3416 | 1586 | 972 | 453 |
|  |  |  | ALHF_07002.g4569 | 253 | 198 | 241 |
|  |  |  | ALHF_10894.g6064 | 26 | 0 | 0 |
|  |  |  | ALHF_02409.g1397 | 2837 | 2113 | 1231 |
|  |  | SEA domain | ALHF_05500.g3773 | 4211 | 2090 | 1429 |
|  |  | Sec23/Sec24 trunk domain | ALHF_11415.g6367 | 256 | 42 | 53 |
|  |  | Sema domain | ALHF_06183.g4135 | 608 | 482 | 124 |
|  |  | Spectrin repeat | ALHF_11586.g6488 | 1672 | 1462 | 592 |
|  |  | Spondin_N | ALHF_07638.g4894 | 177 | 149 | 81 |
|  |  | Tropomodulin | ALHF_10477.g5897 | 1300 | 540 | 229 |
|  |  |  | ALHF_10725.g5982 | 626 | 377 | 73 |
|  |  | Uncharacterised conserved protein (DUF2369) | ALHF_08630.g5368 | 37 | 13 | 5 |
|  |  | Vinculin family | ALHF_09043.g5510 | 201 | 187 | 162 |
|  | Immune response | Fibroblast growth factor | ALHF_07545.g4852 | 26 | 8 | 4 |
|  |  | Sushi domain (SCR repeat) | ALHF_00836.g460 | 213 | 111 | 69 |
|  |  |  | ALHF_10277.g5848 | 470 | 258 | 106 |
|  |  |  | ALHF_10962.g6087 | 413 | 148 | 154 |
|  |  | Tetraspanin family | ALHF_02726.g1600 | 127 | 32 | 7 |
|  |  |  | ALHF_03317.g2044 | 184 | 110 | 54 |
|  |  |  | ALHF_03590.g2241 | 188 | 182 | 58 |
|  | Toxins/defense | Activin types I and II receptor domain | ALHF_01004.g552 | 113 | 53 | 8 |
|  |  |  | ALHF_04519.g3004 | 272 | 96 | 70 |
|  |  | PA26 p53-induced protein (sestrin) | ALHF_05407.g3712 | 2189 | 1216 | 1111 |
| General | General | General | ALHF_02748.g1618 | 184 | 99 | 29 |
|  |  |  | ALHF_04884.g3314 | 1160 | 685 | 264 |
|  |  | Calponin homology (CH) domain | ALHF_05168.g3528 | 2170 | 1554 | 983 |
|  |  |  | ALHF_11664.g6545 | 823 | 0 | 424 |
|  |  | CLASP N terminal | ALHF_02330.g1352 | 472 | 0 | 0 |
|  |  | Cytoplasmic dynein 1 intermediate chain 2 | ALHF_05502.g3774 | 1234 | 873 | 726 |
|  |  | DDHD domain | ALHF_03287.g2018 | 629 | 608 | 344 |
|  |  | EF hand | ALHF_01109.g614 | 92 | 49 | 32 |
|  |  |  | ALHF_02226.g1282 | 27076 | 3457 | 682 |
|  |  |  | ALHF_02418.g1402 | 1662 | 762 | 366 |
|  |  |  | ALHF_03219.g1972 | 108 | 103 | 28 |
|  |  |  | ALHF_04219.g2750 | 290 | 137 | 74 |
|  |  |  | ALHF_05124.g3496 | 8066 | 2268 | 869 |
|  |  |  | ALHF_05203.g3561 | 12488 | 6393 | 1305 |
|  |  |  | ALHF_06092.g4099 | 108 | 103 | 28 |
|  |  | EF-hand domain | ALHF_05505.g3775 | 623 | 345 | 153 |
|  |  | EF-hand domain pair | ALHF_00521.g288 | 16506 | 4857 | 1692 |
|  |  |  | ALHF_00522.g289 | 48917 | 7766 | 1904 |
|  |  |  | ALHF_01326.g724 | 316 | 26 | 102 |
|  |  |  | ALHF_01956.g1104 | 147 | 119 | 17 |
|  |  |  | ALHF_01982.g1118 | 183 | 128 | 63 |
|  |  |  | ALHF_05225.g3577 | 128671 | 18613 | 5378 |
|  |  |  | ALHF_05978.g4043 | 359 | 280 | 228 |
|  |  |  | ALHF_07454.g4811 | 1067 | 570 | 283 |
|  |  |  | ALHF_09166.g5559 | 264 | 51 | 26 |
|  |  | Filamin/ABP280 repeat | ALHF_11635.g6525 | 1347 | 1258 | 534 |
|  |  |  | ALHF_11810.g6636 | 1344 | 1171 | 272 |
|  |  | HEAT repeat | ALHF_08362.g5249 | 2178 | 758 | 378 |
|  |  | Kelch motif | ALHF_04292.g2814 | 5914 | 1171 | 1366 |
|  |  | Leucine rich repeat | ALHF_00720.g392 | 982 | 630 | 112 |
|  |  |  | ALHF_01886.g1064 | 185 | 58 | 13 |
|  |  |  | ALHF_05654.g3862 | 489 | 453 | 16 |
|  |  |  | ALHF_06905.g4518 | 562 | 87 | 26 |
|  |  |  | ALHF_07700.g4925 | 106 | 94 | 77 |
|  |  |  | ALHF_07952.g5052 | 250 | 74 | 4 |
|  |  |  | ALHF_08696.g5393 | 205 | 173 | 110 |
|  |  | Low-density lipoprotein receptor domain class A | ALHF_04105.g2655 | 952 | 835 | 298 |
|  |  | N-acetylmuramoyl-L-alanine amidase | ALHF_01082.g598 | 133 | 61 | 27 |
|  |  | p25-alpha | ALHF_05468.g3751 | 597 | 430 | 495 |
|  |  | PP1-regulatory protein, Phostensin N-terminal | ALHF_04214.g2745 | 239 | 149 | 120 |
|  |  | Protein of unknown function (DUF3585) | ALHF_04122.g2669 | 4085 | 3683 | 4063 |
|  |  | Protein phosphatase 2A regulatory B subunit (B56 family) | ALHF_04331.g2851 | 830 | 606 | 233 |
|  |  | Receptor L domain | ALHF_07349.g4747 | 414 | 379 | 315 |
|  |  | Secreted protein acidic and rich in cysteine Ca binding region | ALHF_01857.g1046 | 717 | 712 | 364 |
|  |  | Spectrin repeat | ALHF_04563.g3043 | 6361 | 5944 | 2510 |
|  |  |  | ALHF_05042.g3431 | 19212 | 3224 | 1024 |
|  |  |  | ALHF_05043.g3432 | 19212 | 3224 | 1024 |
|  |  |  | ALHF_11502.g6433 | 3080 | 0 | 0 |
|  |  |  | ALHF_11666.g6547 | 4364 | 3928 | 1691 |
|  |  | Synaptobrevin | ALHF_10631.g5950 | 368 | 209 | 162 |
|  |  | WD domain, G-beta repeat | ALHF_03059.g1856 | 600 | 450 | 479 |
|  |  |  | ALHF_04660.g3121 | 1919 | 1457 | 804 |
|  | Protein interaction | Protein interaction | ALHF_11053.g6139 | 108 | 56 | 18 |
|  |  |  | ALHF_11352.g6327 | 465 | 127 | 30 |
|  |  |  | ALHF_11353.g6328 | 465 | 127 | 30 |
|  |  |  | ALHF_11354.g6329 | 465 | 127 | 30 |
|  |  |  | ALHF_09534.g5676 | 41 | 10 | 6 |
|  |  | Ankyrin repeats (3 copies) | ALHF_05908.g4005 | 1343 | 1173 | 457 |
|  |  |  | ALHF_11617.g6508 | 410 | 67 | 171 |
|  |  | BAR domain | ALHF_02112.g1205 | 1618 | 1190 | 375 |
|  |  | BTB/POZ domain | ALHF_08069.g5116 | 128 | 53 | 20 |
|  |  |  | ALHF_04485.g2973 | 266 | 57 | 153 |
|  |  | Domain of unknown function (DUF3474) | ALHF_11284.g6275 | 17 | 0 | 0 |
|  |  |  | ALHF_11285.g6276 | 13 | 0 | 0 |
|  |  |  | ALHF_11287.g6278 | 43 | 7 | 0 |
|  |  |  | ALHF_11289.g6280 | 17 | 0 | 0 |
|  |  |  | ALHF_11290.g6281 | 13 | 0 | 0 |
|  |  |  | ALHF_11291.g6282 | 43 | 7 | 0 |
|  |  | Hemopexin | ALHF_08319.g5229 | 139 | 128 | 41 |
|  |  |  | ALHF_11207.g6231 | 96 | 52 | 0 |
|  |  | Human growth factor-like EGF | ALHF_11249.g6254 | 946 | 512 | 41 |
|  |  |  | ALHF_11496.g6427 | 685 | 0 | 41 |
|  |  | IRSp53/MIM homology domain | ALHF_04787.g3229 | 2124 | 528 | 138 |
|  |  | Phorbol esters/diacylglycerol binding domain (C1 domain) | ALHF_11540.g6460 | 3256 | 833 | 264 |
|  |  | Protein of unknown function (DUF1390) | ALHF_13403.g6810 | 41 | 25 | 6 |
|  |  | Respiratory-chain NADH dehydrogenase 51 Kd subunit | ALHF_00578.g328 | 19451 | 14032 | 8118 |
|  |  | Tetratricopeptide repeat | ALHF_03230.g1982 | 282 | 228 | 176 |
|  |  | Uncharacterized protein conserved in bacteria (DUF2317) | ALHF_11619.g6510 | 142 | 0 | 0 |
|  | Small molecule binding | Small molecule binding | ALHF_02950.g1770 | 232 | 173 | 76 |
|  |  |  | ALHF_05589.g3828 | 790 | 536 | 391 |
|  |  | ABC transporter | ALHF_11429.g6377 | 7 | 0 | 0 |
|  |  | ABC transporter transmembrane region | ALHF_04678.g3136 | 1424 | 356 | 228 |
|  |  |  | ALHF_06689.g4403 | 833 | 774 | 362 |
|  |  |  | ALHF_07498.g4831 | 758 | 544 | 216 |
|  |  |  | ALHF_08346.g5242 | 437 | 413 | 225 |
|  |  | ABC-2 family transporter protein | ALHF_01463.g800 | 604 | 458 | 276 |
|  |  |  | ALHF_05545.g3803 | 1408 | 1304 | 938 |
|  |  | ABC-2 type transporter | ALHF_00260.g124 | 285 | 165 | 52 |
|  |  |  | ALHF_02645.g1546 | 460 | 354 | 122 |
|  |  |  | ALHF_03595.g2245 | 2108 | 1857 | 656 |
|  |  |  | ALHF_03716.g2339 | 963 | 788 | 235 |
|  |  |  | ALHF_06273.g4181 | 383 | 184 | 61 |
|  |  |  | ALHF_07468.g4821 | 72 | 58 | 48 |
|  |  | Adenylate kinase | ALHF_00328.g165 | 3559 | 1361 | 239 |
|  |  |  | ALHF_03638.g2279 | 258 | 238 | 186 |
|  |  | AFG1-like ATPase | ALHF_04659.g3120 | 1309 | 860 | 487 |
|  |  | ATP synthase alpha/beta family, nucleotide-binding domain | ALHF_04852.g3286 | 102482 | 48942 | 27359 |
|  |  |  | ALHF_05472.g3755 | 2124 | 1901 | 355 |
|  |  | D-isomer specific 2-hydroxyacid dehydrogenase, NAD binding domain | ALHF_05110.g3488 | 5355 | 1705 | 654 |
|  |  |  | ALHF_05625.g3846 | 566 | 411 | 297 |
|  |  | Dehydrogenase E1 component | ALHF_02464.g1433 | 12413 | 6016 | 3007 |
|  |  |  | ALHF_04399.g2899 | 39061 | 37480 | 14850 |
|  |  | Deoxynucleoside kinase | ALHF_03370.g2083 | 10955 | 5446 | 3193 |
|  |  | Dynamin central region | ALHF_11449.g6390 | 931 | 722 | 408 |
|  |  |  | ALHF_11692.g6558 | 931 | 722 | 408 |
|  |  | Dynamin family | ALHF_11221.g6239 | 7489 | 1980 | 611 |
|  |  | Elongation factor Tu GTP binding domain | ALHF_03890.g2480 | 9337 | 2460 | 2096 |
|  |  | Enoyl-(Acyl carrier protein) reductase | ALHF_03563.g2218 | 345 | 231 | 122 |
|  |  | FAD binding domain | ALHF_01471.g806 | 17575 | 11014 | 6666 |
|  |  | FAD dependent oxidoreductase | ALHF_00362.g185 | 30402 | 9993 | 1751 |
|  |  |  | ALHF_05044.g3433 | 1514 | 1371 | 1304 |
|  |  | FAD linked oxidases, C-terminal domain | ALHF_01516.g831 | 144 | 27 | 61 |
|  |  |  | ALHF_07072.g4615 | 362 | 250 | 108 |
|  |  | Flavin-binding monooxygenase-like | ALHF_04913.g3338 | 743 | 321 | 139 |
|  |  |  | ALHF_05421.g3720 | 1150 | 1022 | 324 |
|  |  | Formate--tetrahydrofolate ligase | ALHF_02029.g1150 | 13377 | 10471 | 3663 |
|  |  | Fungal family of unknown function (DUF1776) | ALHF_00924.g515 | 407 | 335 | 171 |
|  |  | GMC oxidoreductase | ALHF_03437.g2127 | 769 | 501 | 234 |
|  |  |  | ALHF_04035.g2599 | 6611 | 3694 | 566 |
|  |  | Guanylate kinase | ALHF_01368.g746 | 364 | 306 | 119 |
|  |  |  | ALHF_07164.g4659 | 214 | 52 | 24 |
|  |  |  | ALHF_11598.g6495 | 215 | 9 | 72 |
|  |  | Kinesin motor domain | ALHF_04294.g2816 | 2224 | 1627 | 1095 |
|  |  | Male sterility protein | ALHF_00195.g98 | 85 | 19 | 5 |
|  |  |  | ALHF_01523.g835 | 360 | 106 | 33 |
|  |  |  | ALHF_05686.g3882 | 1040 | 434 | 381 |
|  |  |  | ALHF_07531.g4845 | 30 | 6 | 1 |
|  |  | Myosin head (motor domain) | ALHF_02436.g1413 | 693 | 475 | 159 |
|  |  |  | ALHF_10849.g6039 | 526 | 291 | 341 |
|  |  |  | ALHF_10850.g6040 | 375 | 210 | 0 |
|  |  | Myosin tail | ALHF_02437.g1414 | 530 | 197 | 36 |
|  |  |  | ALHF_02438.g1415 | 1296 | 456 | 97 |
|  |  |  | ALHF_02955.g1774 | 541943 | 55319 | 14654 |
|  |  |  | ALHF_02956.g1775 | 217912 | 27002 | 14957 |
|  |  |  | ALHF_03077.g1871 | 418 | 364 | 103 |
|  |  |  | ALHF_10851.g6041 | 305 | 84 | 78 |
|  |  |  | ALHF_10852.g6042 | 687 | 183 | 198 |
|  |  |  | ALHF_10853.g6043 | 215 | 55 | 0 |
|  |  |  | ALHF_10854.g6044 | 512 | 121 | 0 |
|  |  | NAD dependent epimerase/dehydratase family | ALHF_05237.g3588 | 10964 | 6016 | 2979 |
|  |  |  | ALHF_10312.g5854 | 1443 | 50 | 30 |
|  |  | Oxysterol-binding protein | ALHF_11499.g6430 | 334 | 145 | 164 |
|  |  | Phosphatidylethanolamine-binding protein | ALHF_03050.g1850 | 735 | 321 | 71 |
|  |  |  | ALHF_05523.g3787 | 360 | 131 | 88 |
|  |  | Pyridine nucleotide-disulphide oxidoreductase, dimerisation domain | ALHF_05135.g3504 | 20657 | 11255 | 5707 |
|  |  | Ras family | ALHF_01895.g1070 | 138 | 13 | 14 |
|  |  |  | ALHF_04169.g2712 | 140 | 129 | 50 |
|  |  |  | ALHF_04170.g2713 | 140 | 129 | 50 |
|  |  |  | ALHF_06813.g4470 | 114 | 85 | 44 |
|  |  |  | ALHF_07599.g4880 | 128 | 89 | 40 |
|  |  |  | ALHF_08249.g5195 | 97 | 91 | 55 |
|  |  |  | ALHF_09506.g5666 | 207 | 120 | 47 |
|  |  | Saccharopine dehydrogenase | ALHF_03856.g2450 | 233 | 102 | 41 |
|  |  | short chain dehydrogenase | ALHF_01284.g704 | 140 | 42 | 54 |
|  |  |  | ALHF_02394.g1389 | 133 | 17 | 7 |
|  |  |  | ALHF_05231.g3582 | 23501 | 16054 | 5897 |
|  |  |  | ALHF_05426.g3725 | 995 | 0 | 5 |
|  |  |  | ALHF_05533.g3794 | 710 | 333 | 107 |
|  |  |  | ALHF_06709.g4411 | 229 | 156 | 106 |
|  |  |  | ALHF_06859.g4498 | 193 | 94 | 22 |
|  |  |  | ALHF_11000.g6113 | 103 | 76 | 10 |
|  |  |  | ALHF_02311.g1342 | 180 | 156 | 78 |
|  |  |  | ALHF_11095.g6162 | 110 | 106 | 22 |
|  |  |  | ALHF_11122.g6180 | 155 | 67 | 28 |
|  |  | Sulfotransferase domain | ALHF_02783.g1643 | 504 | 215 | 264 |
|  |  |  | ALHF_02863.g1710 | 305 | 216 | 160 |
|  |  |  | ALHF_03297.g2027 | 277 | 231 | 35 |
|  |  |  | ALHF_10970.g6093 | 504 | 215 | 264 |
|  |  | Sushi domain (SCR repeat) | ALHF_01488.g817 | 631 | 220 | 24 |
|  |  | Tetrahydrofolate dehydrogenase/cyclohydrolase, NAD(P)-binding domain | ALHF_11497.g6428 | 2750 | 1491 | 808 |
|  |  | Thrombospondin type 3 repeat | ALHF_02795.g1652 | 1272 | 310 | 50 |
|  |  |  | ALHF_03347.g2067 | 1627 | 432 | 140 |
|  |  |  | ALHF_10977.g6098 | 7386 | 1500 | 542 |
|  |  |  | ALHF_11222.g6240 | 1548 | 326 | 77 |
|  |  | Transketolase, pyrimidine binding domain | ALHF_02705.g1580 | 10480 | 6285 | 3080 |
|  |  |  | ALHF_03879.g2469 | 517 | 368 | 220 |
|  |  |  | ALHF_03880.g2470 | 517 | 368 | 220 |
|  |  | Zinc ribbon domain | ALHF_00076.g47 | 55 | 31 | 18 |
| Information | Chromatin structure | Chromatin structure | ALHF_00563.g320 | 1069 | 399 | 265 |
|  | DNA replication/repair |  | ALHF_08897.g5463 | 231 | 225 | 222 |
|  |  | Endonuclease/Exonuclease/phosphatase family | ALHF_03246.g1995 | 1636 | 1579 | 822 |
|  |  | IBR domain | ALHF_03158.g1927 | 682 | 134 | 0 |
|  |  |  | ALHF_03159.g1928 | 569 | 193 | 0 |
|  |  |  | ALHF_10588.g5937 | 1516 | 288 | 469 |
|  |  |  | ALHF_10589.g5938 | 1290 | 420 | 176 |
|  |  |  | ALHF_11146.g6196 | 682 | 134 | 0 |
|  |  |  | ALHF_11147.g6197 | 569 | 193 | 0 |
|  |  |  | ALHF_11148.g6198 | 292 | 87 | 39 |
|  |  |  | ALHF_11149.g6199 | 292 | 87 | 39 |
|  |  | Ribosomal protein S17 | ALHF_10648.g5954 | 1533 | 155 | 122 |
|  |  | Ring finger domain | ALHF_06553.g4330 | 378 | 321 | 163 |
|  | RNA processing | KH domain | ALHF_03319.g2045 | 258 | 11 | 59 |
|  |  |  | ALHF_08212.g5178 | 85 | 0 | 2 |
|  |  |  | ALHF_10989.g6108 | 298 | 224 | 220 |
|  | Transcription | AAA domain | ALHF_02357.g1368 | 6832 | 6686 | 5240 |
|  | Translation | Translation | ALHF_07856.g5006 | 329 | 282 | 138 |
|  |  | eRF1 domain 2 | ALHF_03276.g2014 | 371 | 288 | 222 |
|  |  | FERM C-terminal PH-like domain | ALHF_08788.g5428 | 268 | 189 | 94 |
| Intra-cellular processes | Cell cycle, Apoptosis | Nuclear RNA-splicing-associated protein | ALHF_06595.g4349 | 347 | 42 | 13 |
|  |  | Rhodanese-like domain | ALHF_03333.g2057 | 342 | 287 | 278 |
|  | Cell motility | Gelsolin repeat | ALHF_03264.g2007 | 1298 | 560 | 0 |
|  |  | Leucine rich repeat | ALHF_03850.g2447 | 1344 | 232 | 133 |
|  |  | Myosin tail | ALHF_02771.g1633 | 66340 | 24388 | 8809 |
|  |  | Protein of unknown function (DUF445) | ALHF_05414.g3717 | 1732 | 238 | 124 |
|  |  | Transglutaminase-like superfamily | ALHF_09429.g5645 | 138 | 33 | 9 |
|  |  | Tropomyosin | ALHF_00247.g116 | 11157 | 825 | 88 |
|  |  |  | ALHF_00248.g117 | 10303 | 4077 | 1759 |
|  |  |  | ALHF_02518.g1468 | 22512 | 158 | 39 |
|  |  |  | ALHF_02519.g1469 | 53418 | 13756 | 3966 |
|  |  | Troponin | ALHF_01625.g903 | 13627 | 1060 | 55 |
|  |  |  | ALHF_01626.g904 | 30743 | 6038 | 1747 |
|  |  |  | ALHF_01627.g905 | 1026 | 48 | 0 |
|  |  |  | ALHF_01628.g906 | 2726 | 654 | 60 |
|  | Ion m/tr**^††^** | Ion m/tr | ALHF_09078.g5521 | 43 | 42 | 6 |
|  |  | Calcium-activated BK potassium channel alpha subunit | ALHF_04838.g3274 | 367 | 269 | 68 |
|  |  |  | ALHF_11826.g6647 | 493 | 228 | 43 |
|  |  |  | ALHF_11827.g6648 | 339 | 220 | 58 |
|  |  | CLN3 protein | ALHF_05802.g3947 | 359 | 190 | 160 |
|  |  |  | ALHF_11968.g6716 | 359 | 190 | 160 |
|  |  | Cyclic nucleotide-binding domain | ALHF_11165.g6209 | 100 | 26 | 23 |
|  |  | E1-E2 ATPase | ALHF_01209.g663 | 1257 | 304 | 91 |
|  |  |  | ALHF_04639.g3103 | 200678 | 71067 | 17744 |
|  |  |  | ALHF_06938.g4537 | 581 | 516 | 329 |
|  |  |  | ALHF_11225.g6243 | 502 | 430 | 126 |
|  |  | Ion channel | ALHF_09289.g5601 | 89 | 47 | 25 |
|  |  |  | ALHF_13115.g6797 | 31 | 17 | 13 |
|  |  |  | ALHF_13834.g6825 | 35 | 13 | 10 |
|  |  | Ion channel regulatory protein UNC-93 | ALHF_02245.g1298 | 4150 | 3083 | 1516 |
|  |  | Ion transport protein | ALHF_02830.g1683 | 514 | 193 | 114 |
|  |  |  | ALHF_08230.g5186 | 159 | 74 | 38 |
|  |  |  | ALHF_08388.g5258 | 71 | 57 | 17 |
|  |  | Ligand-gated ion channel | ALHF_00227.g109 | 1195 | 707 | 372 |
|  |  |  | ALHF_04008.g2576 | 467 | 153 | 118 |
|  |  |  | ALHF_04718.g3165 | 575 | 506 | 112 |
|  |  | Major Facilitator Superfamily | ALHF_00922.g513 | 231 | 194 | 46 |
|  |  |  | ALHF_01103.g611 | 218 | 166 | 71 |
|  |  |  | ALHF_01751.g981 | 680 | 251 | 54 |
|  |  |  | ALHF_02305.g1338 | 1791 | 1584 | 1305 |
|  |  |  | ALHF_02618.g1531 | 294 | 144 | 112 |
|  |  |  | ALHF_03601.g2248 | 941 | 153 | 76 |
|  |  |  | ALHF_03853.g2449 | 1075 | 985 | 206 |
|  |  |  | ALHF_04032.g2596 | 530 | 197 | 59 |
|  |  |  | ALHF_04598.g3070 | 494 | 233 | 119 |
|  |  |  | ALHF_05274.g3617 | 4287 | 3670 | 3394 |
|  |  |  | ALHF_05897.g3996 | 910 | 314 | 121 |
|  |  |  | ALHF_05902.g3999 | 770 | 344 | 74 |
|  |  | MFS_1 like family | ALHF_11797.g6629 | 270 | 192 | 53 |
|  |  | Multicopper oxidase | ALHF_05751.g3918 | 901 | 19 | 4 |
|  |  | MYND finger | ALHF_00601.g335 | 655 | 189 | 54 |
|  |  |  | ALHF_10183.g5817 | 655 | 189 | 54 |
|  |  | Neurotransmitter-gated ion-channel ligand binding domain | ALHF_02465.g1434 | 227 | 76 | 40 |
|  |  |  | ALHF_06514.g4308 | 254 | 109 | 68 |
|  |  | Organic Anion Transporter Polypeptide (OATP) family | ALHF_03646.g2286 | 1191 | 1185 | 1067 |
|  |  | Protein of unknown function (DUF2722) | ALHF_07433.g4795 | 94 | 26 | 37 |
|  |  | Salivary protein of 15kDa inhibits CD4+ T cell activation | ALHF_03948.g2532 | 1041 | 772 | 201 |
|  |  | SET domain | ALHF_00869.g479 | 686 | 300 | 74 |
|  |  |  | ALHF_01720.g964 | 524 | 174 | 116 |
|  |  |  | ALHF_03184.g1945 | 905 | 38 | 11 |
|  |  |  | ALHF_03723.g2344 | 314 | 30 | 2 |
|  |  |  | ALHF_03891.g2481 | 831 | 138 | 47 |
|  |  |  | ALHF_04768.g3213 | 1615 | 1505 | 686 |
|  |  |  | ALHF_06563.g4334 | 179 | 29 | 29 |
|  |  | SPFH domain / Band 7 family | ALHF_02541.g1482 | 276 | 227 | 191 |
|  |  | Sugar (and other) transporter | ALHF_01028.g568 | 218 | 187 | 81 |
|  |  |  | ALHF_02959.g1778 | 622 | 292 | 437 |
|  |  |  | ALHF_03418.g2118 | 465 | 430 | 151 |
|  |  |  | ALHF_04807.g3246 | 1198 | 1046 | 280 |
|  |  |  | ALHF_07044.g4596 | 303 | 270 | 51 |
|  |  | Voltage gated chloride channel | ALHF_04172.g2715 | 586 | 583 | 445 |
|  | Phospholipid m/tr | CRAL/TRIO domain | ALHF_01891.g1067 | 165 | 109 | 41 |
|  |  |  | ALHF_02966.g1782 | 312 | 268 | 111 |
|  |  |  | ALHF_03939.g2523 | 259 | 74 | 46 |
|  |  |  | ALHF_06707.g4410 | 175 | 54 | 27 |
|  |  |  | ALHF_06840.g4487 | 374 | 0 | 2 |
|  |  |  | ALHF_01427.g782 | 516 | 148 | 318 |
|  |  |  | ALHF_07369.g4759 | 46 | 34 | 14 |
|  |  | Glycerophosphoryl diester phosphodiesterase family | ALHF_01618.g898 | 7001 | 2987 | 508 |
|  |  |  | ALHF_02214.g1271 | 82 | 70 | 49 |
|  |  |  | ALHF_12223.g6756 | 82 | 70 | 49 |
|  |  | Phospholipase A2 | ALHF_08621.g5365 | 70 | 56 | 34 |
|  | Proteases | 2-enoyl-CoA Hydratase C-terminal region | ALHF_03878.g2468 | 822 | 0 | 0 |
|  |  | 5'-nucleotidase, C-terminal domain | ALHF_01615.g896 | 1005 | 776 | 872 |
|  |  |  | ALHF_02480.g1442 | 608 | 607 | 233 |
|  |  |  | ALHF_05374.g3692 | 2480 | 1780 | 987 |
|  |  | Alpha amylase, catalytic domain | ALHF_10633.g5951 | 18431 | 12798 | 5064 |
|  |  |  | ALHF_10956.g6083 | 18431 | 12798 | 5064 |
|  |  | Angiotensin-converting enzyme | ALHF_06928.g4532 | 211 | 4 | 2 |
|  |  | Calcineurin-like phosphoesterase | ALHF_00863.g476 | 1801 | 1108 | 500 |
|  |  |  | ALHF_02447.g1421 | 393 | 277 | 149 |
|  |  |  | ALHF_02448.g1422 | 1165 | 562 | 233 |
|  |  |  | ALHF_11364.g6335 | 312 | 0 | 0 |
|  |  | Chitin binding Peritrophin-A domain | ALHF_00899.g498 | 4981 | 443 | 46 |
|  |  | Cytosol aminopeptidase family, catalytic domain | ALHF_05334.g3663** | 5213 | 3226 | 2606 |
|  |  | Domain of unknown function (DUF3358) | ALHF_05593.g3829 | 875 | 576 | 309 |
|  |  |  | ALHF_06361.g4230 | 507 | 387 | 222 |
|  |  |  | ALHF_11113.g6173 | 288 | 0 | 12 |
|  |  | Eukaryotic aspartyl protease | ALHF_06529.g4317** | 71 | 37 | 21 |
|  |  | GON domain | ALHF_04951.g3366 | 813 | 724 | 265 |
|  |  | Insect cuticle protein | ALHF_08056.g5108 | 56 | 4 | 5 |
|  |  | Insulinase (Peptidase family M16) | ALHF_03227.g1979 | 40790 | 20287 | 11009 |
|  |  |  | ALHF_04836.g3272 | 25288 | 12393 | 5427 |
|  |  | Metallo-peptidase family M12B Reprolysin-like | ALHF_03402.g2108 | 333 | 144 | 55 |
|  |  | Metallopeptidase family M24 | ALHF_04237.g2764 | 573 | 450 | 170 |
|  |  | Mitochondrial fission regulator | ALHF_04728.g3174 | 1527 | 947 | 628 |
|  |  | Papain family cysteine protease | ALHF_04504.g2989 | 1817 | 1636 | 413 |
|  |  | PDZ domain (Also known as DHR or GLGF) | ALHF_01791.g1004 | 165 | 54 | 24 |
|  |  | Peptidase family M13 | ALHF_00121.g66 | 108 | 74 | 50 |
|  |  |  | ALHF_00761.g417** | 924 | 17 | 111 |
|  |  |  | ALHF_02207.g1267** | 1200 | 18 | 139 |
|  |  |  | ALHF_03210.g1966 | 546 | 222 | 88 |
|  |  |  | ALHF_03218.g1970** | 425 | 118 | 1 |
|  |  |  | ALHF_03290.g2021 | 284 | 163 | 74 |
|  |  |  | ALHF_07461.g4814 | 204 | 203 | 112 |
|  |  |  | ALHF_07511.g4836** | 276 | 106 | 63 |
|  |  |  | ALHF_11169.g6212 | 308 | 0 | 0 |
|  |  | Peptidase family M20/M25/M40 | ALHF_06480.g4292 | 266 | 122 | 90 |
|  |  | Peptidase family M28 | ALHF_01861.g1049** | 810 | 501 | 275 |
|  |  | Prokaryotic RING finger family 2 | ALHF_06864.g4501 | 46 | 33 | 35 |
|  |  | Rhomboid family | ALHF_07946.g5049 | 85 | 53 | 29 |
|  |  | Serpin (serine protease inhibitor) | ALHF_01182.g646** | 283 | 171 | 58 |
|  |  |  | ALHF_06478.g4290 | 299 | 257 | 96 |
|  |  |  | ALHF_07374.g4763** | 149 | 45 | 11 |
|  |  | Spondin_N | ALHF_11530.g6453 | 2184 | 1548 | 788 |
|  |  | Subtilase family | ALHF_00530.g295** | 509 | 100 | 108 |
|  |  | Transglutaminase family, C-terminal ig like domain | ALHF_04789.g3231 | 1190 | 1092 | 343 |
|  |  | Trypsin | ALHF_03472.g2155 | 2644 | 2543 | 1614 |
|  |  |  | ALHF_05672.g3873 | 143 | 67 | 37 |
|  |  |  | ALHF_08244.g5194 | 50 | 17 | 14 |
|  |  |  | ALHF_08259.g5198 | 48 | 7 | 4 |
|  |  |  | ALHF_10761.g5998 | 40 | 18 | 6 |
|  |  |  | ALHF_10834.g6035 | 217 | 116 | 36 |
|  |  |  | ALHF_10883.g6059 | 217 | 116 | 36 |
|  |  | Ubiquitin carboxyl-terminal hydrolase | ALHF_04382.g2883 | 5375 | 4710 | 4043 |
|  |  |  | ALHF_04436.g2930 | 202 | 87 | 35 |
|  |  |  | ALHF_04437.g2931 | 351 | 269 | 256 |
|  |  |  | ALHF_11571.g6479 | 202 | 87 | 35 |
|  |  |  | ALHF_11613.g6505 | 202 | 87 | 35 |
|  |  |  | ALHF_11614.g6506 | 351 | 269 | 256 |
|  |  | Ubiquitin carboxyl-terminal hydrolase, family 1 | ALHF_04698.g3154 | 849 | 452 | 779 |
|  |  |  | ALHF_11715.g6576 | 849 | 452 | 779 |
|  |  |  | ALHF_11716.g6578 | 849 | 452 | 779 |
|  |  | Zinc carboxypeptidase | ALHF_02473.g1438 | 216 | 12 | 30 |
|  |  |  | ALHF_02563.g1501 | 87 | 9 | 29 |
|  |  |  | ALHF_04057.g2616** | 411 | 297 | 52 |
|  |  |  | ALHF_04058.g2617 | 411 | 297 | 52 |
|  |  |  | ALHF_05871.g3981** | 456 | 393 | 196 |
|  |  |  | ALHF_06114.g4109 | 344 | 67 | 96 |
|  | Protein modification | Alcohol dehydrogenase GroES-like domain | ALHF_04916.g3340 | 6699 | 1912 | 1246 |
|  |  | DnaJ domain | ALHF_00820.g449 | 77 | 28 | 25 |
|  |  | Hsp20/alpha crystallin family | ALHF_02696.g1574 | 452 | 398 | 62 |
|  |  |  | ALHF_02697.g1575 | 2012 | 1530 | 1435 |
|  |  | Zinc-binding dehydrogenase | ALHF_04663.g3124 | 5904 | 5019 | 1464 |
|  | Transport | Transport | ALHF_01096.g606 | 159 | 81 | 60 |
|  |  | Adaptor complexes medium subunit family | ALHF_11038.g6132 | 670 | 320 | 155 |
|  |  | Ammonium Transporter Family | ALHF_03421.g2120 | 1210 | 1041 | 291 |
|  |  | Lipocalin-like domain | ALHF_03313.g2042 | 6 | 0 | 0 |
|  |  | Major intrinsic protein | ALHF_02488.g1448 | 1297 | 281 | 119 |
|  |  |  | ALHF_03651.g2291 | 2159 | 2125 | 871 |
|  |  | Mitochondrial carrier protein | ALHF_01907.g1077 | 268 | 212 | 56 |
|  |  |  | ALHF_02806.g1663 | 322 | 0 | 87 |
|  |  |  | ALHF_03742.g2359 | 120 | 0 | 0 |
|  |  |  | ALHF_04653.g3114 | 120 | 0 | 0 |
|  |  |  | ALHF_04658.g3119 | 740 | 716 | 302 |
|  |  |  | ALHF_05065.g3452 | 120017 | 62773 | 37768 |
|  |  |  | ALHF_05228.g3580 | 45105 | 6963 | 1204 |
|  |  | Synaphin protein | ALHF_04491.g2978 | 171 | 0 | 32 |
|  |  | Synaptobrevin | ALHF_03815.g2420 | 64 | 63 | 23 |
|  |  |  | ALHF_03817.g2422 | 62 | 28 | 46 |
|  |  |  | ALHF_03818.g2423 | 282 | 94 | 43 |
|  |  | TAP C-terminal domain | ALHF_09984.g5751 | 36 | 0 | 0 |
|  |  | Vacuolar sorting protein 9 (VPS9) domain | ALHF_04588.g3061 | 1360 | 1147 | 695 |
|  |  |  | ALHF_04589.g3062 | 1381 | 864 | 245 |
| Metabolism | Amino acids m/tr | Alanine racemase, N-terminal domain | ALHF_05396.g3705 | 1495 | 1101 | 791 |
|  |  | Arginase family | ALHF_00757.g414 | 963 | 157 | 175 |
|  |  | ATP:guanido phosphotransferase, C-terminal catalytic domain | ALHF_02200.g1264 | 25685 | 18750 | 11230 |
|  |  | Glutamine synthetase, catalytic domain | ALHF_00639.g356 | 1176 | 761 | 193 |
|  |  | Histone deacetylase domain | ALHF_11334.g6313 | 309 | 62 | 0 |
|  |  | Lyase | ALHF_05075.g3460 | 6103 | 3175 | 2131 |
|  |  |  | ALHF_05181.g3541 | 14679 | 7720 | 5523 |
|  | Carbohydrate m/tr | Carbohydrate m/tr | ALHF_01653.g924 | 2298 | 139 | 22 |
|  |  | Alpha amylase, catalytic domain | ALHF_01186.g650 | 1843 | 936 | 688 |
|  |  |  | ALHF_02580.g1510 | 13090 | 7550 | 4373 |
|  |  |  | ALHF_05361.g3682 | 4420 | 659 | 404 |
|  |  |  | ALHF_10902.g6067 | 4690 | 3430 | 1717 |
|  |  | Amylo-alpha-1,6-glucosidase | ALHF_00210.g104 | 4604 | 3896 | 1424 |
|  |  |  | ALHF_09960.g5743 | 3922 | 3776 | 2999 |
|  |  | Carbamoyl-phosphate synthase L chain, ATP binding domain | ALHF_11333.g6312 | 2698 | 896 | 1132 |
|  |  | Chitin binding Peritrophin-A domain | ALHF_00232.g112 | 268 | 226 | 109 |
|  |  |  | ALHF_00815.g446 | 1472 | 137 | 18 |
|  |  |  | ALHF_01311.g716 | 120 | 38 | 5 |
|  |  |  | ALHF_01581.g873 | 508 | 279 | 215 |
|  |  |  | ALHF_02754.g1623 | 854 | 119 | 4 |
|  |  |  | ALHF_04064.g2623 | 1334 | 312 | 482 |
|  |  |  | ALHF_04461.g2953 | 908 | 137 | 27 |
|  |  |  | ALHF_10203.g5825 | 341 | 88 | 0 |
|  |  |  | ALHF_13141.g6800 | 28 | 21 | 13 |
|  |  | Glycosyl hydrolase family 20, catalytic domain | ALHF_04498.g2984 | 3954 | 3764 | 1259 |
|  |  |  | ALHF_11242.g6250 | 157 | 0 | 0 |
|  |  | Glycosyl hydrolases family 15 | ALHF_11410.g6363 | 1260 | 1234 | 461 |
|  |  | Glycosyl hydrolases family 18 | ALHF_00537.g301 | 1556 | 500 | 578 |
|  |  |  | ALHF_03417.g2117 | 898 | 127 | 14 |
|  |  |  | ALHF_04634.g3099 | 685 | 566 | 398 |
|  |  |  | ALHF_05040.g3430 | 777 | 133 | 310 |
|  |  |  | ALHF_05511.g3780 | 1849 | 184 | 65 |
|  |  |  | ALHF_09467.g5654 | 970 | 113 | 146 |
|  |  | Glycosyl hydrolases family 31 | ALHF_04205.g2738 | 21879 | 4438 | 3156 |
|  |  | Glycosyl hydrolases family 35 | ALHF_05236.g3587 | 14347 | 11467 | 4017 |
|  |  | Inositol monophosphatase family | ALHF_05470.g3753 | 639 | 554 | 309 |
|  |  |  | ALHF_07119.g4640 | 151 | 104 | 82 |
|  |  | Melibiase | ALHF_05697.g3891 | 804 | 539 | 181 |
|  |  | O-Glycosyl hydrolase family 30 | ALHF_06944.g4540 | 218 | 200 | 30 |
|  | Coenzyme m/tr | Acyl-CoA dehydrogenase, C-terminal domain | ALHF_02197.g1263 | 5939 | 5766 | 3676 |
|  |  |  | ALHF_04575.g3052 | 995 | 988 | 765 |
|  |  | ATP-grasp domain | ALHF_05230.g3581 | 18273 | 8174 | 3781 |
|  |  | CoA binding domain | ALHF_03845.g2442 | 8665 | 4849 | 2338 |
|  |  | Pyridoxamine 5'-phosphate oxidase | ALHF_00313.g155 | 596 | 568 | 506 |
|  |  |  | ALHF_09951.g5737 | 596 | 568 | 506 |
|  |  | Uroporphyrinogen-III synthase HemD | ALHF_01245.g681 | 1487 | 688 | 299 |
|  | E- transfer | Acyl-CoA oxidase | ALHF_07656.g4903 | 273 | 105 | 44 |
|  |  | Cytochrome b5-like Heme/Steroid binding domain | ALHF_03764.g2382 | 228 | 158 | 44 |
|  |  | Cytochrome c oxidase subunit Va | ALHF_05256.g3601 | 14890 | 8179 | 3919 |
|  |  | Cytochrome C1 family | ALHF_05098.g3476 | 22391 | 9584 | 4582 |
|  |  | Molybdopterin-binding domain of aldehyde dehydrogenase | ALHF_01294.g708 | 12367 | 7607 | 4865 |
|  |  |  | ALHF_04462.g2954 | 6459 | 5648 | 2404 |
|  | Energy | Aconitase family (aconitate hydratase) | ALHF_03992.g2565 | 61323 | 46607 | 18323 |
|  |  | ATP synthase | ALHF_05104.g3480 | 25588 | 13448 | 6004 |
|  |  | ATP synthase delta (OSCP) subunit | ALHF_05261.g3605 | 17417 | 7157 | 3854 |
|  |  | ATP synthase, Delta/Epsilon chain, beta-sandwich domain | ALHF_01931.g1090 | 10966 | 5088 | 2784 |
|  |  | Citrate synthase | ALHF_02275.g1322 | 7994 | 6066 | 3993 |
|  |  |  | ALHF_03396.g2102 | 37831 | 11413 | 4872 |
|  |  | Cytochrome c oxidase subunit IV | ALHF_05205.g3562 | 20747 | 13826 | 6310 |
|  |  | Enolase, C-terminal TIM barrel domain | ALHF_05078.g3461 | 26091 | 25473 | 16517 |
|  |  | Glyceraldehyde 3-phosphate dehydrogenase, C-terminal domain | ALHF_05242.g3592 | 62094 | 39624 | 27514 |
|  |  | Low-density lipoprotein receptor repeat class B | ALHF_09535.g5677 | 223 | 78 | 37 |
|  |  | NAD-dependent glycerol-3-phosphate dehydrogenase N-terminus | ALHF_04089.g2643 | 9723 | 2799 | 0 |
|  |  |  | ALHF_11471.g6406 | 5208 | 2641 | 189 |
|  |  |  | ALHF_11733.g6586 | 5208 | 2641 | 189 |
|  |  | Phosphoenolpyruvate carboxykinase | ALHF_03356.g2072 | 1164 | 740 | 361 |
|  |  |  | ALHF_05166.g3527 | 22456 | 13590 | 5311 |
|  |  | Phosphofructokinase | ALHF_03481.g2159 | 8843 | 6892 | 2949 |
|  |  | Phosphoglycerate kinase | ALHF_02451.g1425 | 16037 | 12467 | 6116 |
|  |  | Pyruvate kinase, barrel domain | ALHF_05097.g3475 | 38594 | 28411 | 13862 |
|  |  | SIS domain | ALHF_05159.g3521 | 4597 | 1778 | 436 |
|  |  | Triosephosphate isomerase | ALHF_05217.g3571 | 4894 | 3520 | 3628 |
|  |  | Ubiquinol cytochrome reductase transmembrane region | ALHF_03676.g2306 | 9067 | 3354 | 2525 |
|  |  |  | ALHF_10969.g6092 | 10 | 0 | 0 |
|  |  |  | ALHF_11313.g6297 | 6876 | 4805 | 1886 |
|  |  | Ubiquinol-cytochrome C reductase complex 14kD subunit | ALHF_05150.g3517 | 8024 | 3786 | 1640 |
|  | Lipid m/tr | Low-density lipoprotein receptor domain class A | ALHF_11812.g6638 | 193 | 178 | 45 |
|  |  |  | ALHF_11816.g6642 | 193 | 178 | 45 |
|  |  | Low-density lipoprotein receptor repeat class B | ALHF_08587.g5348 | 180 | 127 | 70 |
|  |  |  | ALHF_09255.g5589 | 144 | 99 | 55 |
|  |  | Thioesterase superfamily | ALHF_05641.g3856 | 247 | 152 | 125 |
|  | Nitrogen m/tr | Cysteine dioxygenase type I | ALHF_05522.g3786 | 577 | 444 | 160 |
|  |  | homogentisate 1,2-dioxygenase | ALHF_03932.g2516 | 3044 | 663 | 212 |
|  | Nucleotide m/tr | Glutamine amidotransferases class-II | ALHF_03927.g2512 | 12213 | 2036 | 1227 |
|  |  | GTP cyclohydrolase I | ALHF_10143.g5801 | 500 | 348 | 69 |
|  |  | Inosine-uridine preferring nucleoside hydrolase | ALHF_07208.g4686 | 186 | 151 | 127 |
|  |  | N-terminal domain of ribose phosphate pyrophosphokinase | ALHF_02932.g1755 | 3871 | 3558 | 1501 |
|  |  | Nicotinate phosphoribosyltransferase (NAPRTase) family | ALHF_04019.g2584 | 796 | 664 | 440 |
|  |  | Phosphorylase superfamily | ALHF_03888.g2478 | 8849 | 3372 | 1905 |
|  |  | RNA pseudouridylate synthase | ALHF_00353.g178 | 694 | 606 | 207 |
|  |  | SAICAR synthetase | ALHF_01554.g856 | 14360 | 7563 | 4782 |
|  | Other enzymes | Other enzymes | ALHF_03904.g2494 | 1944 | 1478 | 678 |
|  |  |  | ALHF_03336.g2059 | 1348 | 128 | 13 |
|  |  |  | ALHF_03844.g2441 | 97 | 87 | 84 |
|  |  | 3'5'-cyclic nucleotide phosphodiesterase | ALHF_01977.g1115 | 399 | 211 | 137 |
|  |  |  | ALHF_07436.g4798 | 739 | 292 | 87 |
|  |  |  | ALHF_09987.g5752 | 267 | 0 | 0 |
|  |  | 6-phosphofructo-2-kinase | ALHF_05053.g3442 | 856 | 90 | 73 |
|  |  |  | ALHF_05054.g3443 | 856 | 90 | 73 |
|  |  | Actin | ALHF_04131.g2677 | 49486 | 359 | 747 |
|  |  |  | ALHF_04132.g2678 | 104486 | 6153 | 1603 |
|  |  |  | ALHF_04133.g2679 | 23170 | 17939 | 7802 |
|  |  |  | ALHF_05088.g3469 | 62138 | 6498 | 804 |
|  |  |  | ALHF_11302.g6290 | 23588 | 195 | 367 |
|  |  |  | ALHF_11303.g6291 | 10880 | 6541 | 2540 |
|  |  | Adenosine/AMP deaminase | ALHF_00267.g128 | 426 | 377 | 18 |
|  |  |  | ALHF_03974.g2551 | 1504 | 781 | 661 |
|  |  | AICARFT/IMPCHase bienzyme | ALHF_05162.g3524 | 14733 | 8359 | 7376 |
|  |  | Aldose 1-epimerase | ALHF_05429.g3726 | 1019 | 983 | 380 |
|  |  | Alpha/beta hydrolase family | ALHF_07255.g4704 | 184 | 139 | 50 |
|  |  | AMP-binding enzyme | ALHF_02432.g1410 | 3884 | 1327 | 1421 |
|  |  |  | ALHF_03983.g2556 | 575 | 173 | 23 |
|  |  |  | ALHF_07655.g4902 | 155 | 39 | 22 |
|  |  |  | ALHF_11670.g6551 | 188 | 58 | 59 |
|  |  | Asparagine synthase | ALHF_02059.g1167 | 718 | 265 | 43 |
|  |  | Carboxylesterase family | ALHF_00771.g422** | 173 | 11 | 38 |
|  |  |  | ALHF_03407.g2111** | 1582 | 1478 | 957 |
|  |  |  | ALHF_05354.g3678 | 3605 | 3345 | 1105 |
|  |  |  | ALHF_05628.g3847** | 637 | 322 | 227 |
|  |  |  | ALHF_06969.g4553 | 332 | 215 | 113 |
|  |  |  | ALHF_07911.g5033 | 117 | 56 | 21 |
|  |  | Chitin binding Peritrophin-A domain | ALHF_04061.g2620 | 10883 | 866 | 27 |
|  |  |  | ALHF_04643.g3106 | 167 | 17 | 0 |
|  |  |  | ALHF_04969.g3373 | 969 | 81 | 3 |
|  |  | Cytochrome c oxidase subunit Vb | ALHF_05083.g3465 | 11402 | 6300 | 2998 |
|  |  | Diacylglycerol kinase accessory domain | ALHF_09025.g5505 | 288 | 135 | 81 |
|  |  | Diacylglycerol kinase catalytic domain | ALHF_03550.g2208 | 690 | 342 | 227 |
|  |  | Epoxide hydrolase N terminus | ALHF_02789.g1649 | 1053 | 1015 | 321 |
|  |  |  | ALHF_03576.g2229 | 2157 | 1186 | 788 |
|  |  | Eukaryotic protein of unknown function (DUF829) | ALHF_08826.g5439 | 111 | 78 | 19 |
|  |  | Eukaryotic-type carbonic anhydrase | ALHF_07437.g4799 | 103 | 99 | 68 |
|  |  | FAD dependent oxidoreductase | ALHF_03915.g2502 | 1159 | 83 | 147 |
|  |  | Family of unknown function (DUF706) | ALHF_03164.g1934 | 8243 | 5685 | 4472 |
|  |  | FGGY family of carbohydrate kinases, N-terminal domain | ALHF_05865.g3977 | 686 | 600 | 455 |
|  |  | Gamma-glutamyltranspeptidase | ALHF_00515.g284 | 654 | 582 | 233 |
|  |  | GDSL-like Lipase/Acylhydrolase | ALHF_00017.g13 | 91 | 15 | 14 |
|  |  | haloacid dehalogenase-like hydrolase | ALHF_01674.g938 | 3193 | 2742 | 1905 |
|  |  |  | ALHF_10747.g5991 | 3193 | 2742 | 1905 |
|  |  | Haloacid dehalogenase-like hydrolase | ALHF_11680.g6555 | 552 | 482 | 406 |
|  |  | Histidine phosphatase superfamily (branch 1) | ALHF_00338.g172 | 13997 | 9103 | 6402 |
|  |  |  | ALHF_02151.g1230 | 232 | 128 | 27 |
|  |  | Histidine phosphatase superfamily (branch 2) | ALHF_00026.g19 | 58 | 28 | 14 |
|  |  | Hsp70 protein | ALHF_02010.g1139 | 308 | 298 | 215 |
|  |  |  | ALHF_04050.g2610 | 440 | 228 | 100 |
|  |  | Hydroxymethylglutaryl-coenzyme A synthase C terminal | ALHF_05452.g3740 | 1799 | 856 | 1158 |
|  |  | Lecithin:cholesterol acyltransferase | ALHF_11440.g6382 | 712 | 232 | 239 |
|  |  | LNS2 (Lipin/Ned1/Smp2) | ALHF_04145.g2690 | 7092 | 3093 | 2999 |
|  |  | Low-density lipoprotein receptor repeat class B | ALHF_04953.g3368 | 113 | 0 | 0 |
|  |  | Major royal jelly protein | ALHF_02988.g1801 | 729 | 2 | 2 |
|  |  |  | ALHF_05690.g3885 | 532 | 322 | 128 |
|  |  |  | ALHF_02145.g1227 | 354 | 30 | 6 |
|  |  |  | ALHF_04469.g2959 | 700 | 105 | 53 |
|  |  | NHL repeat | ALHF_03688.g2317 | 13068 | 5496 | 2352 |
|  |  | Partial alpha/beta-hydrolase lipase region | ALHF_03701.g2328 | 531 | 283 | 159 |
|  |  | pfkB family carbohydrate kinase | ALHF_01925.g1088 | 1051 | 808 | 765 |
|  |  |  | ALHF_00333.g168 | 204 | 143 | 184 |
|  |  | PGAP1-like protein | ALHF_05226.g3578 | 17430 | 7587 | 5409 |
|  |  | Prolyl oligopeptidase family | ALHF_00110.g62 | 52 | 22 | 19 |
|  |  | Protein of unknown function (DUF3236) | ALHF_02967.g1783 | 895 | 665 | 318 |
|  |  | Protein tyrosine kinase | ALHF_11144.g6194** | 153 | 30 | 19 |
|  |  |  | ALHF_11245.g6252** | 75 | 59 | 37 |
|  |  |  | ALHF_09312.g5609** | 309 | 213 | 115 |
|  |  | Respiratory-chain NADH dehydrogenase, 30 Kd subunit | ALHF_05186.g3546 | 6843 | 3961 | 2346 |
|  |  | Respiratory-chain NADH dehydrogenase, 49 Kd subunit | ALHF_05227.g3579 | 12705 | 8366 | 4318 |
|  |  | SMP-30/Gluconolaconase/LRE-like region | ALHF_03369.g2082 | 1882 | 1723 | 363 |
|  |  | Succinate dehydrogenase/Fumarate reductase transmembrane subunit | ALHF_05192.g3552 | 4836 | 2501 | 1293 |
|  |  | Sulfatase | ALHF_03367.g2080 | 290 | 279 | 89 |
|  |  | Trehalose-phosphatase | ALHF_00476.g257 | 4228 | 2144 | 1285 |
|  | Polysaccharide m/tr | Carbohydrate phosphorylase | ALHF_05160.g3522 | 20902 | 17223 | 9671 |
|  |  | UDP-glucoronosyl and UDP-glucosyl transferase | ALHF_01552.g854 | 369 | 240 | 135 |
|  |  |  | ALHF_03127.g1906 | 900 | 670 | 492 |
|  |  |  | ALHF_04857.g3291 | 1313 | 496 | 344 |
|  | Redox | 2Fe-2S iron-sulfur cluster binding domain | ALHF_05201.g3560 | 9430 | 8390 | 3428 |
|  |  | 4Fe-4S binding domain | ALHF_02233.g1288 | 5354 | 3762 | 2299 |
|  |  | AhpC/TSA family | ALHF_05086.g3467 | 9643 | 5488 | 5088 |
|  |  | Aldo/keto reductase family | ALHF_00277.g132 | 3389 | 1971 | 2195 |
|  |  |  | ALHF_00278.g133 | 4194 | 2996 | 2216 |
|  |  |  | ALHF_04352.g2865 | 1143 | 910 | 912 |
|  |  | Animal haem peroxidase | ALHF_04662.g3123 | 1328 | 545 | 119 |
|  |  |  | ALHF_05952.g4029 | 1718 | 1609 | 496 |
|  |  |  | ALHF_07134.g4646 | 589 | 7 | 5 |
|  |  |  | ALHF_07487.g4829 | 362 | 10 | 5 |
|  |  | Biopterin-dependent aromatic amino acid hydroxylase | ALHF_03152.g1923 | 2322 | 839 | 198 |
|  |  | Copper/zinc superoxide dismutase (SODC) | ALHF_04809.g3248 | 338 | 284 | 83 |
|  |  | Cytochrome P450 | ALHF_01339.g731** | 267 | 168 | 50 |
|  |  |  | ALHF_01822.g1025** | 302 | 129 | 157 |
|  |  |  | ALHF_02791.g1651** | 437 | 334 | 223 |
|  |  |  | ALHF_03006.g1816** | 1313 | 586 | 567 |
|  |  |  | ALHF_03063.g1860** | 382 | 322 | 216 |
|  |  |  | ALHF_03088.g1882** | 493 | 385 | 231 |
|  |  |  | ALHF_03849.g2446** | 2181 | 2110 | 674 |
|  |  |  | ALHF_04444.g2938** | 634 | 178 | 254 |
|  |  |  | ALHF_04445.g2939** | 826 | 433 | 310 |
|  |  |  | ALHF_04553.g3033** | 492 | 404 | 296 |
|  |  |  | ALHF_04665.g3125** | 446 | 3 | 46 |
|  |  |  | ALHF_04730.g3176** | 234 | 110 | 116 |
|  |  |  | ALHF_04736.g3182** | 278 | 218 | 53 |
|  |  |  | ALHF_05136.g3505** | 9632 | 5497 | 3221 |
|  |  |  | ALHF_05265.g3608** | 2144 | 14 | 329 |
|  |  |  | ALHF_07553.g4857** | 167 | 26 | 42 |
|  |  |  | ALHF_07623.g4891** | 109 | 74 | 41 |
|  |  |  | ALHF_08221.g5182** | 51 | 22 | 39 |
|  |  | Glyoxalase/Bleomycin resistance protein/Dioxygenase superfamily | ALHF_05310.g3644 | 3416 | 807 | 179 |
|  |  | Heme oxygenase | ALHF_00886.g489 | 796 | 455 | 376 |
|  |  | Glutathione S-transferase, C-terminal domain | ALHF_03731.g2351** | 1814 | 868 | 539 |
|  |  | Glutathione S-transferase, N-terminal domain | ALHF_03123.g1902 | 35640 | 2508 | 598 |
|  |  |  | ALHF_04476.g2964** | 5777 | 5380 | 1816 |
|  |  |  | ALHF_04477.g2965** | 8872 | 7837 | 3873 |
|  |  |  | ALHF_04900.g3328** | 669 | 388 | 308 |
|  |  |  | ALHF_03145.g1917** | 240 | 185 | 67 |
|  |  | Isocitrate/isopropylmalate dehydrogenase | ALHF_02312.g1343 | 12502 | 7126 | 2987 |
|  |  |  | ALHF_05245.g3593 | 12095 | 6695 | 2952 |
|  |  | lactate/malate dehydrogenase, NAD binding domain | ALHF_05238.g3589 | 28623 | 15877 | 8597 |
|  |  | Malic enzyme, NAD binding domain | ALHF_04528.g3011 | 191 | 62 | 29 |
|  |  |  | ALHF_04529.g3012 | 1358 | 1257 | 1083 |
|  |  | Molybdopterin oxidoreductase | ALHF_02044.g1158 | 19826 | 14086 | 6892 |
|  |  | PAP2 superfamily | ALHF_07944.g5047 | 127 | 21 | 8 |
|  |  | Redoxin | ALHF_00473.g254 | 4297 | 2831 | 1140 |
|  |  | Respiratory-chain NADH dehydrogenase 24 Kd subunit | ALHF_01507.g827 | 4580 | 2777 | 1519 |
|  |  | SH3-binding, glutamic acid-rich protein | ALHF_03946.g2530 | 144 | 0 | 96 |
|  |  | Thioredoxin | ALHF_03860.g2454 | 3837 | 1253 | 216 |
|  |  |  | ALHF_03861.g2455 | 1896 | 848 | 313 |
|  |  |  | ALHF_04448.g2942 | 860 | 391 | 220 |
|  |  | Thioredoxin-like domain | ALHF_03862.g2456 | 6148 | 2652 | 498 |
|  | Secondary metabolism | Secondary metabolism | ALHF_02742.g1612 | 517 | 39 | 0 |
|  |  |  | ALHF_10958.g6085 | 690 | 191 | 2 |
|  |  |  | ALHF_10959.g6086 | 973 | 112 | 74 |
|  |  | A-macroglobulin complement component | ALHF_10494.g5906 | 129 | 67 | 12 |
|  |  |  | ALHF_05903.g4000 | 584 | 192 | 199 |
|  |  | Cartilage oligomeric matrix protein | ALHF_01950.g1101 | 312 | 167 | 116 |
|  |  |  | ALHF_10814.g6024 | 312 | 167 | 116 |
|  |  | Eukaryotic cobalamin-binding protein | ALHF_02635.g1543 | 277 | 160 | 82 |
|  |  | Galactoside-binding lectin | ALHF_03944.g2528 | 708 | 45 | 16 |
|  |  |  | ALHF_07168.g4663 | 232 | 192 | 127 |
|  |  | Laminin G domain | ALHF_04344.g2860 | 1798 | 603 | 142 |
|  |  |  | ALHF_05604.g3833 | 351 | 217 | 39 |
|  |  |  | ALHF_11117.g6176 | 1368 | 825 | 440 |
|  |  |  | ALHF_11118.g6177 | 351 | 217 | 39 |
|  |  |  | ALHF_11119.g6178 | 1368 | 825 | 440 |
|  |  | LMBR1-like membrane protein | ALHF_02420.g1404 | 1460 | 241 | 108 |
|  |  |  | ALHF_10957.g6084 | 384 | 187 | 47 |
|  |  | MAM domain | ALHF_05901.g3998 | 988 | 888 | 297 |
|  |  | Pentaxin family | ALHF_02157.g1233 | 330 | 58 | 7 |
|  |  | Ras family | ALHF_03061.g1858 | 119 | 71 | 28 |
|  |  | Taurine catabolism dioxygenase TauD, TfdA family inosine | ALHF_02458.g1430 | 314 | 257 | 160 |
|  |  | Thrombospondin C-terminal region | ALHF_01949.g1100 | 817 | 374 | 185 |
|  | Transferases | 2-oxoacid dehydrogenases acyltransferase (catalytic domain) | ALHF_03097.g1888 | 15494 | 8120 | 3864 |
|  |  |  | ALHF_05147.g3515 | 17341 | 10604 | 4834 |
|  |  | Acetyltransferase (GNAT) family | ALHF_06713.g4415 | 179 | 151 | 128 |
|  |  | Aminotransferase class I and II | ALHF_02444.g1420 | 2409 | 1411 | 228 |
|  |  |  | ALHF_03635.g2275 | 6321 | 5448 | 4161 |
|  |  | Aminotransferase class IV | ALHF_03433.g2126 | 4717 | 1318 | 480 |
|  |  | Aminotransferase class-III | ALHF_04644.g3107 | 1942 | 1329 | 638 |
|  |  | Aminotransferase class-V | ALHF_05248.g3596 | 8630 | 8112 | 4342 |
|  |  | Chitin synthase | ALHF_04825.g3262 | 3218 | 238 | 11 |
|  |  | Choline/Carnitine o-acyltransferase | ALHF_01598.g884 | 1511 | 1409 | 1272 |
|  |  |  | ALHF_10528.g5922 | 1511 | 1409 | 1272 |
|  |  | CobB/CobQ-like glutamine amidotransferase domain | ALHF_05277.g3619 | 13565 | 6678 | 5347 |
|  |  | Cyclic nucleotide-binding domain | ALHF_03490.g2167 | 409 | 373 | 280 |
|  |  | FR47-like protein | ALHF_06363.g4231 | 222 | 96 | 50 |
|  |  |  | ALHF_09510.g5667 | 70 | 28 | 11 |
|  |  | Glycine cleavage system P-protein | ALHF_03961.g2544 | 11663 | 5931 | 2260 |
|  |  | Insect cuticle protein | ALHF_02884.g1721 | 463 | 99 | 15 |
|  |  | Methyltransferase domain | ALHF_03190.g1950 | 3401 | 2320 | 1156 |
|  |  | Neurotransmitter-gated ion-channel transmembrane region | ALHF_13736.g6821 | 51 | 30 | 47 |
|  |  | Patatin-like phospholipase | ALHF_04896.g3324 | 4551 | 2985 | 2797 |
|  |  | Phosphoribosylglycinamide synthetase, ATP-grasp (A) domain | ALHF_05327.g3659 | 16332 | 11242 | 6142 |
|  |  | Protein tyrosine kinase | ALHF_03649.g2289** | 228 | 219 | 120 |
|  |  | Pyridoxal-dependent decarboxylase conserved domain | ALHF_05668.g3872 | 825 | 368 | 204 |
|  |  |  | ALHF_08999.g5500 | 89 | 61 | 16 |
|  |  |  | ALHF_13676.g6818 | 47 | 38 | 15 |
|  |  | Ricin-type beta-trefoil lectin domain | ALHF_08475.g5301 | 102 | 95 | 66 |
|  |  | RIH domain | ALHF_04262.g2788 | 27782 | 9215 | 3127 |
|  |  | Serine hydroxymethyltransferase | ALHF_05234.g3585 | 13881 | 12580 | 7568 |
|  |  | UTP--glucose-1-phosphate uridylyltransferase | ALHF_00405.g212 | 1509 | 820 | 731 |
| Regulation | DNA-binding | C2H2-type zinc finger | ALHF_07757.g4954 | 121 | 28 | 12 |
|  |  |  | ALHF_08660.g5381 | 75 | 35 | 17 |
|  |  | CUT domain | ALHF_08770.g5421 | 174 | 117 | 56 |
|  |  | Eukaryotic translation initiation factor 3 subunit 8 N-terminus | ALHF_11439.g6381 | 15307 | 13028 | 14277 |
|  |  | Fork head domain | ALHF_08819.g5437 | 101 | 93 | 44 |
|  |  | Helix-loop-helix DNA-binding domain | ALHF_05537.g3797 | 445 | 400 | 329 |
|  |  |  | ALHF_07653.g4901 | 90 | 53 | 42 |
|  |  | HMG (high mobility group) box | ALHF_00384.g200 | 845 | 391 | 142 |
|  |  |  | ALHF_04895.g3323 | 1074 | 1045 | 917 |
|  |  |  | ALHF_07248.g4699 | 290 | 139 | 77 |
|  |  | Homeobox domain | ALHF_09298.g5604 | 39 | 24 | 12 |
|  |  | Homeobox KN domain | ALHF_00760.g416 | 176 | 123 | 57 |
|  |  | Ligand-gated ion channel | ALHF_00783.g429 | 321 | 73 | 63 |
|  |  | LIM domain | ALHF_02271.g1320 | 1948 | 1579 | 509 |
|  |  |  | ALHF_04092.g2644 | 10530 | 4537 | 325 |
|  |  | Pou domain - N-terminal to homeobox domain | ALHF_06649.g4378 | 171 | 165 | 43 |
|  |  | Rel homology domain (RHD) | ALHF_03698.g2325 | 823 | 643 | 274 |
|  |  | Zinc-finger double domain | ALHF_03082.g1876 | 245 | 106 | 31 |
|  |  |  | ALHF_11156.g6203 | 450 | 442 | 416 |
|  | Kinases/phosphatases | Kinases/phosphatases | ALHF_03461.g2146 | 1157 | 226 | 84 |
|  |  |  | ALHF_10746.g5990 | 403 | 357 | 86 |
|  |  | Dual specificity phosphatase, catalytic domain | ALHF_11825.g6646 | 728 | 15 | 282 |
|  |  | Ecdysteroid kinase | ALHF_00383.g199 | 407 | 77 | 85 |
|  |  |  | ALHF_00653.g364 | 117 | 52 | 16 |
|  |  |  | ALHF_01017.g561 | 264 | 228 | 131 |
|  |  |  | ALHF_02818.g1673 | 804 | 414 | 128 |
|  |  |  | ALHF_02948.g1769 | 570 | 564 | 150 |
|  |  |  | ALHF_05383.g3699 | 2089 | 477 | 259 |
|  |  |  | ALHF_06088.g4096 | 281 | 235 | 98 |
|  |  |  | ALHF_07356.g4751 | 164 | 140 | 98 |
|  |  |  | ALHF_11384.g6351 | 391 | 389 | 249 |
|  |  | Guanylate kinase | ALHF_11325.g6304 | 203 | 90 | 118 |
|  |  | Immunoglobulin I-set domain | ALHF_00307.g152 | 2113 | 378 | 144 |
|  |  |  | ALHF_02477.g1440 | 24200 | 10921 | 3512 |
|  |  |  | ALHF_03144.g1916 | 543 | 275 | 127 |
|  |  |  | ALHF_04487.g2975 | 48516 | 18935 | 5574 |
|  |  |  | ALHF_05185.g3545 | 108439 | 10280 | 1627 |
|  |  |  | ALHF_09013.g5502 | 78 | 40 | 11 |
|  |  |  | ALHF_11408.g6361 | 180 | 153 | 20 |
|  |  | Myosin head (motor domain) | ALHF_05296.g3631 | 8767 | 7039 | 3154 |
|  |  | PKC-activated protein phosphatase-1 inhibitor | ALHF_02356.g1367 | 126 | 114 | 44 |
|  |  | Protein kinase domain | ALHF_00685.g381** | 784 | 189 | 75 |
|  |  |  | ALHF_00727.g395** | 245 | 238 | 113 |
|  |  |  | ALHF_00823.g452** | 1228 | 722 | 297 |
|  |  |  | ALHF_01595.g882** | 1150 | 673 | 312 |
|  |  |  | ALHF_01832.g1033** | 284 | 198 | 77 |
|  |  |  | ALHF_02546.g1487** | 166 | 75 | 36 |
|  |  |  | ALHF_02885.g1722** | 298 | 222 | 53 |
|  |  |  | ALHF_03341.g2063 | 1397 | 1031 | 304 |
|  |  |  | ALHF_03462.g2147** | 1425 | 329 | 38 |
|  |  |  | ALHF_04095.g2646** | 2814 | 1546 | 640 |
|  |  |  | ALHF_04187.g2728 | 2589 | 0 | 2441 |
|  |  |  | ALHF_04500.g2986** | 1271 | 1230 | 430 |
|  |  |  | ALHF_04611.g3081 | 847 | 149 | 0 |
|  |  |  | ALHF_04614.g3084 | 751 | 184 | 0 |
|  |  |  | ALHF_04616.g3086 | 144 | 95 | 93 |
|  |  |  | ALHF_06710.g4412 | 2437 | 0 | 1356 |
|  |  |  | ALHF_08078.g5122** | 125 | 91 | 85 |
|  |  |  | ALHF_11277.g6269** | 287 | 131 | 80 |
|  |  |  | ALHF_11278.g6270 | 665 | 0 | 0 |
|  |  |  | ALHF_11442.g6384** | 478 | 439 | 269 |
|  |  |  | ALHF_11684.g6556 | 538 | 289 | 0 |
|  |  |  | ALHF_11596.g6493 | 106 | 0 | 51 |
|  |  | Protein phosphatase 2C | ALHF_06769.g4446 | 563 | 282 | 176 |
|  |  | Protein tyrosine kinase | ALHF_05773.g3933** | 202 | 163 | 118 |
|  |  |  | ALHF_07173.g4665** | 746 | 581 | 209 |
|  |  |  | ALHF_10712.g5974** | 57 | 43 | 24 |
|  |  |  | ALHF_11829.g6650** | 405 | 326 | 236 |
|  |  | Protein-tyrosine phosphatase | ALHF_03863.g2457** | 130 | 102 | 40 |
|  |  |  | ALHF_09146.g5552 | 152 | 101 | 109 |
|  |  |  | ALHF_11768.g6612** | 114 | 0 | 0 |
|  |  |  | ALHF_11769.g6613 | 114 | 0 | 0 |
|  |  | UL92 family | ALHF_08305.g5220 | 224 | 61 | 36 |
|  | Other regulatory function | Mob1/phocein family | ALHF_04834.g3271 | 422 | 323 | 83 |
|  | Receptor activity | Insect pheromone-binding family, A10/OS-D | ALHF_00416.g219 | 5978 | 4035 | 2367 |
|  |  | Lysyl oxidase | ALHF_06487.g4295 | 287 | 164 | 32 |
|  |  | UL45 protein | ALHF_00880.g486 | 424 | 400 | 264 |
|  | RNA binding, m/tr | ATP-dependent protease La (LON) domain | ALHF_00188.g95 | 665 | 594 | 235 |
|  |  | RNA recognition motif. (a.k.a. RRM, RBD, or RNP domain) | ALHF_04127.g2674 | 400 | 363 | 170 |
|  |  |  | ALHF_10458.g5891 | 137 | 12 | 0 |
|  |  | SUZ domain | ALHF_11351.g6326 | 959 | 796 | 509 |
|  | Signal transduction | Signal transduction | ALHF_11090.g6160 | 210 | 66 | 20 |
|  |  |  | ALHF_11091.g6161 | 210 | 66 | 20 |
|  |  |  | ALHF_11508.g6438 | 210 | 66 | 20 |
|  |  |  | ALHF_03300.g2029 | 4328 | 3851 | 2243 |
|  |  |  | ALHF_10904.g6068 | 258 | 82 | 223 |
|  |  |  | ALHF_11661.g6543 | 981 | 952 | 713 |
|  |  | 7 transmembrane receptor (rhodopsin-like GPCR family) | ALHF_01760.g986** | 235 | 157 | 70 |
|  |  |  | ALHF_02706.g1581** | 18922 | 18497 | 11723 |
|  |  |  | ALHF_04422.g2918** | 847 | 725 | 303 |
|  |  |  | ALHF_06811.g4468** | 235 | 197 | 178 |
|  |  |  | ALHF_07519.g4838** | 59 | 42 | 18 |
|  |  | 7TM-HD extracellular | ALHF_02400.g1393** | 286 | 272 | 183 |
|  |  | Adenylate and Guanylate cyclase catalytic domain | ALHF_01050.g580** | 589 | 443 | 200 |
|  |  |  | ALHF_07748.g4948** | 140 | 121 | 45 |
|  |  | ATP synthase alpha/beta family, nucleotide-binding domain | ALHF_01778.g998 | 139134 | 61184 | 33560 |
|  |  | C2 domain | ALHF_06727.g4423 | 272 | 116 | 47 |
|  |  |  | ALHF_07183.g4672 | 5513 | 4993 | 4617 |
|  |  | Complement Clr-like EGF-like | ALHF_08913.g5469 | 78 | 12 | 9 |
|  |  | EGF domain | ALHF_05090.g3471 | 3550 | 2381 | 488 |
|  |  | Frizzled/Smoothened family membrane region | ALHF_09621.g5698 | 109 | 77 | 32 |
|  |  | Fumarylacetoacetate (FAA) hydrolase family | ALHF_04648.g3108 | 1443 | 638 | 264 |
|  |  | Fungal domain of unknown function (DUF1750) | ALHF_02430.g1409 | 127 | 72 | 50 |
|  |  | Guanylate kinase | ALHF_11413.g6366 | 21 | 5 | 16 |
|  |  | Heme NO binding associated | ALHF_00553.g312 | 448 | 144 | 73 |
|  |  | Hemocyanin, copper containing domain | ALHF_04934.g3357 | 2151 | 5 | 12 |
|  |  | Insect cuticle protein | ALHF_03881.g2471 | 2937 | 72 | 3 |
|  |  |  | ALHF_08022.g5091 | 44 | 0 | 0 |
|  |  | La domain | ALHF_04264.g2790 | 317 | 0 | 0 |
|  |  | Leucine-zipper of ternary complex factor MIP1 | ALHF_03446.g2134 | 57 | 49 | 34 |
|  |  | Ligand-binding domain of nuclear hormone receptor | ALHF_07047.g4597 | 174 | 125 | 58 |
|  |  | LIM domain | ALHF_04874.g3305 | 538 | 478 | 145 |
|  |  |  | ALHF_11668.g6549 | 22321 | 332 | 163 |
|  |  | Low-density lipoprotein receptor domain class A | ALHF_01477.g811 | 420 | 143 | 44 |
|  |  | MIZ/SP-RING zinc finger | ALHF_07577.g4869 | 724 | 619 | 340 |
|  |  | N-terminal domain of unknown function (DUF4140) | ALHF_06081.g4093 | 342 | 299 | 188 |
|  |  | Neurotransmitter-gated ion-channel ligand binding domain | ALHF_02495.g1452 | 324 | 142 | 33 |
|  |  | PBP/GOBP family | ALHF_05128.g3497 | 1588 | 1291 | 596 |
|  |  |  | ALHF_05611.g3837 | 416 | 277 | 113 |
|  |  |  | ALHF_10169.g5811 | 122 | 73 | 45 |
|  |  |  | ALHF_04854.g3288 | 1529 | 838 | 380 |
|  |  | PDZ domain (Also known as DHR or GLGF) | ALHF_02132.g1220 | 7722 | 2333 | 662 |
|  |  |  | ALHF_02133.g1222 | 2669 | 1002 | 229 |
|  |  |  | ALHF_04830.g3267 | 7560 | 1918 | 196 |
|  |  |  | ALHF_05273.g3616 | 1846 | 446 | 37 |
|  |  |  | ALHF_05275.g3618 | 1264 | 1112 | 259 |
|  |  |  | ALHF_05476.g3758 | 2363 | 2338 | 1599 |
|  |  |  | ALHF_11205.g6229 | 531 | 68 | 8 |
|  |  |  | ALHF_11576.g6483 | 634 | 420 | 119 |
|  |  |  | ALHF_11578.g6485 | 97 | 88 | 56 |
|  |  | Phosphotyrosine interaction domain (PTB/PID) | ALHF_01855.g1044 | 179 | 132 | 87 |
|  |  |  | ALHF_04566.g3046 | 336 | 66 | 265 |
|  |  | Phosphotyrosine-binding domain | ALHF_02476.g1439 | 713 | 479 | 274 |
|  |  |  | ALHF_03968.g2549 | 6368 | 6061 | 2446 |
|  |  | Raf-like Ras-binding domain | ALHF_09388.g5633 | 86 | 34 | 19 |
|  |  | RhoGAP domain | ALHF_10240.g5836 | 290 | 77 | 75 |
|  |  | RhoGEF domain | ALHF_00186.g93 | 213 | 136 | 105 |
|  |  |  | ALHF_06971.g4554 | 340 | 164 | 112 |
|  |  | Serpentine type 7TM GPCR chemoreceptor Srw | ALHF_01902.g1074** | 251 | 183 | 79 |
|  |  | Serpin (serine protease inhibitor) | ALHF_02807.g1664 | 7066 | 392 | 9 |
|  |  | SET domain | ALHF_04979.g3382 | 583 | 347 | 226 |
|  |  | SH3 domain | ALHF_04669.g3129 | 542 | 89 | 108 |
|  |  |  | ALHF_11705.g6568 | 960 | 563 | 2 |
|  |  |  | ALHF_11706.g6569 | 1089 | 114 | 882 |
|  |  |  | ALHF_11708.g6571 | 209 | 201 | 5 |
|  |  |  | ALHF_11659.g6541 | 398 | 288 | 101 |
|  |  | Stathmin family | ALHF_04225.g2756 | 212 | 106 | 44 |
|  |  |  | ALHF_04226.g2757 | 120 | 18 | 22 |
|  |  |  | ALHF_11128.g6185 | 109 | 57 | 2 |
|  |  | Variant SH3 domain | ALHF_08997.g5499 | 140 | 113 | 83 |
| Other | Unknown function | Arrestin (or S-antigen), N-terminal domain | ALHF_05063.g3451 | 10560 | 10107 | 5267 |
|  |  |  | ALHF_07976.g5066 | 171 | 143 | 48 |
|  |  | Beige/BEACH domain | ALHF_03517.g2181 | 1943 | 429 | 742 |
|  |  |  | ALHF_03518.g2183 | 1943 | 429 | 742 |
|  |  | BSD domain | ALHF_00449.g237 | 395 | 158 | 73 |
|  |  | Hemocyanin, ig-like domain | ALHF_04590.g3063 | 794 | 89 | 5 |
|  |  |  | ALHF_04773.g3217 | 6016 | 36 | 21 |
|  |  |  | ALHF_05487.g3766 | 683 | 39 | 2 |
|  |  |  | ALHF_06765.g4443 | 119 | 0 | 0 |
|  |  | Inward rectifier potassium channel | ALHF_04542.g3025 | 256 | 20 | 53 |
|  |  | ML domain | ALHF_06345.g4224 | 41 | 31 | 3 |
|  |  | Sodium/calcium exchanger protein | ALHF_03036.g1842 | 550 | 452 | 206 |
| Not annotated | not annotated |  | ALHF_03661.g2297 | 195 | 139 | 70 |
|  |  |  | ALHF_00388.g203 | 373 | 301 | 161 |
|  |  |  | ALHF_00432.g227 | 301 | 218 | 0 |
|  |  |  | ALHF_00748.g408 | 2259 | 1146 | 592 |
|  |  |  | ALHF_00756.g413 | 103 | 74 | 18 |
|  |  |  | ALHF_01005.g553 | 111 | 49 | 8 |
|  |  |  | ALHF_01185.g649 | 45 | 37 | 9 |
|  |  |  | ALHF_01436.g788 | 1562 | 796 | 381 |
|  |  |  | ALHF_01470.g805 | 278 | 59 | 27 |
|  |  |  | ALHF_01570.g865 | 215 | 142 | 51 |
|  |  |  | ALHF_01577.g871 | 1817 | 1511 | 644 |
|  |  |  | ALHF_01671.g936 | 860 | 846 | 792 |
|  |  |  | ALHF_01690.g950 | 209 | 29 | 16 |
|  |  |  | ALHF_02000.g1130 | 882 | 225 | 3 |
|  |  |  | ALHF_02016.g1142 | 61 | 61 | 31 |
|  |  |  | ALHF_02067.g1174 | 202 | 199 | 69 |
|  |  |  | ALHF_02341.g1357 | 599 | 314 | 79 |
|  |  |  | ALHF_03133.g1907 | 128 | 39 | 37 |
|  |  |  | ALHF_03303.g2032 | 12422 | 1982 | 866 |
|  |  |  | ALHF_03304.g2033 | 651 | 310 | 34 |
|  |  |  | ALHF_03770.g2385 | 2053 | 1405 | 635 |
|  |  |  | ALHF_03773.g2388 | 410 | 88 | 19 |
|  |  |  | ALHF_03896.g2486 | 1911 | 1754 | 410 |
|  |  |  | ALHF_04072.g2629 | 2515 | 1498 | 673 |
|  |  |  | ALHF_04081.g2636 | 51 | 5 | 4 |
|  |  |  | ALHF_04082.g2637 | 51 | 5 | 4 |
|  |  |  | ALHF_04115.g2663 | 1433 | 833 | 424 |
|  |  |  | ALHF_04126.g2673 | 65 | 18 | 6 |
|  |  |  | ALHF_04220.g2751 | 331 | 198 | 168 |
|  |  |  | ALHF_04221.g2752 | 266 | 263 | 67 |
|  |  |  | ALHF_04258.g2785 | 846 | 739 | 350 |
|  |  |  | ALHF_04385.g2886 | 939 | 475 | 300 |
|  |  |  | ALHF_04472.g2962 | 427 | 411 | 76 |
|  |  |  | ALHF_04585.g3060 | 1015 | 304 | 141 |
|  |  |  | ALHF_04591.g3064 | 1665 | 712 | 513 |
|  |  |  | ALHF_04751.g3196 | 4723 | 2986 | 1273 |
|  |  |  | ALHF_04855.g3289 | 2457 | 284 | 13 |
|  |  |  | ALHF_04990.g3389 | 514 | 342 | 183 |
|  |  |  | ALHF_05011.g3407 | 172 | 0 | 24 |
|  |  |  | ALHF_05012.g3408 | 172 | 0 | 24 |
|  |  |  | ALHF_05013.g3409 | 1168 | 0 | 350 |
|  |  |  | ALHF_05014.g3410 | 1168 | 0 | 350 |
|  |  |  | ALHF_05049.g3438 | 401 | 293 | 157 |
|  |  |  | ALHF_05068.g3455 | 1404 | 1170 | 1083 |
|  |  |  | ALHF_05173.g3533 | 611 | 548 | 237 |
|  |  |  | ALHF_05174.g3534 | 611 | 548 | 237 |
|  |  |  | ALHF_05300.g3635 | 1331 | 1306 | 280 |
|  |  |  | ALHF_05520.g3785 | 1266 | 836 | 249 |
|  |  |  | ALHF_05741.g3912 | 194 | 34 | 15 |
|  |  |  | ALHF_05851.g3972 | 1420 | 190 | 114 |
|  |  |  | ALHF_05913.g4009 | 438 | 100 | 37 |
|  |  |  | ALHF_06503.g4303 | 44 | 40 | 23 |
|  |  |  | ALHF_07137.g4648 | 322 | 0 | 1 |
|  |  |  | ALHF_07178.g4668 | 275 | 86 | 92 |
|  |  |  | ALHF_07456.g4813 | 149 | 88 | 71 |
|  |  |  | ALHF_07727.g4941 | 100 | 51 | 63 |
|  |  |  | ALHF_07790.g4974 | 158 | 46 | 20 |
|  |  |  | ALHF_07865.g5009 | 91 | 68 | 7 |
|  |  |  | ALHF_08122.g5136 | 44 | 24 | 3 |
|  |  |  | ALHF_08276.g5209 | 232 | 160 | 74 |
|  |  |  | ALHF_08520.g5319 | 71 | 8 | 8 |
|  |  |  | ALHF_08653.g5376 | 851 | 624 | 694 |
|  |  |  | ALHF_08700.g5396 | 46 | 36 | 30 |
|  |  |  | ALHF_09600.g5693 | 17 | 2 | 4 |
|  |  |  | ALHF_10003.g5760 | 5362 | 23 | 6 |
|  |  |  | ALHF_10029.g5769 | 9 | 0 | 0 |
|  |  |  | ALHF_10057.g5785 | 2457 | 284 | 13 |
|  |  |  | ALHF_10239.g5835 | 166 | 100 | 74 |
|  |  |  | ALHF_10416.g5880 | 459 | 40 | 10 |
|  |  |  | ALHF_10517.g5914 | 230 | 130 | 64 |
|  |  |  | ALHF_10563.g5932 | 860 | 846 | 792 |
|  |  |  | ALHF_10765.g6001 | 330 | 233 | 141 |
|  |  |  | ALHF_10766.g6002 | 64 | 5 | 1 |
|  |  |  | ALHF_10801.g6018 | 968 | 370 | 217 |
|  |  |  | ALHF_10802.g6019 | 2543 | 1077 | 361 |
|  |  |  | ALHF_10803.g6020 | 139 | 4 | 1 |
|  |  |  | ALHF_10830.g6032 | 599 | 314 | 79 |
|  |  |  | ALHF_11198.g6227 | 559 | 420 | 208 |
|  |  |  | ALHF_11362.g6333 | 364 | 46 | 34 |
|  |  |  | ALHF_11363.g6334 | 364 | 46 | 34 |
|  |  |  | ALHF_11436.g6378 | 1291 | 901 | 561 |
|  |  |  | ALHF_11517.g6442 | 121 | 0 | 0 |
|  |  |  | ALHF_11522.g6446 | 1628 | 916 | 491 |
|  |  |  | ALHF_11629.g6519 | 493 | 104 | 0 |
|  |  |  | ALHF_11631.g6521 | 529 | 478 | 329 |
|  |  |  | ALHF_11771.g6614 | 2457 | 284 | 13 |
|  |  |  | ALHF_12063.g6737 | 84 | 70 | 60 |
|  |  |  | ALHF_12498.g6765 | 82 | 60 | 32 |
|  |  |  | ALHF_00817.g448 | 125 | 2 | 0 |
|  |  |  | ALHF_07071.g4614 | 194 | 134 | 66 |
|  |  |  | ALHF_09367.g5625 | 42 | 32 | 8 |
|  |  |  | ALHF_05070.g3457 | 23760 | 9357 | 805 |
|  |  |  | ALHF_02181.g1250 | 941 | 797 | 173 |
|  |  |  | ALHF_09999.g5758 | 941 | 797 | 173 |
|  |  |  | ALHF_04998.g3397 | 2555 | 1884 | 1141 |
|  |  |  | ALHF_05835.g3966 | 738 | 594 | 299 |
|  |  |  | ALHF_06691.g4404 | 311 | 158 | 159 |
|  |  |  | ALHF_11729.g6582 | 622 | 547 | 192 |
|  |  |  | ALHF_01167.g638 | 105 | 45 | 87 |
|  |  |  | ALHF_01828.g1029 | 186 | 180 | 122 |
|  |  |  | ALHF_02736.g1608 | 679 | 340 | 224 |
|  |  |  | ALHF_06722.g4420 | 278 | 168 | 232 |
|  |  |  | ALHF_11250.g6255 | 299 | 148 | 33 |
|  |  |  | ALHF_03174.g1939 | 418 | 283 | 73 |
|  |  |  | ALHF_04176.g2719 | 1559 | 468 | 444 |
|  |  |  | ALHF_04177.g2720 | 1669 | 1551 | 603 |
|  |  |  | ALHF_06075.g4088 | 279 | 257 | 113 |
|  |  |  | ALHF_11457.g6395 | 586 | 471 | 533 |
|  |  |  | ALHF_02712.g1586 | 2042 | 1592 | 678 |
|  |  |  | ALHF_11564.g6472 | 685 | 490 | 199 |
|  |  |  | ALHF_11824.g6645 | 685 | 490 | 199 |
|  |  |  | ALHF_00900.g499 | 209 | 140 | 63 |
|  |  |  | ALHF_01541.g846 | 650 | 224 | 84 |
|  |  |  | ALHF_01651.g923 | 125 | 73 | 41 |
|  |  |  | ALHF_01862.g1050 | 315 | 281 | 128 |
|  |  |  | ALHF_02033.g1152 | 109 | 13 | 6 |
|  |  |  | ALHF_02167.g1240 | 251 | 161 | 34 |
|  |  |  | ALHF_02223.g1280 | 60495 | 10830 | 101 |
|  |  |  | ALHF_02768.g1632 | 182 | 104 | 12 |
|  |  |  | ALHF_03597.g2246 | 5163 | 101 | 5 |
|  |  |  | ALHF_03603.g2250 | 637 | 395 | 263 |
|  |  |  | ALHF_03615.g2259 | 126 | 11 | 2 |
|  |  |  | ALHF_03794.g2400 | 664 | 78 | 8 |
|  |  |  | ALHF_04056.g2615 | 658 | 82 | 22 |
|  |  |  | ALHF_04071.g2628 | 158 | 86 | 24 |
|  |  |  | ALHF_04174.g2717 | 411 | 83 | 21 |
|  |  |  | ALHF_04175.g2718 | 555 | 99 | 39 |
|  |  |  | ALHF_04509.g2995 | 338 | 0 | 146 |
|  |  |  | ALHF_05056.g3445 | 275 | 116 | 4 |
|  |  |  | ALHF_05223.g3576 | 7917 | 4785 | 1927 |
|  |  |  | ALHF_05325.g3657 | 1502 | 721 | 313 |
|  |  |  | ALHF_05535.g3796 | 680 | 523 | 290 |
|  |  |  | ALHF_05585.g3825 | 29 | 5 | 7 |
|  |  |  | ALHF_05587.g3827 | 172 | 90 | 2 |
|  |  |  | ALHF_05604.g3834 | 9 | 1 | 0 |
|  |  |  | ALHF_07194.g4676 | 143 | 15 | 66 |
|  |  |  | ALHF_08346.g5243 | 380 | 219 | 284 |
|  |  |  | ALHF_09102.g5530 | 58 | 28 | 12 |
|  |  |  | ALHF_09171.g5560 | 79 | 17 | 7 |
|  |  |  | ALHF_09408.g5639 | 45 | 6 | 5 |
|  |  |  | ALHF_09437.g5648 | 69 | 55 | 44 |
|  |  |  | ALHF_09488.g5660 | 42 | 20 | 12 |
|  |  |  | ALHF_09917.g5726 | 264 | 37 | 14 |
|  |  |  | ALHF_10587.g5936 | 69 | 66 | 22 |
|  |  |  | ALHF_10716.g5978 | 88 | 69 | 36 |
|  |  |  | ALHF_11117.g6175 | 9 | 1 | 0 |
|  |  |  | ALHF_11120.g6179 | 9 | 1 | 0 |
|  |  |  | ALHF_11177.g6216 | 449 | 304 | 3 |
|  |  |  | ALHF_11180.g6217 | 565 | 5 | 3 |
|  |  |  | ALHF_11181.g6218 | 879 | 5 | 3 |
|  |  |  | ALHF_11272.g6266 | 721 | 262 | 103 |
|  |  |  | ALHF_11672.g6553 | 69 | 0 | 0 |
|  |  |  | ALHF_14067.g6833 | 26 | 15 | 6 |
|  |  |  | ALHF_07055.g4602 | 342 | 127 | 80 |
|  |  |  | ALHF_00317.g159 | 983 | 90 | 69 |
|  |  |  | ALHF_02588.g1514 | 230 | 122 | 79 |
|  |  |  | ALHF_05284.g3624 | 1931 | 1671 | 1262 |
|  |  |  | ALHF_02992.g1803 | 1116 | 980 | 621 |
|  |  |  | ALHF_08334.g5237 | 420 | 334 | 222 |
|  |  |  | ALHF_00408.g213 | 17412 | 10363 | 4725 |
|  |  |  | ALHF_10238.g5834 | 220 | 89 | 0 |
|  |  |  | ALHF_04320.g2841 | 724 | 654 | 235 |
|  |  |  | ALHF_04321.g2842 | 724 | 654 | 235 |
|  |  |  | ALHF_05341.g3667 | 1025 | 70 | 3 |
|  |  |  | ALHF_01957.g1105 | 61 | 54 | 37 |
|  |  |  | ALHF_00875.g484 | 186 | 96 | 69 |
|  |  |  | ALHF_02734.g1606 | 319 | 221 | 174 |
|  |  |  | ALHF_03536.g2197 | 418 | 356 | 143 |
|  |  |  | ALHF_02873.g1716 | 239 | 144 | 80 |
|  |  |  | ALHF_05015.g3411 | 163 | 0 | 20 |
|  |  |  | ALHF_05017.g3413 | 841 | 0 | 238 |
|  |  |  | ALHF_05018.g3414 | 879 | 868 | 485 |
|  |  |  | ALHF_07412.g4786 | 384 | 267 | 339 |
|  |  |  | ALHF_02550.g1491 | 9579 | 9305 | 5098 |
|  |  |  | ALHF_00475.g256 | 1698 | 1627 | 1455 |
|  |  |  | ALHF_01146.g628 | 299 | 118 | 100 |
|  |  |  | ALHF_04305.g2827 | 1839 | 1044 | 409 |
|  |  |  | ALHF_06280.g4186 | 410 | 364 | 134 |
|  |  |  | ALHF_11162.g6206 | 85 | 36 | 0 |
|  |  |  | ALHF_11171.g6214 | 207 | 0 | 0 |
|  |  |  | ALHF_01657.g928 | 368 | 265 | 201 |
|  |  |  | ALHF_05106.g3482 | 4380 | 2149 | 1373 |
|  |  |  | ALHF_01196.g656 | 98 | 65 | 63 |
|  |  |  | ALHF_08185.g5167 | 143 | 48 | 35 |
|  |  |  | ALHF_03913.g2500 | 294 | 153 | 164 |
|  |  |  | ALHF_04078.g2634 | 37234 | 18201 | 6057 |
|  |  |  | ALHF_14203.g6836 | 59 | 9 | 2 |
|  |  |  | ALHF_04774.g3218 | 760 | 123 | 51 |
|  |  |  | ALHF_05214.g3569 | 2446 | 1663 | 836 |
|  |  |  | ALHF_05252.g3599 | 4045 | 1750 | 1001 |
|  |  |  | ALHF_02985.g1798 | 299 | 79 | 1 |
|  |  |  | ALHF_08576.g5345 | 194 | 135 | 57 |
|  |  |  | ALHF_08439.g5285 | 187 | 81 | 58 |
|  |  |  | ALHF_11188.g6222 | 563 | 284 | 106 |
|  |  |  | ALHF_04300.g2822 | 2495 | 2222 | 1958 |
|  |  |  | ALHF_10408.g5878 | 144 | 48 | 19 |
|  |  |  | ALHF_11316.g6299 | 265 | 52 | 128 |
|  |  |  | ALHF_04416.g2913 | 7546 | 6642 | 2767 |
|  |  |  | ALHF_04417.g2914 | 2930 | 2771 | 1227 |
|  |  |  | ALHF_11603.g6501 | 423 | 351 | 107 |
|  |  |  | ALHF_07408.g4783 | 94 | 3 | 0 |
|  |  |  | ALHF_03110.g1897 | 1809 | 1130 | 507 |
|  |  |  | ALHF_03878.g2467 | 8 | 0 | 0 |
|  |  |  | ALHF_02281.g1324 | 778 | 267 | 109 |
|  |  |  | ALHF_07012.g4575 | 122 | 76 | 63 |
|  |  |  | ALHF_03401.g2107 | 191 | 131 | 69 |
|  |  |  | ALHF_04821.g3258 | 944 | 773 | 228 |
|  |  |  | ALHF_02764.g1630 | 4952 | 2530 | 111 |
|  |  |  | ALHF_03858.g2452 | 579 | 250 | 63 |
|  |  |  | ALHF_02834.g1687 | 965 | 200 | 36 |
|  |  |  | ALHF_07455.g4812 | 396 | 24 | 5 |
|  |  |  | ALHF_00457.g242 | 3228 | 1938 | 1007 |
|  |  |  | ALHF_06171.g4128 | 486 | 279 | 110 |
|  |  |  | ALHF_10483.g5901 | 486 | 279 | 110 |
|  |  |  | ALHF_05262.g3606 | 18451 | 17871 | 12301 |
|  |  |  | ALHF_03613.g2257 | 265 | 180 | 80 |
|  |  |  | ALHF_10502.g5909 | 84 | 81 | 41 |
|  |  |  | ALHF_12183.g6749 | 84 | 81 | 41 |
|  |  |  | ALHF_01611.g893 | 1852 | 916 | 190 |
|  |  |  | ALHF_01970.g1111 | 5507 | 3501 | 1229 |
|  |  |  | ALHF_05347.g3672 | 2172 | 203 | 77 |
|  |  |  | ALHF_03180.g1942 | 214 | 115 | 81 |
|  |  |  | ALHF_04899.g3327 | 5205 | 3413 | 1134 |
|  |  |  | ALHF_06547.g4326 | 420 | 357 | 76 |
|  |  |  | ALHF_01003.g551 | 303 | 231 | 127 |
|  |  |  | ALHF_09836.g5715 | 69 | 28 | 15 |
|  |  |  | ALHF_11745.g6595 | 116 | 0 | 10 |
|  |  |  | ALHF_08085.g5124 | 154 | 70 | 62 |
|  |  |  | ALHF_11498.g6429 | 388 | 0 | 48 |
|  |  |  | ALHF_00006.g5 | 94 | 29 | 24 |
|  |  |  | ALHF_07838.g4997 | 294 | 137 | 61 |
|  |  |  | ALHF_10892.g6063 | 194 | 143 | 58 |
|  |  |  | ALHF_11860.g6669 | 194 | 143 | 58 |
|  |  |  | ALHF_03094.g1885 | 1544 | 1399 | 441 |
|  |  |  | ALHF_05219.g3573 | 3207 | 3180 | 1804 |
|  |  |  | ALHF_05111.g3489 | 1003 | 950 | 555 |
|  |  |  | ALHF_04965.g3371 | 6841 | 216 | 10 |
|  |  |  | ALHF_05779.g3936 | 451 | 377 | 227 |
|  |  |  | ALHF_00832.g457 | 337 | 277 | 140 |
|  |  |  | ALHF_11523.g6447 | 358 | 75 | 81 |
|  |  |  | ALHF_11524.g6448 | 358 | 75 | 81 |
|  |  |  | ALHF_05089.g3470 | 3298 | 1601 | 1016 |
|  |  |  | ALHF_00873.g482 | 120 | 106 | 50 |
|  |  |  | ALHF_01249.g683 | 1781 | 1629 | 595 |
|  |  |  | ALHF_01682.g944 | 236 | 123 | 41 |
|  |  |  | ALHF_02968.g1784 | 48 | 37 | 10 |
|  |  |  | ALHF_02969.g1785 | 48 | 37 | 10 |
|  |  |  | ALHF_03678.g2308 | 446 | 259 | 54 |
|  |  |  | ALHF_05473.g3756 | 549 | 21 | 0 |
|  |  |  | ALHF_08170.g5162 | 178 | 4 | 81 |
|  |  |  | ALHF_09795.g5711 | 43 | 7 | 18 |
|  |  |  | ALHF_03746.g2364 | 2278 | 872 | 1008 |
|  |  |  | ALHF_10316.g5856 | 92 | 67 | 40 |
|  |  |  | ALHF_06676.g4395 | 75 | 33 | 37 |
|  |  |  | ALHF_11655.g6537 | 2406 | 363 | 197 |
|  |  |  | ALHF_04601.g3073 | 3584 | 0 | 0 |
|  |  |  | ALHF_04827.g3264 | 897 | 603 | 351 |
|  |  |  | ALHF_05350.g3675 | 1334 | 897 | 198 |
|  |  |  | ALHF_06329.g4216 | 1716 | 1035 | 432 |
|  |  |  | ALHF_10511.g5913 | 94 | 79 | 9 |
|  |  |  | ALHF_11621.g6512 | 4837 | 2626 | 412 |
|  |  |  | ALHF_11622.g6513 | 8439 | 6657 | 2202 |
|  |  |  | ALHF_04535.g3018 | 3121 | 1224 | 477 |
|  |  |  | ALHF_04733.g3179 | 12738 | 2472 | 345 |
|  |  |  | ALHF_04860.g3293 | 22450 | 11878 | 7619 |
|  |  |  | ALHF_08068.g5115 | 190 | 132 | 84 |
|  |  |  | ALHF_00341.g174 | 858 | 35 | 2 |
|  |  |  | ALHF_00501.g274 | 612 | 51 | 3 |
|  |  |  | ALHF_00591.g332 | 1845 | 204 | 6 |
|  |  |  | ALHF_01267.g691 | 213 | 58 | 3 |
|  |  |  | ALHF_01589.g880 | 260 | 132 | 19 |
|  |  |  | ALHF_02709.g1584 | 108 | 4 | 1 |
|  |  |  | ALHF_03064.g1861 | 257 | 3 | 1 |
|  |  |  | ALHF_03302.g2031 | 470 | 10 | 1 |
|  |  |  | ALHF_03631.g2272 | 984 | 470 | 240 |
|  |  |  | ALHF_04277.g2802 | 2290 | 1988 | 690 |
|  |  |  | ALHF_04642.g3105 | 3460 | 204 | 7 |
|  |  |  | ALHF_05199.g3558 | 11969 | 1987 | 11 |
|  |  |  | ALHF_05239.g3590 | 9505 | 965 | 13 |
|  |  |  | ALHF_05442.g3735 | 628 | 57 | 0 |
|  |  |  | ALHF_05633.g3851 | 232 | 30 | 2 |
|  |  |  | ALHF_06359.g4229 | 80 | 1 | 0 |
|  |  |  | ALHF_07156.g4656 | 240 | 38 | 68 |
|  |  |  | ALHF_11036.g6130 | 171 | 0 | 4 |
|  |  |  | ALHF_11301.g6289 | 77 | 13 | 7 |
|  |  |  | ALHF_09329.g5616 | 60 | 5 | 16 |
|  |  |  | ALHF_05580.g3820 | 222 | 91 | 47 |
|  |  |  | ALHF_02247.g1300 | 21124 | 10978 | 4510 |
|  |  |  | ALHF_05342.g3668 | 595 | 394 | 69 |
|  |  |  | ALHF_00908.g504 | 578 | 144 | 87 |
|  |  |  | ALHF_10475.g5896 | 578 | 144 | 87 |
|  |  |  | ALHF_04009.g2577 | 525 | 208 | 232 |
|  |  |  | ALHF_11019.g6124 | 153 | 28 | 0 |
|  |  |  | ALHF_01351.g737 | 2336 | 893 | 301 |
|  |  |  | ALHF_00587.g331 | 185 | 96 | 10 |
|  |  |  | ALHF_09792.g5709 | 125 | 67 | 26 |
|  |  |  | ALHF_04333.g2853 | 1276 | 982 | 509 |
|  |  |  | ALHF_08902.g5464 | 28 | 21 | 16 |
|  |  |  | ALHF_00330.g167 | 1096 | 1008 | 472 |
|  |  |  | ALHF_07216.g4689 | 74 | 43 | 27 |
|  |  |  | ALHF_06119.g4110 | 374 | 278 | 181 |
|  |  |  | ALHF_07781.g4968 | 160 | 142 | 60 |
|  |  |  | ALHF_02263.g1315 | 12554 | 6304 | 3343 |
|  |  |  | ALHF_11750.g6599 | 173 | 156 | 60 |
|  |  |  | ALHF_00644.g358 | 181 | 119 | 62 |
|  |  |  | ALHF_10745.g5989 | 97 | 63 | 23 |
|  |  |  | ALHF_11424.g6374 | 97 | 63 | 23 |
|  |  |  | ALHF_03025.g1832 | 20686 | 9557 | 5049 |
|  |  |  | ALHF_05197.g3556 | 5471 | 2660 | 2125 |
|  |  |  | ALHF_11366.g6337 | 18959 | 15131 | 6802 |
|  |  |  | ALHF_14312.g6838 | 13 | 1 | 1 |
|  |  |  | ALHF_03608.g2254 | 7357 | 6656 | 3936 |
|  |  |  | ALHF_05206.g3563 | 5400 | 1024 | 311 |
|  |  |  | ALHF_04070.g2627 | 712 | 52 | 3 |
|  |  |  | ALHF_07394.g4776 | 97 | 67 | 42 |
|  |  |  | ALHF_11579.g6486 | 16 | 0 | 7 |
|  |  |  | ALHF_00347.g176 | 875 | 353 | 393 |
|  |  |  | ALHF_00534.g299 | 1031 | 534 | 541 |
|  |  |  | ALHF_04506.g2991 | 320 | 65 | 13 |
|  |  |  | ALHF_05101.g3478 | 2200 | 1850 | 785 |
|  |  |  | ALHF_05195.g3554 | 3783 | 2077 | 1098 |
|  |  |  | ALHF_02419.g1403 | 3489 | 1830 | 1083 |
|  |  |  | ALHF_02725.g1599 | 5518 | 3261 | 1540 |
|  |  |  | ALHF_03311.g2040 | 377 | 210 | 143 |
|  |  |  | ALHF_00378.g196 | 76 | 69 | 60 |
|  |  |  | ALHF_03368.g2081 | 926 | 2 | 1 |
|  |  |  | ALHF_08662.g5382 | 71 | 5 | 4 |
|  |  |  | ALHF_11136.g6189 | 287 | 107 | 30 |
|  |  |  | ALHF_11137.g6190 | 413 | 255 | 51 |
|  |  |  | ALHF_06194.g4141 | 261 | 160 | 45 |
|  |  |  | ALHF_11817.g6643 | 302 | 173 | 13 |
|  |  |  | ALHF_02890.g1725 | 911 | 424 | 215 |
|  |  |  | ALHF_03664.g2300 | 591 | 535 | 357 |
|  |  |  | ALHF_11186.g6220 | 314 | 285 | 94 |
|  |  |  | ALHF_02741.g1611 | 615 | 339 | 339 |
|  |  |  | ALHF_10806.g6022 | 100 | 0 | 16 |
|  |  |  | ALHF_05905.g4002 | 306 | 271 | 144 |
|  |  |  | ALHF_04026.g2591 | 856 | 165 | 173 |
|  |  |  | ALHF_11525.g6449 | 500 | 293 | 218 |
|  |  |  | ALHF_00288.g141 | 183 | 70 | 78 |
|  |  |  | ALHF_01166.g637 | 593 | 431 | 175 |
|  |  |  | ALHF_03083.g1877 | 1242 | 99 | 92 |
|  |  |  | ALHF_08298.g5216 | 189 | 46 | 27 |
|  |  |  | ALHF_13859.g6829 | 32 | 12 | 10 |
|  |  |  | ALHF_08240.g5192 | 50 | 16 | 18 |
|  |  |  | ALHF_09204.g5573 | 20 | 2 | 3 |
|  |  |  | ALHF_00510.g280 | 15834 | 4604 | 3247 |
|  |  |  | ALHF_01609.g891 | 205 | 196 | 145 |
|  |  |  | ALHF_03990.g2563 | 10195 | 3275 | 436 |
|  |  |  | ALHF_11096.g6163 | 100 | 0 | 0 |
|  |  |  | ALHF_04020.g2585 | 3486 | 3479 | 831 |
|  |  |  | ALHF_02695.g1573 | 2316 | 1767 | 1167 |
|  |  |  | ALHF_03493.g2170 | 324 | 278 | 147 |
|  |  |  | ALHF_04025.g2590 | 107 | 26 | 8 |
|  |  |  | ALHF_03379.g2089 | 1478 | 381 | 327 |
|  |  |  | ALHF_05616.g3839 | 251 | 206 | 96 |
|  |  |  | ALHF_01744.g978 | 272 | 9 | 17 |
|  |  |  | ALHF_04879.g3309 | 5722 | 1827 | 765 |
|  |  |  | ALHF_11124.g6181 | 40 | 2 | 5 |
|  |  |  | ALHF_04276.g2801 | 285 | 243 | 139 |
|  |  |  | ALHF_06422.g4263 | 132 | 94 | 42 |
|  |  |  | ALHF_07600.g4881 | 108 | 16 | 7 |
|  |  |  | ALHF_01449.g792 | 83 | 13 | 28 |
|  |  |  | ALHF_02767.g1631 | 598 | 91 | 122 |
|  |  |  | ALHF_05161.g3523 | 6336 | 3813 | 1463 |
|  |  |  | ALHF_03691.g2320 | 876 | 424 | 119 |
|  |  |  | ALHF_10786.g6010 | 32 | 28 | 0 |
|  |  |  | ALHF_04738.g3184 | 999 | 593 | 241 |
|  |  |  | ALHF_06988.g4562 | 154 | 60 | 31 |
|  |  |  | ALHF_03112.g1898 | 205 | 20 | 1 |
|  |  |  | ALHF_11269.g6264 | 28 | 16 | 16 |
|  |  |  | ALHF_05109.g3487 | 1989 | 1164 | 289 |
|  |  |  | ALHF_11214.g6235 | 182 | 89 | 80 |
|  |  |  | ALHF_08149.g5151 | 609 | 254 | 176 |
|  |  |  | ALHF_10153.g5804 | 313 | 162 | 136 |
|  |  |  | ALHF_04812.g3251 | 500 | 264 | 133 |
|  |  |  | ALHF_04813.g3252 | 500 | 264 | 133 |
|  |  |  | ALHF_05775.g3934 | 757 | 619 | 353 |
|  |  |  | ALHF_09969.g5745 | 268 | 254 | 79 |
|  |  |  | ALHF_10384.g5874 | 268 | 254 | 79 |
|  |  |  | ALHF_08203.g5173 | 57 | 22 | 13 |
|  |  |  | ALHF_05923.g4017 | 270 | 186 | 238 |
|  |  |  | ALHF_03868.g2461 | 190 | 74 | 94 |
|  |  |  | ALHF_01025.g565 | 253 | 249 | 154 |
|  |  |  | ALHF_00366.g188 | 599 | 554 | 116 |
|  |  |  | ALHF_04924.g3348 | 1200 | 1094 | 94 |
|  |  |  | ALHF_07912.g5034 | 42 | 10 | 7 |
|  |  |  | ALHF_06828.g4478 | 379 | 75 | 201 |
|  |  |  | ALHF_01089.g602 | 187 | 72 | 15 |
|  |  |  | ALHF_05462.g3747 | 1753 | 988 | 213 |
|  |  |  | ALHF_08364.g5250 | 397 | 259 | 69 |
|  |  |  | ALHF_11876.g6677 | 3309 | 2984 | 1225 |
|  |  |  | ALHF_00296.g145 | 457 | 309 | 131 |
|  |  |  | ALHF_05316.g3649 | 2405 | 1746 | 469 |
|  |  |  | ALHF_10201.g5823 | 457 | 309 | 131 |
|  |  |  | ALHF_10202.g5824 | 1027 | 518 | 89 |
|  |  |  | ALHF_00078.g48 | 127 | 114 | 25 |
|  |  |  | ALHF_05036.g3426 | 1131 | 756 | 230 |
|  |  |  | ALHF_07835.g4994 | 239 | 122 | 36 |
|  |  |  | ALHF_10250.g5838 | 137 | 116 | 65 |
|  |  |  | ALHF_10485.g5903 | 351 | 138 | 32 |
|  |  |  | ALHF_05675.g3875 | 905 | 602 | 304 |
|  |  |  | ALHF_06398.g4250 | 317 | 309 | 223 |
|  |  |  | ALHF_10684.g5964 | 426 | 0 | 233 |
|  |  |  | ALHF_01380.g755 | 139 | 108 | 34 |
|  |  |  | ALHF_13018.g6794 | 95 | 80 | 10 |
|  |  |  | ALHF_08174.g5164 | 232 | 34 | 3 |
|  |  |  | ALHF_03780.g2393 | 3052 | 2876 | 1183 |
|  |  |  | ALHF_01901.g1073 | 1066 | 669 | 486 |
|  |  |  | ALHF_10315.g5855 | 171 | 165 | 79 |
|  |  |  | ALHF_04431.g2925 | 1034 | 142 | 4 |
|  |  |  | ALHF_03244.g1993 | 344 | 332 | 39 |
|  |  |  | ALHF_05388.g3700 | 1070 | 392 | 325 |
|  |  |  | ALHF_01355.g740 | 70 | 51 | 29 |
|  |  |  | ALHF_10606.g5945 | 40 | 39 | 33 |
|  |  |  | ALHF_11332.g6311 | 782 | 632 | 317 |
|  |  |  | ALHF_11366.g6338 | 782 | 632 | 317 |
|  |  |  | ALHF_02839.g1691 | 768 | 522 | 146 |
|  |  |  | ALHF_06521.g4313 | 241 | 191 | 58 |
|  |  |  | ALHF_11001.g6114 | 241 | 191 | 58 |
|  |  |  | ALHF_10827.g6029 | 699 | 430 | 172 |
|  |  |  | ALHF_10828.g6030 | 274 | 226 | 114 |
|  |  |  | ALHF_01545.g851 | 5811 | 5595 | 2071 |
|  |  |  | ALHF_10372.g5872 | 18 | 0 | 0 |
|  |  |  | ALHF_11556.g6467 | 1741 | 1263 | 1202 |
|  |  |  | ALHF_02093.g1191 | 788 | 264 | 115 |
|  |  |  | ALHF_03910.g2498 | 476 | 471 | 139 |
|  |  |  | ALHF_05148.g3516 | 4094 | 3911 | 2254 |
|  |  |  | ALHF_09132.g5544 | 112 | 28 | 16 |
|  |  |  | ALHF_10700.g5969 | 114 | 0 | 32 |
|  |  |  | ALHF_02038.g1155 | 3309 | 1211 | 1048 |
|  |  |  | ALHF_08419.g5272 | 108 | 30 | 18 |
|  |  |  | ALHF_10983.g6104 | 513 | 219 | 2 |
|  |  |  | ALHF_11602.g6500 | 218 | 21 | 207 |
|  |  |  | ALHF_04516.g3001 | 548 | 461 | 177 |
|  |  |  | ALHF_04814.g3253 | 903 | 856 | 449 |
|  |  |  | ALHF_02976.g1792 | 6 | 0 | 0 |
|  |  |  | ALHF_02978.g1794 | 6 | 0 | 0 |
|  |  |  | ALHF_11073.g6153 | 6 | 0 | 0 |
|  |  |  | ALHF_11074.g6154 | 6 | 0 | 0 |
|  |  |  | ALHF_11075.g6155 | 6 | 0 | 0 |
|  |  |  | ALHF_12097.g6744 | 6 | 0 | 0 |
|  |  |  | ALHF_05926.g4019 | 195 | 174 | 91 |
|  |  |  | ALHF_04273.g2798 | 1045 | 545 | 640 |
|  |  |  | ALHF_01396.g765 | 94 | 0 | 0 |
|  |  |  | ALHF_11786.g6623 | 89 | 0 | 0 |
|  |  |  | ALHF_01085.g599 | 954 | 279 | 120 |
|  |  |  | ALHF_08028.g5094 | 70 | 6 | 8 |
|  |  |  | ALHF_03380.g2090 | 1164 | 661 | 110 |
|  |  |  | ALHF_03848.g2445 | 947 | 841 | 239 |
|  |  |  | ALHF_11456.g6394 | 489 | 238 | 68 |
|  |  |  | ALHF_11470.g6405 | 560 | 0 | 85 |
|  |  |  |  |  |  |  |
| DOWN-REGULATED GENES | | |  |  |  |  |
| Extra-cellular processes | Blood clotting | Fibrinogen C-terminal domain-like | ALHF_11669.g6550 | 0 | 141 | 166 |
|  | Cell adhesion | Immunoglobulin | ALHF_00024.g17 | 83 | 330 | 710 |
|  |  |  | ALHF_00654.g365 | 360 | 1455 | 400 |
|  |  |  | ALHF_09531.g5674 | 21 | 90 | 163 |
|  |  |  | ALHF_09190.g5566 | 64 | 280 | 660 |
|  |  | Lamin A/C globular tail domain | ALHF_04723.g3169 | 616 | 3353 | 3558 |
|  |  | RNI-like | ALHF_02057.g1166 | 546 | 2696 | 4886 |
|  |  |  | ALHF_04627.g3093 | 608 | 3622 | 4540 |
|  |  | Sema domain | ALHF_00614.g342 | 912 | 3173 | 4100 |
|  |  |  | ALHF_08455.g5294 | 1192 | 4243 | 5734 |
|  |  | vWA-like | ALHF_04944.g3363 | 635 | 2672 | 2223 |
|  |  |  | ALHF_08748.g5411 | 44 | 452 | 643 |
|  | Immune response | Tetraspanin | ALHF_01683.g945 | 96 | 437 | 774 |
| General | General | ARM repeat | ALHF_00029.g23 | 140 | 548 | 719 |
|  |  |  | ALHF_00626.g348 | 439 | 2878 | 5164 |
|  |  |  | ALHF_00780.g427 | 300 | 2152 | 2750 |
|  |  |  | ALHF_00793.g435 | 496 | 1619 | 1920 |
|  |  |  | ALHF_00920.g511 | 225 | 2319 | 3478 |
|  |  |  | ALHF_01081.g597 | 119 | 432 | 125 |
|  |  |  | ALHF_01762.g988 | 155 | 954 | 1846 |
|  |  |  | ALHF_01814.g1018 | 564 | 2054 | 3572 |
|  |  |  | ALHF_02691.g1570 | 2074 | 9117 | 12432 |
|  |  |  | ALHF_02786.g1646 | 527 | 7083 | 11702 |
|  |  |  | ALHF_03038.g1844 | 429 | 2176 | 2067 |
|  |  |  | ALHF_03189.g1949 | 274 | 1565 | 2673 |
|  |  |  | ALHF_03259.g2004 | 595 | 2560 | 3341 |
|  |  |  | ALHF_03338.g2061 | 568 | 5769 | 9998 |
|  |  |  | ALHF_03415.g2116 | 1726 | 13634 | 14089 |
|  |  |  | ALHF_04238.g2765 | 2041 | 18980 | 19564 |
|  |  |  | ALHF_04425.g2920 | 2641 | 12360 | 14403 |
|  |  |  | ALHF_06221.g4158 | 589 | 2208 | 2708 |
|  |  |  | ALHF_06379.g4239 | 583 | 1927 | 3168 |
|  |  |  | ALHF_06431.g4266 | 193 | 798 | 1102 |
|  |  |  | ALHF_06745.g4434 | 97 | 421 | 807 |
|  |  |  | ALHF_07207.g4685 | 450 | 2576 | 3359 |
|  |  |  | ALHF_07277.g4715 | 215 | 2826 | 4480 |
|  |  |  | ALHF_07358.g4753 | 571 | 2070 | 3625 |
|  |  |  | ALHF_07544.g4851 | 574 | 2488 | 4240 |
|  |  |  | ALHF_07731.g4943 | 115 | 7033 | 17186 |
|  |  |  | ALHF_07809.g4987 | 74 | 429 | 724 |
|  |  |  | ALHF_07991.g5075 | 358 | 1283 | 1919 |
|  |  |  | ALHF_08107.g5133 | 750 | 2588 | 3428 |
|  |  |  | ALHF_08314.g5226 | 553 | 4402 | 5792 |
|  |  |  | ALHF_08367.g5252 | 149 | 1624 | 5292 |
|  |  |  | ALHF_08595.g5354 | 100 | 444 | 862 |
|  |  |  | ALHF_09187.g5564 | 586 | 2163 | 4778 |
|  |  |  | ALHF_09248.g5588 | 67 | 339 | 540 |
|  |  |  | ALHF_10713.g5975 | 1417 | 1695 | 1430 |
|  |  |  | ALHF_10822.g6026 | 598 | 9668 | 12988 |
|  |  |  | ALHF_00223.g108 | 171 | 598 | 988 |
|  |  |  | ALHF_01534.g842 | 210 | 833 | 1432 |
|  |  |  | ALHF_04148.g2693 | 843 | 3005 | 4705 |
|  |  |  | ALHF_08896.g5462 | 43 | 337 | 294 |
|  |  |  | ALHF_08993.g5498 | 142 | 592 | 760 |
|  |  |  | ALHF_10614.g5947 | 56 | 577 | 1018 |
|  |  | BRCT domain | ALHF_03403.g2109 | 786 | 3529 | 2926 |
|  |  | Calponin-homology domain, CH-domain | ALHF_02687.g1567 | 100 | 1820 | 4249 |
|  |  |  | ALHF_04247.g2774 | 696 | 4707 | 6716 |
|  |  |  | ALHF_11157.g6204 | 301 | 1187 | 2073 |
|  |  |  | ALHF_11501.g6432 | 1000 | 3360 | 3455 |
|  |  | EF-hand | ALHF_03515.g2178 | 190 | 661 | 805 |
|  |  |  | ALHF_04164.g2707 | 178 | 2217 | 3946 |
|  |  |  | ALHF_07891.g5020 | 213 | 795 | 1366 |
|  |  |  | ALHF_01475.g809 | 432 | 1434 | 2193 |
|  |  | Kelch motif | ALHF_01803.g1012 | 299 | 1124 | 1671 |
|  |  |  | ALHF_07393.g4775 | 140 | 589 | 927 |
|  |  |  | ALHF_09069.g5517 | 240 | 1019 | 2033 |
|  |  | L domain-like | ALHF_01704.g957 | 41 | 159 | 536 |
|  |  |  | ALHF_03024.g1831 | 132 | 648 | 137 |
|  |  |  | ALHF_05958.g4033 | 396 | 1388 | 1642 |
|  |  |  | ALHF_09197.g5569 | 120 | 611 | 872 |
|  |  |  | ALHF_01293.g707 | 283 | 1206 | 1658 |
|  |  |  | ALHF_03942.g2526 | 3471 | 3985 | 3610 |
|  |  | Ubiquitin-like | ALHF_01408.g771 | 51 | 193 | 309 |
|  |  |  | ALHF_02678.g1561 | 288 | 1090 | 1682 |
|  |  |  | ALHF_10091.g5791 | 130 | 566 | 1392 |
|  |  |  | ALHF_10685.g5965 | 51 | 193 | 309 |
|  |  | WD40 repeat-like | ALHF_00554.g313 | 187 | 621 | 1125 |
|  |  |  | ALHF_00572.g325 | 274 | 1570 | 3563 |
|  |  |  | ALHF_00742.g403 | 478 | 2060 | 3841 |
|  |  |  | ALHF_00804.g440 | 202 | 1050 | 1509 |
|  |  |  | ALHF_01079.g595 | 260 | 2252 | 2734 |
|  |  |  | ALHF_01250.g684 | 199 | 727 | 1145 |
|  |  |  | ALHF_01610.g892 | 243 | 1042 | 1313 |
|  |  |  | ALHF_01632.g908 | 55 | 628 | 679 |
|  |  |  | ALHF_01665.g932 | 270 | 1343 | 1781 |
|  |  |  | ALHF_02023.g1148 | 718 | 3021 | 3983 |
|  |  |  | ALHF_02051.g1164 | 775 | 3771 | 2692 |
|  |  |  | ALHF_02327.g1350 | 229 | 875 | 1380 |
|  |  |  | ALHF_02631.g1540 | 302 | 1247 | 1366 |
|  |  |  | ALHF_02868.g1714 | 93 | 522 | 720 |
|  |  |  | ALHF_03235.g1986 | 559 | 3096 | 5059 |
|  |  |  | ALHF_03682.g2312 | 359 | 1949 | 2099 |
|  |  |  | ALHF_04755.g3200 | 335 | 2347 | 2938 |
|  |  |  | ALHF_06127.g4114 | 794 | 6511 | 12499 |
|  |  |  | ALHF_06586.g4344 | 241 | 1858 | 2414 |
|  |  |  | ALHF_06670.g4392 | 335 | 1802 | 2751 |
|  |  |  | ALHF_06695.g4405 | 172 | 975 | 1589 |
|  |  |  | ALHF_06726.g4422 | 312 | 1756 | 3369 |
|  |  |  | ALHF_06795.g4457 | 388 | 1409 | 2534 |
|  |  |  | ALHF_07344.g4744 | 364 | 1684 | 2065 |
|  |  |  | ALHF_07483.g4828 | 861 | 3138 | 4856 |
|  |  |  | ALHF_08268.g5203 | 58 | 338 | 565 |
|  |  |  | ALHF_08326.g5234 | 214 | 1230 | 3842 |
|  |  |  | ALHF_08327.g5235 | 83 | 416 | 603 |
|  |  |  | ALHF_08509.g5314 | 212 | 1230 | 1983 |
|  |  |  | ALHF_09007.g5501 | 345 | 2960 | 4097 |
|  |  |  | ALHF_09014.g5503 | 100 | 712 | 1156 |
|  |  |  | ALHF_09129.g5543 | 89 | 904 | 1328 |
|  |  |  | ALHF_11167.g6210 | 246 | 1452 | 1962 |
|  |  |  | ALHF_00132.g70 | 262 | 1013 | 1123 |
|  |  |  | ALHF_01752.g982 | 243 | 1619 | 1947 |
|  |  |  | ALHF_02188.g1255 | 117 | 568 | 910 |
|  |  |  | ALHF_07771.g4962 | 95 | 411 | 673 |
|  |  |  | ALHF_07821.g4990 | 102 | 386 | 579 |
|  |  |  | ALHF_10537.g5925 | 833 | 5616 | 6713 |
|  | Ion binding | ArfGap/RecO-like zinc finger | ALHF_04457.g2949 | 404 | 1410 | 1375 |
|  |  |  | ALHF_08100.g5129 | 517 | 1846 | 2185 |
|  | Ligand binding | Supernatant protein factor (SPF), C-terminal domain | ALHF_02715.g1589 | 156 | 603 | 1043 |
|  |  |  | ALHF_08324.g5233 | 68 | 254 | 388 |
|  | Protein interaction | Ankyrin repeat | ALHF_01317.g719 | 207 | 886 | 1081 |
|  |  |  | ALHF_04762.g3207 | 230 | 914 | 2177 |
|  |  |  | ALHF_06753.g4439 | 213 | 1140 | 1605 |
|  |  |  | ALHF_07024.g4583 | 173 | 621 | 1206 |
|  |  |  | ALHF_07543.g4850 | 508 | 1759 | 2531 |
|  |  |  | ALHF_07768.g4960 | 91 | 353 | 504 |
|  |  |  | ALHF_09107.g5533 | 163 | 1034 | 2486 |
|  |  |  | ALHF_11618.g6509 | 217 | 751 | 883 |
|  |  | ApaG-like | ALHF_03522.g2186 | 1036 | 1176 | 1065 |
|  |  | BAR/IMD domain-like | ALHF_00310.g154 | 226 | 1800 | 2520 |
|  |  |  | ALHF_08173.g5163 | 235 | 1144 | 1727 |
|  |  |  | ALHF_11052.g6138 | 36 | 314 | 1139 |
|  |  |  | ALHF_11125.g6182 | 60 | 252 | 357 |
|  |  | FMN-linked oxidoreductases | ALHF_02535.g1479 | 641 | 5454 | 1865 |
|  |  | POZ domain | ALHF_00808.g442 | 208 | 749 | 890 |
|  |  |  | ALHF_00987.g544 | 791 | 3706 | 8025 |
|  |  |  | ALHF_02881.g1718 | 1361 | 6433 | 15502 |
|  |  |  | ALHF_06322.g4210 | 377 | 1813 | 3737 |
|  |  |  | ALHF_06411.g4256 | 86 | 526 | 782 |
|  |  |  | ALHF_03738.g2355 | 105 | 512 | 405 |
|  |  | SWIB/MDM2 domain | ALHF_02555.g1495 | 508 | 1738 | 3822 |
|  |  |  | ALHF_05762.g3925 | 227 | 1555 | 3850 |
|  |  | TPR-like | ALHF_00127.g67 | 44 | 203 | 344 |
|  |  |  | ALHF_00356.g181 | 389 | 1277 | 1950 |
|  |  |  | ALHF_00574.g327 | 567 | 2596 | 5738 |
|  |  |  | ALHF_01270.g694 | 235 | 926 | 1527 |
|  |  |  | ALHF_01794.g1006 | 891 | 4426 | 5401 |
|  |  |  | ALHF_01802.g1011 | 126 | 518 | 921 |
|  |  |  | ALHF_02622.g1532 | 591 | 2782 | 3715 |
|  |  |  | ALHF_03007.g1817 | 528 | 2134 | 2095 |
|  |  |  | ALHF_04881.g3311 | 823 | 2789 | 2522 |
|  |  |  | ALHF_06134.g4116 | 474 | 2334 | 3405 |
|  |  |  | ALHF_06486.g4294 | 667 | 2649 | 4663 |
|  |  |  | ALHF_06495.g4299 | 401 | 1400 | 2085 |
|  |  |  | ALHF_06959.g4546 | 377 | 2256 | 3628 |
|  |  |  | ALHF_07587.g4874 | 271 | 924 | 1699 |
|  |  |  | ALHF_08061.g5111 | 96 | 465 | 883 |
|  |  |  | ALHF_08656.g5378 | 103 | 446 | 919 |
|  |  |  | ALHF_08781.g5424 | 60 | 301 | 388 |
|  |  | UBA-like | ALHF_03586.g2238 | 199 | 1411 | 2613 |
|  |  |  | ALHF_03588.g2240 | 199 | 1411 | 2613 |
|  |  |  | ALHF_11282.g6273 | 28 | 396 | 767 |
|  |  |  | ALHF_11286.g6277 | 28 | 396 | 767 |
|  |  | WW domain | ALHF_08426.g5276 | 140 | 619 | 1823 |
|  |  | WWE domain | ALHF_09200.g5571 | 207 | 972 | 2497 |
|  | Small molecule binding | GST C-terminal domain-like | ALHF_07154.g4655 | 123 | 707 | 745 |
|  |  | NAD(P)-binding Rossmann-fold domains | ALHF_02254.g1306 | 143 | 500 | 752 |
|  |  |  | ALHF_03060.g1857 | 1020 | 16870 | 3550 |
|  |  |  | ALHF_03614.g2258 | 393 | 2026 | 5051 |
|  |  |  | ALHF_07462.g4815 | 158 | 716 | 1036 |
|  |  | P-loop containing nucleoside triphosphate hydrolases | ALHF_00816.g447 | 116 | 412 | 694 |
|  |  |  | ALHF_00846.g465 | 830 | 3721 | 3776 |
|  |  |  | ALHF_00919.g510 | 571 | 2085 | 3536 |
|  |  |  | ALHF_00998.g548 | 658 | 2364 | 2858 |
|  |  |  | ALHF_01472.g807 | 122 | 998 | 1086 |
|  |  |  | ALHF_01687.g948 | 520 | 2505 | 3902 |
|  |  |  | ALHF_01823.g1026 | 362 | 1291 | 2859 |
|  |  |  | ALHF_01896.g1071 | 1210 | 6954 | 8072 |
|  |  |  | ALHF_02073.g1180 | 426 | 2178 | 5020 |
|  |  |  | ALHF_02479.g1441 | 373 | 1238 | 1982 |
|  |  |  | ALHF_02547.g1488 | 963 | 4659 | 4765 |
|  |  |  | ALHF_02702.g1578 | 1910 | 8177 | 15854 |
|  |  |  | ALHF_02703.g1579 | 556 | 2621 | 4036 |
|  |  |  | ALHF_02739.g1610 | 383 | 1849 | 3105 |
|  |  |  | ALHF_02798.g1655 | 607 | 2045 | 3009 |
|  |  |  | ALHF_03344.g2065 | 676 | 5033 | 10583 |
|  |  |  | ALHF_03383.g2092 | 400 | 1759 | 2021 |
|  |  |  | ALHF_03456.g2141 | 178 | 743 | 712 |
|  |  |  | ALHF_03477.g2157 | 1162 | 14058 | 18714 |
|  |  |  | ALHF_03545.g2204 | 346 | 1314 | 2464 |
|  |  |  | ALHF_03670.g2303 | 290 | 2065 | 2823 |
|  |  |  | ALHF_03831.g2431 | 1484 | 6983 | 12262 |
|  |  |  | ALHF_04188.g2729 | 481 | 1737 | 1892 |
|  |  |  | ALHF_04387.g2888 | 799 | 4227 | 8982 |
|  |  |  | ALHF_04717.g3164 | 433 | 1678 | 2169 |
|  |  |  | ALHF_04797.g3237 | 709 | 3909 | 2580 |
|  |  |  | ALHF_04880.g3310 | 1130 | 6454 | 7543 |
|  |  |  | ALHF_04919.g3343 | 1380 | 4765 | 4306 |
|  |  |  | ALHF_04943.g3362 | 4776 | 16787 | 15492 |
|  |  |  | ALHF_05298.g3633 | 5061 | 26160 | 30186 |
|  |  |  | ALHF_05742.g3913 | 1498 | 4861 | 6278 |
|  |  |  | ALHF_06006.g4056 | 147 | 720 | 1224 |
|  |  |  | ALHF_06235.g4166 | 330 | 2349 | 3095 |
|  |  |  | ALHF_06340.g4221 | 481 | 2971 | 4323 |
|  |  |  | ALHF_06343.g4222 | 102 | 543 | 530 |
|  |  |  | ALHF_06372.g4237 | 210 | 1468 | 2368 |
|  |  |  | ALHF_06434.g4267 | 267 | 1898 | 2723 |
|  |  |  | ALHF_06672.g4394 | 957 | 3160 | 8097 |
|  |  |  | ALHF_06696.g4406 | 182 | 984 | 2079 |
|  |  |  | ALHF_06739.g4429 | 380 | 2022 | 4811 |
|  |  |  | ALHF_06772.g4447 | 364 | 1976 | 2901 |
|  |  |  | ALHF_06845.g4492 | 778 | 2843 | 5513 |
|  |  |  | ALHF_06912.g4522 | 401 | 1713 | 2087 |
|  |  |  | ALHF_07063.g4608 | 69 | 1945 | 3603 |
|  |  |  | ALHF_07146.g4650 | 287 | 1627 | 4098 |
|  |  |  | ALHF_07187.g4674 | 368 | 1441 | 2569 |
|  |  |  | ALHF_07314.g4731 | 229 | 1489 | 1894 |
|  |  |  | ALHF_07332.g4737 | 256 | 1165 | 1621 |
|  |  |  | ALHF_07342.g4743 | 283 | 938 | 1397 |
|  |  |  | ALHF_07398.g4778 | 81 | 307 | 428 |
|  |  |  | ALHF_07504.g4833 | 371 | 1636 | 2153 |
|  |  |  | ALHF_07560.g4860 | 69 | 481 | 1562 |
|  |  |  | ALHF_07681.g4916 | 121 | 624 | 1144 |
|  |  |  | ALHF_07706.g4928 | 77 | 289 | 450 |
|  |  |  | ALHF_07736.g4945 | 124 | 467 | 1194 |
|  |  |  | ALHF_07964.g5058 | 587 | 2882 | 3296 |
|  |  |  | ALHF_07974.g5065 | 626 | 3071 | 3720 |
|  |  |  | ALHF_08065.g5113 | 160 | 883 | 1893 |
|  |  |  | ALHF_08164.g5158 | 74 | 459 | 980 |
|  |  |  | ALHF_08328.g5236 | 220 | 1203 | 2199 |
|  |  |  | ALHF_08424.g5275 | 22 | 158 | 510 |
|  |  |  | ALHF_08436.g5282 | 194 | 1176 | 882 |
|  |  |  | ALHF_08469.g5300 | 113 | 403 | 503 |
|  |  |  | ALHF_08755.g5415 | 22 | 212 | 553 |
|  |  |  | ALHF_08872.g5455 | 429 | 1897 | 3539 |
|  |  |  | ALHF_09208.g5575 | 87 | 318 | 356 |
|  |  |  | ALHF_09383.g5631 | 107 | 870 | 1341 |
|  |  |  | ALHF_09504.g5665 | 62 | 273 | 224 |
|  |  |  | ALHF_10996.g6109 | 1306 | 5339 | 8222 |
|  |  |  | ALHF_11037.g6131 | 305 | 1161 | 1013 |
|  |  |  | ALHF_11450.g6391 | 284 | 1040 | 1445 |
|  |  |  | ALHF_11693.g6559 | 284 | 1040 | 1445 |
|  |  |  | ALHF_11756.g6605 | 729 | 3282 | 3542 |
|  |  |  | ALHF_00040.g28 | 48 | 215 | 467 |
|  |  |  | ALHF_00095.g56 | 98 | 457 | 604 |
|  |  |  | ALHF_00985.g543 | 480 | 1932 | 4436 |
|  |  |  | ALHF_01746.g979 | 558 | 2383 | 3300 |
|  |  |  | ALHF_02657.g1552 | 206 | 1979 | 2994 |
|  |  |  | ALHF_04233.g2760 | 376 | 1743 | 1915 |
|  |  |  | ALHF_04454.g2946 | 536 | 2289 | 2082 |
|  |  |  | ALHF_07018.g4579 | 140 | 914 | 1244 |
|  |  |  | ALHF_07377.g4765 | 75 | 721 | 922 |
|  |  |  | ALHF_08490.g5304 | 61 | 377 | 368 |
|  |  |  | ALHF_09477.g5657 | 61 | 377 | 458 |
|  |  |  | ALHF_13359.g6809 | 14 | 177 | 304 |
| Information | Chromatin structure | NAP-like | ALHF_01276.g699 | 3799 | 22764 | 23990 |
|  |  | Nucleoplasmin-like core domain | ALHF_02215.g1272 | 205 | 1283 | 2147 |
|  |  | Smc hinge domain | ALHF_06490.g4297 | 794 | 3458 | 5785 |
|  | DNA replication/repair | Chromo domain-like | ALHF_00781.g428 | 75 | 461 | 1044 |
|  |  |  | ALHF_01090.g603 | 154 | 587 | 1080 |
|  |  |  | ALHF_05481.g3761 | 373 | 1633 | 2589 |
|  |  |  | ALHF_06020.g4062 | 372 | 1234 | 1805 |
|  |  |  | ALHF_07353.g4750 | 98 | 352 | 552 |
|  |  | DNA clamp | ALHF_00768.g420 | 347 | 2694 | 5306 |
|  |  | DNase I-like | ALHF_07205.g4684 | 300 | 2034 | 3875 |
|  |  |  | ALHF_07222.g4690 | 112 | 1482 | 3582 |
|  |  | FYVE/PHD zinc finger | ALHF_00602.g336 | 55 | 213 | 538 |
|  |  |  | ALHF_01654.g925 | 312 | 1604 | 3138 |
|  |  |  | ALHF_02449.g1423 | 383 | 1596 | 2616 |
|  |  |  | ALHF_03565.g2220 | 285 | 990 | 954 |
|  |  |  | ALHF_07108.g4635 | 1344 | 5157 | 10581 |
|  |  |  | ALHF_08443.g5287 | 42 | 234 | 530 |
|  |  |  | ALHF_08567.g5339 | 106 | 400 | 765 |
|  |  |  | ALHF_08759.g5417 | 258 | 3850 | 2511 |
|  |  |  | ALHF_11298.g6287 | 140 | 747 | 843 |
|  |  |  | ALHF_01233.g674 | 174 | 803 | 1656 |
|  |  |  | ALHF_07182.g4671 | 86 | 383 | 489 |
|  |  | His-Me finger endonucleases | ALHF_00518.g286 | 1230 | 33015 | 4604 |
|  |  | Nucleic acid-binding proteins | ALHF_00252.g119 | 91 | 632 | 1514 |
|  |  |  | ALHF_01244.g680 | 355 | 3038 | 7361 |
|  |  |  | ALHF_01876.g1059 | 8462 | 31371 | 27586 |
|  |  |  | ALHF_02131.g1218 | 365 | 5082 | 12310 |
|  |  |  | ALHF_03113.g1899 | 354 | 3160 | 8586 |
|  |  |  | ALHF_03637.g2278 | 250 | 1050 | 1539 |
|  |  |  | ALHF_05023.g3418 | 784 | 3480 | 4842 |
|  |  |  | ALHF_06193.g4140 | 436 | 1915 | 5052 |
|  |  |  | ALHF_07015.g4577 | 282 | 4656 | 9672 |
|  |  |  | ALHF_07846.g5001 | 78 | 286 | 691 |
|  |  |  | ALHF_06203.g4147 | 273 | 3326 | 6713 |
|  |  |  | ALHF_06803.g4463 | 194 | 741 | 903 |
|  |  | Restriction endonuclease-like | ALHF_08636.g5371 | 111 | 457 | 884 |
|  |  | RING/U-box | ALHF_02504.g1460 | 97 | 337 | 503 |
|  |  |  | ALHF_02744.g1614 | 105 | 414 | 769 |
|  |  |  | ALHF_06067.g4085 | 157 | 563 | 968 |
|  |  |  | ALHF_06684.g4401 | 224 | 1536 | 2109 |
|  |  |  | ALHF_07121.g4642 | 391 | 2044 | 2934 |
|  |  |  | ALHF_07387.g4770 | 283 | 1082 | 1505 |
|  |  |  | ALHF_07776.g4964 | 174 | 1198 | 2216 |
|  |  |  | ALHF_07998.g5080 | 175 | 990 | 3107 |
|  |  |  | ALHF_08129.g5140 | 396 | 1660 | 3017 |
|  |  |  | ALHF_08269.g5204 | 106 | 767 | 1480 |
|  |  |  | ALHF_10878.g6055 | 97 | 337 | 503 |
|  |  |  | ALHF_09211.g5576 | 99 | 425 | 849 |
|  |  | Tudor/PWWP/MBT | ALHF_01126.g622 | 341 | 1612 | 3503 |
|  |  |  | ALHF_01336.g728 | 423 | 1877 | 2740 |
|  |  |  | ALHF_01569.g864 | 370 | 1713 | 2979 |
|  |  |  | ALHF_02008.g1137 | 573 | 2679 | 2111 |
|  |  |  | ALHF_03034.g1840 | 2743 | 9994 | 11510 |
|  |  |  | ALHF_06653.g4382 | 158 | 650 | 602 |
|  |  |  | ALHF_06839.g4486 | 115 | 600 | 908 |
|  |  |  | ALHF_07171.g4664 | 366 | 2617 | 2582 |
|  |  |  | ALHF_08087.g5126 | 53 | 476 | 683 |
|  |  |  | ALHF_12079.g6738 | 207 | 731 | 1387 |
|  |  |  | ALHF_12041.g6732 | 174 | 598 | 494 |
|  |  | Type II DNA topoisomerase | ALHF_02096.g1193 | 1599 | 9401 | 19808 |
|  | RNA processing | Eukaryotic type KH-domain (KH-domain type I) | ALHF_00352.g177 | 382 | 1243 | 2808 |
|  |  |  | ALHF_00738.g401 | 110 | 765 | 1122 |
|  |  |  | ALHF_05826.g3960 | 543 | 2008 | 2680 |
|  |  |  | ALHF_06541.g4324 | 376 | 1823 | 3710 |
|  |  |  | ALHF_06984.g4560 | 217 | 872 | 1471 |
|  |  |  | ALHF_07904.g5028 | 84 | 555 | 886 |
|  |  |  | ALHF_10654.g5955 | 0 | 216 | 154 |
|  |  | ISY1 domain-like | ALHF_08379.g5254 | 31 | 221 | 378 |
|  |  | PAP/OAS1 substrate-binding domain | ALHF_09123.g5540 | 104 | 1101 | 2184 |
|  |  | RNase III domain-like | ALHF_00102.g58 | 560 | 2368 | 1587 |
|  |  | Translin | ALHF_05928.g4021 | 129 | 487 | 563 |
|  | Transcription | beta and beta-prime subunits of DNA dependent RNA-polymerase | ALHF_04866.g3298 | 1048 | 6352 | 10122 |
|  |  |  | ALHF_04901.g3329 | 2049 | 9340 | 19454 |
|  |  |  | ALHF_06824.g4475 | 489 | 2286 | 2686 |
|  |  |  | ALHF_07315.g4732 | 187 | 1112 | 1155 |
|  |  |  | ALHF_08449.g5291 | 80 | 517 | 806 |
|  |  |  | ALHF_12792.g6783 | 28 | 162 | 297 |
|  |  |  | ALHF_08104.g5132 | 34 | 219 | 317 |
|  |  | E2F-DP heterodimerization region | ALHF_09357.g5622 | 314 | 1618 | 3112 |
|  |  |  | ALHF_08184.g5166 | 85 | 628 | 1057 |
|  |  | Eukaryotic RPB5 N-terminal domain | ALHF_02159.g1234 | 206 | 752 | 1589 |
|  |  | Poly A polymerase C-terminal region-like | ALHF_00719.g391 | 137 | 667 | 1047 |
|  | Translation | Class II aaRS ABD-related | ALHF_01194.g654 | 132 | 912 | 1412 |
|  |  |  | ALHF_01670.g935 | 230 | 1178 | 2062 |
|  |  |  | ALHF_08227.g5184 | 62 | 252 | 510 |
|  |  | eEF1-gamma domain | ALHF_03541.g2201 | 16947 | 54980 | 48923 |
|  |  | eIF-2-alpha, C-terminal domain | ALHF_04903.g3331 | 934 | 3969 | 4324 |
|  |  | eIF4e-like | ALHF_03298.g2028 | 785 | 4110 | 5820 |
|  |  |  | ALHF_07243.g4695 | 316 | 1283 | 2471 |
|  |  | Elongation factor TFIIS domain 2 | ALHF_01565.g863 | 180 | 1089 | 1774 |
|  |  | Functional domain of the splicing factor Prp18 | ALHF_07841.g4999 | 69 | 307 | 389 |
|  |  | Hsp90 co-chaperone CDC37 | ALHF_00640.g357 | 586 | 1968 | 2349 |
|  |  | L30e-like | ALHF_06215.g4153 | 85 | 480 | 991 |
|  |  | Release factor | ALHF_09657.g5700 | 23 | 128 | 160 |
|  |  | Ribosomal protein L1 | ALHF_01091.g604 | 251 | 1845 | 2538 |
|  |  | Ribosomal protein S5 domain 2-like | ALHF_00678.g376 | 1405 | 6480 | 5480 |
|  |  |  | ALHF_02986.g1799 | 9496 | 47007 | 72512 |
|  |  |  | ALHF_05789.g3941 | 869 | 2979 | 3454 |
|  |  | Ribosome recycling factor, RRF | ALHF_07575.g4867 | 51 | 258 | 374 |
|  |  | Sm-like ribonucleoproteins | ALHF_01205.g660 | 46 | 411 | 775 |
|  |  |  | ALHF_03248.g1997 | 755 | 21511 | 44860 |
|  |  |  | ALHF_06851.g4494 | 33 | 132 | 269 |
|  |  | Translation initiation factor 2 beta, aIF2beta, N-terminal domain | ALHF_00525.g292 | 893 | 3743 | 4653 |
|  |  | Translation proteins | ALHF_02583.g1511 | 176 | 898 | 1080 |
|  |  | Translational machinery components | ALHF_03482.g2160 | 297 | 1044 | 1221 |
| Intra-cellular processes | Cell cycle, Apoptosis | Bcl-2 inhibitors of programmed cell death | ALHF_03724.g2345 | 140 | 701 | 979 |
|  |  | Cullin repeat-like | ALHF_05747.g3916 | 1150 | 5037 | 8044 |
|  |  |  | ALHF_06812.g4469 | 216 | 778 | 999 |
|  |  | Cystine-knot cytokines | ALHF_05915.g4011 | 239 | 929 | 1191 |
|  |  | Mitotic arrest deficient-like 1, Mad1 | ALHF_08155.g5153 | 145 | 667 | 1557 |
|  |  | RCC1/BLIP-II | ALHF_05760.g3923 | 427 | 3409 | 5085 |
|  |  |  | ALHF_05877.g3985 | 561 | 5193 | 9123 |
|  |  | Rhodanese/Cell cycle control phosphatase | ALHF_00471.g252 | 138 | 1005 | 932 |
|  | Cell motility | Actin depolymerizing proteins | ALHF_03938.g2522 | 428 | 1711 | 2042 |
|  |  | Mitochondrial glycoprotein MAM33-like | ALHF_05623.g3844 | 364 | 2885 | 4720 |
|  |  | Outer arm dynein light chain 1 | ALHF_11377.g6345 | 0 | 97 | 59 |
|  |  | Tropomyosin | ALHF_03492.g2169 | 561 | 641 | 592 |
|  |  | Tubulin nucleotide-binding domain-like | ALHF_04988.g3387 | 99 | 12418 | 20943 |
|  |  | VHP, Villin headpiece domain | ALHF_08275.g5208 | 172 | 901 | 1706 |
|  | Ion m/tr | Band 7/SPFH domain | ALHF_00692.g384 | 88 | 367 | 520 |
|  |  |  | ALHF_03719.g2342 | 730 | 3442 | 3904 |
|  |  |  | ALHF_05423.g3722 | 677 | 3024 | 3455 |
|  |  |  | ALHF_06017.g4060 | 348 | 1204 | 1323 |
|  |  | Calcium ATPase, transmembrane domain M | ALHF_01140.g626 | 1343 | 4431 | 4273 |
|  |  | Cupredoxins | ALHF_00409.g214 | 235 | 800 | 903 |
|  |  | Ferritin-like | ALHF_01191.g652 | 564 | 6452 | 13798 |
|  |  | MFS general substrate transporter | ALHF_02775.g1637 | 208 | 3349 | 3316 |
|  |  |  | ALHF_03002.g1812 | 549 | 2233 | 2259 |
|  |  |  | ALHF_03748.g2367 | 974 | 4383 | 2982 |
|  |  |  | ALHF_05854.g3973 | 337 | 2456 | 2270 |
|  |  |  | ALHF_06312.g4206 | 246 | 958 | 763 |
|  |  |  | ALHF_06625.g4366 | 182 | 2186 | 4104 |
|  |  |  | ALHF_08342.g5240 | 283 | 2178 | 1510 |
|  |  |  | ALHF_08448.g5290 | 173 | 698 | 636 |
|  |  |  | ALHF_08649.g5374 | 143 | 572 | 1154 |
|  |  | Multidrug resistance efflux transporter EmrE | ALHF_06635.g4369 | 187 | 682 | 775 |
|  |  | Periplasmic binding protein-like II | ALHF_11359.g6330 | 466 | 8792 | 1376 |
|  |  | SET domain | ALHF_03458.g2143 | 1302 | 5142 | 7048 |
|  |  |  | ALHF_06302.g4200 | 337 | 1121 | 1621 |
|  |  |  | ALHF_06808.g4466 | 267 | 1885 | 2860 |
|  |  |  | ALHF_07287.g4720 | 243 | 1346 | 2691 |
|  |  |  | ALHF_08255.g5197 | 47 | 511 | 438 |
|  |  |  | ALHF_08492.g5305 | 276 | 1550 | 6311 |
|  |  |  | ALHF_08658.g5380 | 137 | 1364 | 3210 |
|  |  |  | ALHF_08817.g5436 | 220 | 725 | 1203 |
|  |  |  | ALHF_07984.g5071 | 92 | 328 | 575 |
|  |  |  | ALHF_09266.g5595 | 78 | 402 | 980 |
|  | Phospholipid m/tr | CRAL/TRIO domain | ALHF_07179.g4669 | 122 | 1257 | 128 |
|  |  | PLC-like phosphodiesterases | ALHF_07992.g5076 | 391 | 1950 | 2599 |
|  | Proteases | Caspase-like | ALHF_06331.g4217 | 75 | 449 | 878 |
|  |  | ClpP/crotonase | ALHF_01922.g1086 | 485 | 3532 | 7276 |
|  |  |  | ALHF_03535.g2195 | 1652 | 2062 | 1842 |
|  |  | Creatinase/aminopeptidase | ALHF_03743.g2361 | 1454 | 5232 | 5558 |
|  |  |  | ALHF_04397.g2897 | 1995 | 2543 | 2188 |
|  |  |  | ALHF_04947.g3365 | 1637 | 10598 | 13635 |
|  |  |  | ALHF_10591.g5939 | 350 | 3789 | 6521 |
|  |  | Cysteine proteinases | ALHF_00253.g120 | 2674 | 8628 | 8648 |
|  |  |  | ALHF_02424.g1407 | 193 | 806 | 1371 |
|  |  |  | ALHF_02944.g1766 | 750 | 3935 | 5457 |
|  |  |  | ALHF_03630.g2271 | 501 | 3840 | 8043 |
|  |  |  | ALHF_03883.g2473 | 808 | 3420 | 2683 |
|  |  |  | ALHF_04503.g2988 | 576 | 3606 | 5825 |
|  |  |  | ALHF_06573.g4338 | 657 | 2183 | 3246 |
|  |  |  | ALHF_07167.g4662 | 299 | 1358 | 2187 |
|  |  |  | ALHF_07696.g4923 | 265 | 2096 | 4348 |
|  |  |  | ALHF_07805.g4984 | 97 | 401 | 510 |
|  |  |  | ALHF_08137.g5146 | 595 | 2705 | 6237 |
|  |  |  | ALHF_06182.g4134 | 280 | 1118 | 1623 |
|  |  | Leukotriene A4 hydrolase N-terminal domain | ALHF_08357.g5248 | 86 | 364 | 558 |
|  |  | LuxS/MPP-like metallohydrolase | ALHF_02340.g1356 | 868 | 4119 | 5784 |
|  |  |  | ALHF_06744.g4433 | 678 | 2369 | 3122 |
|  |  | Metallo-dependent phosphatases | ALHF_00751.g410 | 202 | 1371 | 3909 |
|  |  |  | ALHF_03030.g1836 | 462 | 2167 | 2884 |
|  |  |  | ALHF_11597.g6494 | 523 | 2333 | 4314 |
|  |  | Metalloproteases ("zincins"), catalytic domain | ALHF_00632.g352 | 637 | 2672 | 712 |
|  |  |  | ALHF_04383.g2884 | 1506 | 7624 | 10355 |
|  |  |  | ALHF_05541.g3801 | 846 | 7451 | 2808 |
|  |  |  | ALHF_05699.g3892 | 1199 | 4855 | 5914 |
|  |  |  | ALHF_08437.g5283 | 222 | 878 | 1182 |
|  |  | Serpins | ALHF_02165.g1239 | 581 | 2425 | 2850 |
|  |  |  | ALHF_05524.g3788 | 1093 | 5022 | 3749 |
|  |  | Subtilisin-like | ALHF_02191.g1257 | 1306 | 6776 | 5386 |
|  |  |  | ALHF_06079.g4092 | 231 | 917 | 1194 |
|  |  | Tricorn protease domain 2 | ALHF_00951.g526 | 276 | 1092 | 1361 |
|  |  |  | ALHF_01184.g648 | 82 | 1572 | 2641 |
|  |  |  | ALHF_03004.g1814 | 515 | 3938 | 5439 |
|  |  |  | ALHF_05093.g3472 | 4514 | 16768 | 17538 |
|  |  |  | ALHF_05788.g3940 | 436 | 1693 | 2394 |
|  |  |  | ALHF_05959.g4034 | 351 | 1229 | 1416 |
|  |  |  | ALHF_07820.g4989 | 96 | 478 | 528 |
|  |  |  | ALHF_08044.g5101 | 84 | 607 | 896 |
|  |  |  | ALHF_08102.g5131 | 188 | 950 | 1532 |
|  |  |  | ALHF_08570.g5341 | 88 | 781 | 1215 |
|  |  |  | ALHF_08763.g5418 | 303 | 2074 | 9741 |
|  |  |  | ALHF_06365.g4233 | 361 | 2289 | 3423 |
|  |  | Trypsin-like serine proteases | ALHF_00567.g323 | 3472 | 14748 | 3706 |
|  |  |  | ALHF_02036.g1154 | 401 | 4410 | 1508 |
|  |  |  | ALHF_02287.g1327 | 14458 | 47995 | 14531 |
|  |  |  | ALHF_06531.g4319 | 122 | 520 | 761 |
|  |  | Zn-dependent exopeptidases | ALHF_03052.g1852 | 122 | 523 | 130 |
|  |  |  | ALHF_03254.g2002 | 490 | 2808 | 2773 |
|  | Protein modification | Chaperone J-domain | ALHF_00376.g194 | 4801 | 15669 | 16428 |
|  |  |  | ALHF_05764.g3927 | 377 | 1378 | 1551 |
|  |  |  | ALHF_06139.g4118 | 480 | 2434 | 3968 |
|  |  |  | ALHF_07361.g4754 | 160 | 2440 | 961 |
|  |  |  | ALHF_07741.g4946 | 539 | 2242 | 3670 |
|  |  |  | ALHF_08543.g5329 | 92 | 348 | 552 |
|  |  |  | ALHF_09735.g5706 | 89 | 754 | 1477 |
|  |  |  | ALHF_10943.g6081 | 44 | 492 | 343 |
|  |  | Cyclophilin-like | ALHF_05323.g3655 | 1420 | 5843 | 6079 |
|  |  | FKBP-like | ALHF_00613.g341 | 470 | 4418 | 6687 |
|  |  |  | ALHF_06748.g4437 | 211 | 1369 | 1945 |
|  |  |  | ALHF_11239.g6248 | 183 | 2164 | 2143 |
|  |  | GroEL equatorial domain-like | ALHF_02584.g1512 | 2238 | 10314 | 7848 |
|  |  |  | ALHF_02913.g1741 | 2209 | 11577 | 12697 |
|  |  |  | ALHF_03015.g1824 | 2189 | 8434 | 9511 |
|  |  |  | ALHF_04102.g2652 | 1557 | 9973 | 12859 |
|  |  |  | ALHF_04708.g3160 | 1397 | 7199 | 7818 |
|  |  |  | ALHF_04744.g3189 | 1794 | 12408 | 14144 |
|  |  |  | ALHF_05441.g3734 | 1401 | 8652 | 10513 |
|  |  |  | ALHF_05461.g3746 | 1606 | 7307 | 8691 |
|  |  |  | ALHF_05565.g3814 | 1223 | 9344 | 8301 |
|  |  | HSP20-like chaperones | ALHF_00299.g147 | 96 | 989 | 642 |
|  |  |  | ALHF_01116.g617 | 155 | 1850 | 1658 |
|  |  |  | ALHF_01169.g640 | 146 | 888 | 1606 |
|  |  |  | ALHF_02106.g1200 | 184 | 2338 | 1401 |
|  |  |  | ALHF_04411.g2908 | 227 | 1699 | 1557 |
|  |  |  | ALHF_05408.g3713 | 573 | 3541 | 6310 |
|  |  |  | ALHF_05869.g3979 | 153 | 2594 | 2363 |
|  |  |  | ALHF_07252.g4702 | 55 | 705 | 488 |
|  |  |  | ALHF_11599.g6496 | 143 | 705 | 950 |
|  |  |  | ALHF_12637.g6776 | 37 | 271 | 428 |
|  |  | Mannose 6-phosphate receptor domain | ALHF_04775.g3219 | 750 | 2494 | 2528 |
|  |  | Prefoldin | ALHF_06234.g4165 | 164 | 846 | 1482 |
|  |  |  | ALHF_12876.g6787 | 38 | 200 | 254 |
|  |  | Proteasome activator | ALHF_04492.g2979 | 331 | 1849 | 2735 |
|  |  | UBC-like | ALHF_00052.g33 | 209 | 3034 | 5661 |
|  |  |  | ALHF_00279.g134 | 421 | 1514 | 2453 |
|  |  |  | ALHF_00538.g302 | 168 | 583 | 945 |
|  |  |  | ALHF_04042.g2605 | 619 | 3068 | 3924 |
|  |  |  | ALHF_05367.g3686 | 595 | 753 | 650 |
|  |  |  | ALHF_05518.g3784 | 288 | 1135 | 1761 |
|  |  |  | ALHF_06296.g4196 | 198 | 838 | 1621 |
|  | Transport | C-terminal autoproteolytic domain of nucleoporin nup98 | ALHF_00443.g233 | 972 | 5463 | 7849 |
|  |  | ENTH/VHS domain | ALHF_06301.g4199 | 321 | 1380 | 1897 |
|  |  | Lipocalins | ALHF_00460.g244 | 183 | 932 | 763 |
|  |  | MIT domain | ALHF_08511.g5316 | 81 | 318 | 468 |
|  |  | Mitochondrial carrier | ALHF_01048.g578 | 118 | 541 | 741 |
|  |  |  | ALHF_01514.g830 | 228 | 1084 | 978 |
|  |  | NTF2-like | ALHF_02109.g1203 | 280 | 1709 | 3096 |
|  |  |  | ALHF_04094.g2645 | 1027 | 6984 | 10685 |
|  |  | Nucleoporin domain | ALHF_09293.g5602 | 721 | 4164 | 6444 |
|  |  | Phoshotransferase/anion transport protein | ALHF_06646.g4376 | 656 | 2221 | 2031 |
|  |  |  | ALHF_06836.g4484 | 761 | 2785 | 2190 |
|  |  | SNARE fusion complex | ALHF_00217.g105 | 93 | 355 | 421 |
|  |  |  | ALHF_02090.g1188 | 86 | 477 | 619 |
|  |  | SRP19 | ALHF_00391.g205 | 137 | 521 | 575 |
| Metabolism | Amino acids m/tr | Arginase/deacetylase | ALHF_00776.g424 | 318 | 1930 | 4797 |
|  |  | Glutaminase/Asparaginase | ALHF_01030.g569 | 313 | 1959 | 1385 |
|  |  | PLP-binding barrel | ALHF_12502.g6766 | 71 | 1052 | 255 |
|  | Carbohydrate m/tr | (Trans)glycosidases | ALHF_06583.g4343 | 178 | 734 | 789 |
|  |  |  | ALHF_08664.g5383 | 268 | 1252 | 2196 |
|  |  | HIT-like | ALHF_00061.g36 | 107 | 383 | 1160 |
|  |  | Seven-hairpin glycosidases | ALHF_07726.g4940 | 241 | 892 | 1071 |
|  |  |  | ALHF_08699.g5395 | 99 | 401 | 736 |
|  |  | Six-hairpin glycosidases | ALHF_11738.g6590 | 335 | 1542 | 1602 |
|  | Coenzyme m/tr | Activating enzymes of the ubiquitin-like proteins | ALHF_01757.g984 | 250 | 927 | 848 |
|  |  |  | ALHF_02385.g1384 | 289 | 1488 | 3590 |
|  |  |  | ALHF_03772.g2387 | 4613 | 16340 | 19126 |
|  |  |  | ALHF_06858.g4497 | 179 | 734 | 993 |
|  |  | Acyl-CoA dehydrogenase NM domain-like | ALHF_01220.g669 | 299 | 1499 | 1713 |
|  |  | Class II aaRS and biotin synthetases | ALHF_02577.g1507 | 524 | 1932 | 1508 |
|  |  |  | ALHF_02813.g1668 | 546 | 4715 | 6589 |
|  |  |  | ALHF_02827.g1681 | 674 | 4014 | 3815 |
|  |  |  | ALHF_03213.g1968 | 1011 | 4278 | 4078 |
|  |  |  | ALHF_03967.g2548 | 1078 | 4689 | 4678 |
|  |  |  | ALHF_04377.g2880 | 960 | 4116 | 4176 |
|  |  |  | ALHF_04777.g3221 | 753 | 2637 | 2611 |
|  |  |  | ALHF_04995.g3394 | 1336 | 5132 | 6005 |
|  |  |  | ALHF_06527.g4316 | 364 | 2224 | 2426 |
|  |  | Glutathione synthetase ATP-binding domain-like | ALHF_04316.g2838 | 2101 | 13716 | 12284 |
|  |  |  | ALHF_08071.g5118 | 157 | 1215 | 1350 |
|  | E- transfer | Molybdenum cofactor-binding domain | ALHF_03745.g2363 | 313 | 2188 | 932 |
|  |  | Oxidoreductase molybdopterin-binding domain | ALHF_00120.g65 | 99 | 391 | 370 |
|  | Lipid m/tr | Acyl-CoA binding protein | ALHF_01771.g994 | 228 | 754 | 815 |
|  |  | YWTD domain | ALHF_00103.g59 | 169 | 17590 | 19279 |
|  |  |  | ALHF_08971.g5492 | 175 | 1308 | 178 |
|  |  |  | ALHF_11813.g6639 | 175 | 1308 | 178 |
|  | Nucleotide m/tr | Adenine nucleotide alpha hydrolases-like | ALHF_07444.g4804 | 284 | 1514 | 1932 |
|  |  | Nucleotidyltransferase | ALHF_01606.g889 | 372 | 1429 | 2242 |
|  |  | Nucleotidylyl transferase | ALHF_01099.g608 | 1265 | 4762 | 3527 |
|  |  |  | ALHF_02006.g1135 | 548 | 2512 | 2659 |
|  |  |  | ALHF_02028.g1149 | 1349 | 6796 | 4860 |
|  |  |  | ALHF_02121.g1211 | 434 | 1546 | 1911 |
|  |  |  | ALHF_03173.g1938 | 1809 | 7282 | 8284 |
|  |  |  | ALHF_03797.g2403 | 1023 | 4989 | 5472 |
|  |  |  | ALHF_04737.g3183 | 1130 | 9594 | 7988 |
|  |  |  | ALHF_04799.g3239 | 589 | 3398 | 2050 |
|  |  |  | ALHF_05831.g3962 | 520 | 2312 | 2613 |
|  |  |  | ALHF_10733.g5984 | 118 | 419 | 475 |
|  |  |  | ALHF_11312.g6296 | 521 | 2174 | 1911 |
|  |  | PRTase-like | ALHF_02237.g1291 | 142 | 608 | 754 |
|  |  |  | ALHF_04939.g3359 | 364 | 1509 | 2105 |
|  |  |  | ALHF_11796.g6628 | 173 | 671 | 709 |
|  |  | Pseudouridine synthase | ALHF_04393.g2895 | 457 | 4673 | 7649 |
|  |  |  | ALHF_09967.g5744 | 136 | 478 | 697 |
|  |  |  | ALHF_10256.g5839 | 136 | 478 | 697 |
|  |  | Purine and uridine phosphorylases | ALHF_01753.g983 | 198 | 822 | 975 |
|  |  | Ribonuclease H-like | ALHF_00666.g372 | 253 | 1812 | 3177 |
|  |  |  | ALHF_00968.g533 | 239 | 977 | 1280 |
|  |  |  | ALHF_01385.g758 | 207 | 2232 | 3450 |
|  |  |  | ALHF_03085.g1879 | 880 | 5480 | 12704 |
|  |  |  | ALHF_04441.g2935 | 724 | 4050 | 6244 |
|  |  |  | ALHF_06582.g4342 | 336 | 2284 | 3741 |
|  |  |  | ALHF_06601.g4353 | 301 | 1314 | 2986 |
|  |  |  | ALHF_07571.g4865 | 283 | 1031 | 3121 |
|  |  |  | ALHF_08029.g5095 | 2049 | 7995 | 9641 |
|  |  | Ribulose-phoshate binding barrel | ALHF_02960.g1779 | 766 | 3737 | 3449 |
|  |  |  | ALHF_05797.g3944 | 207 | 821 | 893 |
|  |  | SAICAR synthase-like | ALHF_02406.g1395 | 268 | 1267 | 1766 |
|  |  |  | ALHF_04583.g3058 | 762 | 2532 | 2630 |
|  |  | Staphylococcal nuclease | ALHF_03138.g1912 | 3313 | 18900 | 17589 |
|  | Other enzymes | Acetyl-CoA synthetase-like | ALHF_01797.g1008 | 195 | 803 | 1343 |
|  |  |  | ALHF_02262.g1314 | 238 | 846 | 1416 |
|  |  |  | ALHF_02823.g1677 | 674 | 2206 | 3720 |
|  |  |  | ALHF_03842.g2439 | 762 | 2803 | 5494 |
|  |  |  | ALHF_05403.g3710 | 1473 | 6352 | 1545 |
|  |  |  | ALHF_07392.g4774 | 152 | 992 | 1240 |
|  |  |  | ALHF_07512.g4837 | 142 | 586 | 1474 |
|  |  |  | ALHF_08224.g5183 | 54 | 231 | 465 |
|  |  |  | ALHF_08243.g5193 | 182 | 773 | 1199 |
|  |  |  | ALHF_08340.g5239 | 405 | 2369 | 5065 |
|  |  | Actin-like ATPase domain | ALHF_01346.g734 | 2334 | 7974 | 7554 |
|  |  |  | ALHF_03000.g1811 | 1152 | 4171 | 4059 |
|  |  |  | ALHF_03728.g2348 | 270 | 2316 | 3040 |
|  |  |  | ALHF_04021.g2586 | 942 | 1098 | 5031 |
|  |  |  | ALHF_04548.g3031 | 333 | 1105 | 1256 |
|  |  |  | ALHF_04549.g3032 | 1867 | 7680 | 7931 |
|  |  |  | ALHF_07784.g4970 | 127 | 679 | 1518 |
|  |  | Activator of Hsp90 ATPase, Aha1 | ALHF_00219.g106 | 425 | 1609 | 3468 |
|  |  | alpha/beta-Hydrolases | ALHF_03084.g1878 | 437 | 1696 | 2654 |
|  |  |  | ALHF_04154.g2697 | 5772 | 183408 | 58816 |
|  |  |  | ALHF_04443.g2937 | 1184 | 5437 | 3714 |
|  |  |  | ALHF_04779.g3223 | 631 | 149852 | 66355 |
|  |  |  | ALHF_06894.g4516 | 332 | 1940 | 1978 |
|  |  |  | ALHF_07152.g4653 | 275 | 1274 | 1473 |
|  |  |  | ALHF_07311.g4730 | 501 | 1770 | 2690 |
|  |  |  | ALHF_07720.g4935 | 205 | 867 | 966 |
|  |  |  | ALHF_08414.g5269 | 69 | 1045 | 1892 |
|  |  |  | ALHF_10698.g5968 | 86 | 341 | 325 |
|  |  |  | ALHF_10890.g6061 | 22 | 246 | 198 |
|  |  |  | ALHF_11416.g6368 | 0 | 15 | 90 |
|  |  |  | ALHF_11417.g6369 | 0 | 15 | 90 |
|  |  | Calcium-dependent phosphotriesterase | ALHF_10288.g5851 | 110 | 534 | 323 |
|  |  |  | ALHF_10354.g5864 | 110 | 534 | 323 |
|  |  | DHS-like NAD/FAD-binding domain | ALHF_05985.g4046 | 674 | 2649 | 4367 |
|  |  | HAD-like | ALHF_00361.g184 | 251 | 1351 | 1522 |
|  |  |  | ALHF_06461.g4280 | 132 | 954 | 791 |
|  |  |  | ALHF_08163.g5157 | 124 | 584 | 874 |
|  |  |  | ALHF_12683.g6780 | 36 | 269 | 329 |
|  |  | HD-domain/PDEase-like | ALHF_00298.g146 | 200 | 919 | 1587 |
|  |  | Indolic compounds 2,3-dioxygenase-like | ALHF_11495.g6426 | 2847 | 14159 | 2874 |
|  |  | LysM domain | ALHF_04328.g2849 | 532 | 4135 | 10512 |
|  |  | PFL-like glycyl radical enzymes | ALHF_03316.g2043 | 737 | 5357 | 11607 |
|  |  | Phosphoglycerate mutase-like | ALHF_10274.g5845 | 169 | 861 | 1903 |
|  |  | PurM C-terminal domain-like | ALHF_00242.g114 | 960 | 3669 | 5244 |
|  |  | Quinoprotein alcohol dehydrogenase-like | ALHF_01391.g761 | 254 | 1470 | 2725 |
|  |  | Radical SAM enzymes | ALHF_03220.g1973 | 246 | 1071 | 1372 |
|  |  | Ribokinase-like | ALHF_01015.g560 | 254 | 1252 | 1838 |
|  |  |  | ALHF_00410.g215 | 122 | 530 | 369 |
|  |  |  | ALHF_09998.g5757 | 224 | 860 | 644 |
|  |  |  | ALHF_10221.g5830 | 122 | 530 | 369 |
|  |  | SGNH hydrolase | ALHF_05551.g3807 | 637 | 13920 | 11387 |
|  |  |  | ALHF_06892.g4514 | 113 | 516 | 863 |
|  | Polysaccharide m/tr | Starch-binding domain-like | ALHF_04571.g3050 | 2006 | 6643 | 6588 |
|  |  | UDP-Glycosyltransferase/glycogen phosphorylase | ALHF_00948.g525 | 126 | 444 | 445 |
|  |  |  | ALHF_05259.g3603 | 46 | 182 | 280 |
|  | Redox | ALDH-like | ALHF_01401.g767 | 170 | 2939 | 647 |
|  |  |  | ALHF_06419.g4261 | 228 | 2709 | 1651 |
|  |  | Cytochrome P450 | ALHF_02049.g1162 | 5 | 738 | 186 |
|  |  |  | ALHF_08783.g5425 | 39 | 283 | 124 |
|  |  |  | ALHF_04704.g3157 | 754 | 5732 | 2756 |
|  |  | FAD/NAD-linked reductases, dimerisation (C-terminal) domain | ALHF_01204.g659 | 302 | 1983 | 2013 |
|  |  | Formate/glycerate dehydrogenase catalytic domain-like | ALHF_02893.g1728 | 114 | 487 | 1165 |
|  |  | GST C-terminal domain-like | ALHF_03573.g2227 | 311 | 1625 | 2600 |
|  |  | Inosine monophosphate dehydrogenase (IMPDH) | ALHF_02243.g1296 | 1782 | 8397 | 5952 |
|  |  | LDH C-terminal domain-like | ALHF_00676.g375 | 5838 | 7095 | 6713 |
|  |  | Metallo-hydrolase/oxidoreductase | ALHF_07405.g4780 | 242 | 1163 | 1338 |
|  |  |  | ALHF_08493.g5306 | 69 | 398 | 491 |
|  |  | Thioredoxin-like | ALHF_00264.g125 | 345 | 1400 | 1763 |
|  |  |  | ALHF_00305.g150 | 36 | 237 | 254 |
|  |  |  | ALHF_01870.g1054 | 108 | 477 | 615 |
|  |  |  | ALHF_03207.g1963 | 601 | 2101 | 2953 |
|  |  |  | ALHF_03872.g2464 | 320 | 1408 | 1527 |
|  |  |  | ALHF_04337.g2856 | 772 | 3200 | 2274 |
|  |  |  | ALHF_05322.g3654 | 3112 | 13259 | 11467 |
|  |  |  | ALHF_05815.g3953 | 308 | 3727 | 7486 |
|  |  |  | ALHF_07410.g4785 | 79 | 325 | 446 |
|  |  |  | ALHF_09995.g5755 | 114 | 398 | 430 |
|  |  |  | ALHF_10625.g5948 | 114 | 398 | 430 |
|  |  |  | ALHF_11922.g6699 | 308 | 3727 | 7486 |
|  | Secondary metabolism | ADP-ribosylation | ALHF_00830.g456 | 381 | 2052 | 5953 |
|  |  | Clavaminate synthase-like | ALHF_01646.g919 | 131 | 811 | 1812 |
|  |  |  | ALHF_07054.g4601 | 204 | 730 | 1438 |
|  |  |  | ALHF_08217.g5179 | 102 | 390 | 662 |
|  |  | Concanavalin A-like lectins/glucanases | ALHF_01442.g791 | 107 | 536 | 806 |
|  |  |  | ALHF_03962.g2545 | 580 | 2127 | 1727 |
|  |  |  | ALHF_05282.g3622 | 2640 | 9216 | 8366 |
|  |  |  | ALHF_06003.g4055 | 1516 | 1915 | 4489 |
|  |  |  | ALHF_06471.g4286 | 155 | 546 | 941 |
|  |  |  | ALHF_09457.g5650 | 110 | 423 | 767 |
|  |  | PR-1-like | ALHF_00904.g501 | 141 | 25320 | 4022 |
|  |  | Terpenoid cyclases/Protein prenyltransferases | ALHF_01819.g1022 | 4379 | 19168 | 5028 |
|  |  |  | ALHF_04060.g2619 | 229 | 1661 | 703 |
|  |  |  | ALHF_03739.g2356 | 1360 | 7348 | 1560 |
|  |  | Terpenoid synthases | ALHF_03549.g2207 | 166 | 653 | 929 |
|  | Transferases | Acyl-CoA N-acyltransferases (Nat) | ALHF_02256.g1308 | 150 | 961 | 1571 |
|  |  |  | ALHF_03117.g1901 | 247 | 932 | 1334 |
|  |  |  | ALHF_06806.g4465 | 247 | 1515 | 3318 |
|  |  |  | ALHF_07870.g5011 | 332 | 1987 | 3382 |
|  |  |  | ALHF_09244.g5587 | 64 | 377 | 526 |
|  |  | Class I glutamine amidotransferase-like | ALHF_11419.g6371 | 381 | 2679 | 2739 |
|  |  | Glycerol-3-phosphate (1)-acyltransferase | ALHF_00891.g493 | 418 | 1547 | 2139 |
|  |  |  | ALHF_05910.g4007 | 239 | 255 | 239 |
|  |  | NagB/RpiA/CoA transferase-like | ALHF_00707.g388 | 300 | 1946 | 3045 |
|  |  |  | ALHF_01321.g721 | 199 | 1366 | 1555 |
|  |  |  | ALHF_01517.g832 | 161 | 589 | 846 |
|  |  |  | ALHF_02505.g1461 | 185 | 672 | 863 |
|  |  | Nucleotide-diphospho-sugar transferases | ALHF_00187.g94 | 178 | 942 | 1393 |
|  |  |  | ALHF_01388.g759 | 429 | 1798 | 2041 |
|  |  |  | ALHF_07185.g4673 | 97 | 407 | 280 |
|  |  | PLP-dependent transferases | ALHF_00615.g343 | 177 | 973 | 2269 |
|  |  |  | ALHF_06638.g4372 | 126 | 586 | 966 |
|  |  | Protein prenylyltransferase | ALHF_02566.g1502 | 225 | 876 | 1108 |
|  |  | S-adenosyl-L-methionine-dependent methyltransferases | ALHF_00004.g3 | 167 | 1199 | 2851 |
|  |  |  | ALHF_00445.g235 | 265 | 1254 | 1250 |
|  |  |  | ALHF_00511.g281 | 351 | 2172 | 4475 |
|  |  |  | ALHF_00735.g400 | 195 | 1284 | 2135 |
|  |  |  | ALHF_01392.g762 | 289 | 3249 | 4928 |
|  |  |  | ALHF_02623.g1533 | 405 | 2077 | 2653 |
|  |  |  | ALHF_03397.g2103 | 266 | 2087 | 3008 |
|  |  |  | ALHF_03929.g2514 | 1126 | 1253 | 1127 |
|  |  |  | ALHF_06281.g4187 | 245 | 2133 | 2718 |
|  |  |  | ALHF_06849.g4493 | 134 | 465 | 768 |
|  |  |  | ALHF_06978.g4558 | 134 | 556 | 625 |
|  |  |  | ALHF_07224.g4691 | 145 | 583 | 1193 |
|  |  |  | ALHF_07576.g4868 | 189 | 633 | 1674 |
|  |  |  | ALHF_08169.g5161 | 49 | 195 | 372 |
|  |  |  | ALHF_08461.g5298 | 130 | 761 | 1160 |
|  |  |  | ALHF_09198.g5570 | 44 | 215 | 635 |
|  |  |  | ALHF_03079.g1873 | 325 | 1099 | 1370 |
| Regulation | DNA-binding | ARID-like | ALHF_02720.g1594 | 852 | 3308 | 7483 |
|  |  |  | ALHF_08304.g5219 | 88 | 864 | 2171 |
|  |  |  | ALHF_10781.g6007 | 521 | 2440 | 5756 |
|  |  |  | ALHF_06919.g4525 | 512 | 2698 | 5484 |
|  |  | ATP-dependent DNA ligase DNA-binding domain | ALHF_01860.g1048 | 260 | 1859 | 2991 |
|  |  | beta-beta-alpha zinc fingers | ALHF_00743.g404 | 198 | 1498 | 1815 |
|  |  |  | ALHF_02138.g1225 | 278 | 1158 | 1980 |
|  |  |  | ALHF_03181.g1943 | 835 | 5519 | 9703 |
|  |  |  | ALHF_03372.g2084 | 684 | 3552 | 2730 |
|  |  |  | ALHF_04112.g2661 | 416 | 2698 | 4620 |
|  |  |  | ALHF_04620.g3087 | 183 | 1266 | 2350 |
|  |  |  | ALHF_05306.g3640 | 3629 | 23025 | 28949 |
|  |  |  | ALHF_05559.g3810 | 159 | 854 | 579 |
|  |  |  | ALHF_06568.g4336 | 215 | 958 | 1993 |
|  |  |  | ALHF_06623.g4365 | 77 | 300 | 490 |
|  |  |  | ALHF_06835.g4483 | 161 | 646 | 1805 |
|  |  |  | ALHF_07266.g4708 | 189 | 848 | 1652 |
|  |  |  | ALHF_07622.g4890 | 170 | 802 | 1571 |
|  |  |  | ALHF_08055.g5107 | 156 | 599 | 1378 |
|  |  |  | ALHF_08123.g5137 | 182 | 1034 | 2135 |
|  |  |  | ALHF_08526.g5322 | 117 | 784 | 1619 |
|  |  |  | ALHF_08594.g5353 | 146 | 865 | 980 |
|  |  |  | ALHF_09083.g5524 | 164 | 846 | 1180 |
|  |  |  | ALHF_09318.g5611 | 68 | 552 | 1589 |
|  |  |  | ALHF_09550.g5682 | 87 | 544 | 1162 |
|  |  |  | ALHF_12319.g6760 | 50 | 251 | 1464 |
|  |  |  | ALHF_00984.g542 | 507 | 2565 | 4952 |
|  |  |  | ALHF_06587.g4345 | 306 | 1014 | 2256 |
|  |  |  | ALHF_07581.g4871 | 70 | 327 | 1708 |
|  |  |  | ALHF_08707.g5401 | 99 | 1739 | 3613 |
|  |  |  | ALHF_10181.g5815 | 91 | 801 | 1445 |
|  |  |  | ALHF_10592.g5940 | 128 | 792 | 1778 |
|  |  | Bromodomain | ALHF_00075.g46 | 128 | 910 | 2170 |
|  |  |  | ALHF_01483.g814 | 188 | 1438 | 1915 |
|  |  |  | ALHF_02915.g1743 | 1637 | 7219 | 16274 |
|  |  |  | ALHF_04194.g2732 | 174 | 709 | 1553 |
|  |  |  | ALHF_06178.g4132 | 216 | 1192 | 1612 |
|  |  |  | ALHF_07885.g5016 | 118 | 706 | 819 |
|  |  |  | ALHF_08003.g5083 | 264 | 2152 | 5101 |
|  |  |  | ALHF_08207.g5175 | 419 | 2240 | 5612 |
|  |  |  | ALHF_08323.g5232 | 145 | 783 | 1608 |
|  |  |  | ALHF_08501.g5310 | 1180 | 6347 | 15906 |
|  |  | CCCH zinc finger | ALHF_02359.g1370 | 502 | 1778 | 3737 |
|  |  |  | ALHF_03078.g1872 | 587 | 1985 | 2760 |
|  |  |  | ALHF_06777.g4450 | 223 | 778 | 1371 |
|  |  | Conserved domain common to transcription factors TFIIS, elongin A, CRSP70 | ALHF_07453.g4810 | 185 | 1248 | 2166 |
|  |  | Core binding factor beta, CBF | ALHF_07913.g5035 | 72 | 353 | 1336 |
|  |  | Cyclin-like | ALHF_03450.g2136 | 190 | 677 | 1315 |
|  |  |  | ALHF_04147.g2692 | 246 | 26819 | 47680 |
|  |  |  | ALHF_06124.g4113 | 497 | 1816 | 3152 |
|  |  |  | ALHF_07135.g4647 | 99 | 396 | 783 |
|  |  |  | ALHF_07971.g5062 | 30 | 2138 | 4596 |
|  |  |  | ALHF_08435.g5281 | 33 | 324 | 507 |
|  |  | DNA-binding domain | ALHF_07566.g4863 | 311 | 1456 | 1819 |
|  |  | Glucocorticoid receptor-like (DNA-binding domain) | ALHF_06068.g4086 | 280 | 1911 | 3665 |
|  |  |  | ALHF_07523.g4841 | 120 | 632 | 1526 |
|  |  |  | ALHF_08859.g5450 | 145 | 703 | 654 |
|  |  |  | ALHF_09811.g5713 | 81 | 474 | 686 |
|  |  |  | ALHF_12344.g6761 | 82 | 353 | 805 |
|  |  |  | ALHF_09616.g5697 | 101 | 426 | 1060 |
|  |  | Histone-fold | ALHF_07270.g4711 | 95 | 509 | 610 |
|  |  |  | ALHF_07665.g4909 | 151 | 761 | 1207 |
|  |  | HLH, helix-loop-helix DNA-binding domain | ALHF_01995.g1127 | 51 | 266 | 166 |
|  |  |  | ALHF_03777.g2392 | 823 | 4711 | 6626 |
|  |  |  | ALHF_04155.g2698 | 577 | 2579 | 2382 |
|  |  | HMG-box | ALHF_00266.g127 | 44 | 3256 | 7601 |
|  |  |  | ALHF_06564.g4335 | 145 | 692 | 1645 |
|  |  |  | ALHF_07960.g5055 | 297 | 1538 | 3023 |
|  |  | Homeodomain-like | ALHF_03076.g1870 | 324 | 1143 | 1835 |
|  |  |  | ALHF_06736.g4426 | 582 | 3342 | 6864 |
|  |  |  | ALHF_07450.g4808 | 170 | 849 | 1838 |
|  |  |  | ALHF_07746.g4947 | 221 | 1048 | 1709 |
|  |  |  | ALHF_07238.g4694 | 805 | 4299 | 9279 |
|  |  | Insert subdomain of RNA polymerase alpha subunit | ALHF_07320.g4735 | 96 | 701 | 1238 |
|  |  |  | ALHF_08429.g5277 | 83 | 506 | 777 |
|  |  | Kix domain of CBP (creb binding protein) | ALHF_01283.g703 | 53 | 217 | 471 |
|  |  | p53-like transcription factors | ALHF_00913.g507 | 156 | 748 | 970 |
|  |  |  | ALHF_06269.g4178 | 1542 | 5178 | 7190 |
|  |  | PAH2 domain | ALHF_11699.g6562 | 2866 | 11136 | 16828 |
|  |  |  | ALHF_09993.g5753 | 349 | 1491 | 2633 |
|  |  | RPB6/omega subunit-like | ALHF_06167.g4126 | 92 | 415 | 693 |
|  |  | SAM/Pointed domain | ALHF_06438.g4270 | 183 | 695 | 1098 |
|  |  | SAP domain | ALHF_01500.g822 | 215 | 878 | 1397 |
|  |  |  | ALHF_06779.g4451 | 285 | 2161 | 4477 |
|  |  | SMAD/FHA domain | ALHF_08644.g5373 | 150 | 652 | 1027 |
|  |  | Transcription factor IIA (TFIIA), beta-barrel domain | ALHF_05560.g3811 | 792 | 2601 | 2654 |
|  |  | Winged helix DNA-binding domain | ALHF_04000.g2569 | 0 | 6010 | 720 |
|  |  |  | ALHF_04640.g3104 | 1292 | 5308 | 4781 |
|  |  |  | ALHF_04676.g3134 | 624 | 2050 | 2504 |
|  |  |  | ALHF_06580.g4341 | 329 | 1169 | 1839 |
|  |  |  | ALHF_11485.g6418 | 244 | 860 | 877 |
|  |  |  | ALHF_05344.g3669 | 0 | 9 | 12 |
|  | Kinases/phosphatases | (Phosphotyrosine protein) phosphatases II | ALHF_00159.g86 | 652 | 2913 | 5451 |
|  |  |  | ALHF_03744.g2362 | 1241 | 5550 | 9740 |
|  |  |  | ALHF_03413.g2114 | 156 | 607 | 1118 |
|  |  | PP2C-like | ALHF_02625.g1535 | 569 | 2508 | 4112 |
|  |  | Protein kinase-like (PK-like) | ALHF_01944.g1098 | 349 | 1177 | 1934 |
|  |  |  | ALHF_02551.g1492 | 159 | 992 | 1556 |
|  |  |  | ALHF_02941.g1763 | 530 | 2678 | 5146 |
|  |  |  | ALHF_03602.g2249 | 2198 | 7165 | 8581 |
|  |  |  | ALHF_04613.g3083 | 160 | 2018 | 1189 |
|  |  |  | ALHF_04615.g3085 | 681 | 3897 | 8030 |
|  |  |  | ALHF_05667.g3871 | 586 | 4749 | 1860 |
|  |  |  | ALHF_05689.g3884 | 515 | 1984 | 2524 |
|  |  |  | ALHF_06259.g4174 | 266 | 981 | 1232 |
|  |  |  | ALHF_06682.g4400 | 407 | 1692 | 2401 |
|  |  |  | ALHF_07367.g4757 | 156 | 584 | 854 |
|  |  |  | ALHF_08041.g5099 | 520 | 2351 | 4920 |
|  |  |  | ALHF_08655.g5377 | 2046 | 9297 | 10878 |
|  |  |  | ALHF_08924.g5474 | 65 | 629 | 3491 |
|  |  |  | ALHF_09100.g5529 | 318 | 1705 | 3246 |
|  |  |  | ALHF_09127.g5542 | 245 | 2309 | 3014 |
|  |  |  | ALHF_09317.g5610 | 173 | 884 | 803 |
|  |  |  | ALHF_09364.g5624 | 14 | 793 | 1270 |
|  |  |  | ALHF_09904.g5718 | 107 | 542 | 821 |
|  |  |  | ALHF_13288.g6805 | 35 | 444 | 686 |
|  |  |  | ALHF_00927.g516 | 141 | 642 | 1075 |
|  |  |  | ALHF_02173.g1244 | 403 | 1572 | 2816 |
|  |  |  | ALHF_07713.g4931 | 192 | 925 | 1137 |
|  |  |  | ALHF_09044.g5511 | 19 | 258 | 659 |
|  |  |  | ALHF_09275.g5597 | 52 | 1988 | 3454 |
|  |  |  | ALHF_10339.g5860 | 218 | 1582 | 2481 |
|  |  |  | ALHF_10756.g5995 | 263 | 1288 | 2776 |
|  |  |  | ALHF_11900.g6692 | 445 | 2149 | 2378 |
|  | Other regulatory function | ASF1-like | ALHF_00740.g402 | 51 | 202 | 673 |
|  |  |  | ALHF_12034.g6731 | 51 | 202 | 673 |
|  |  | Ran binding protein zinc finger-like | ALHF_07597.g4878 | 497 | 3308 | 4615 |
|  |  | Sec7 domain | ALHF_02803.g1660 | 431 | 1792 | 1751 |
|  | RNA binding, m/tr | NOB1 zinc finger-like | ALHF_02929.g1753 | 207 | 1062 | 1585 |
|  |  | Nop domain | ALHF_02751.g1620 | 766 | 4200 | 7171 |
|  |  |  | ALHF_07373.g4762 | 118 | 423 | 601 |
|  |  |  | ALHF_01296.g709 | 512 | 6774 | 12948 |
|  |  | PWI domain | ALHF_05052.g3441 | 965 | 4166 | 7032 |
|  |  | R3H domain | ALHF_02174.g1245 | 1279 | 5226 | 5681 |
|  |  |  | ALHF_02602.g1522 | 166 | 585 | 1112 |
|  |  |  | ALHF_07031.g4588 | 89 | 321 | 522 |
|  |  | RNA-binding domain, RBD | ALHF_00150.g81 | 221 | 819 | 1288 |
|  |  |  | ALHF_00321.g160 | 281 | 1144 | 2346 |
|  |  |  | ALHF_00422.g224 | 699 | 3578 | 4996 |
|  |  |  | ALHF_00479.g259 | 1715 | 6610 | 6625 |
|  |  |  | ALHF_01061.g585 | 186 | 1541 | 2679 |
|  |  |  | ALHF_01885.g1063 | 217 | 963 | 1471 |
|  |  |  | ALHF_02048.g1161 | 135 | 820 | 1662 |
|  |  |  | ALHF_02128.g1216 | 338 | 1122 | 2208 |
|  |  |  | ALHF_02156.g1232 | 286 | 1683 | 3484 |
|  |  |  | ALHF_02235.g1289 | 269 | 962 | 1526 |
|  |  |  | ALHF_02450.g1424 | 192 | 869 | 1093 |
|  |  |  | ALHF_02840.g1692 | 388 | 2447 | 4285 |
|  |  |  | ALHF_02848.g1699 | 784 | 6692 | 6764 |
|  |  |  | ALHF_03086.g1880 | 471 | 1908 | 3167 |
|  |  |  | ALHF_03087.g1881 | 595 | 3782 | 8877 |
|  |  |  | ALHF_03198.g1958 | 426 | 2310 | 5066 |
|  |  |  | ALHF_03700.g2327 | 88 | 603 | 957 |
|  |  |  | ALHF_03823.g2424 | 622 | 4393 | 8651 |
|  |  |  | ALHF_04202.g2736 | 311 | 3679 | 4095 |
|  |  |  | ALHF_04224.g2755 | 485 | 2365 | 2551 |
|  |  |  | ALHF_04512.g2997 | 931 | 5234 | 11574 |
|  |  |  | ALHF_04579.g3056 | 871 | 3644 | 5838 |
|  |  |  | ALHF_04808.g3247 | 953 | 4104 | 8167 |
|  |  |  | ALHF_04968.g3372 | 352 | 2200 | 3481 |
|  |  |  | ALHF_05529.g3791 | 316 | 1550 | 2364 |
|  |  |  | ALHF_06083.g4094 | 364 | 1491 | 2280 |
|  |  |  | ALHF_06142.g4119 | 198 | 728 | 1007 |
|  |  |  | ALHF_06204.g4148 | 766 | 2985 | 3619 |
|  |  |  | ALHF_06410.g4255 | 88 | 631 | 1033 |
|  |  |  | ALHF_06511.g4307 | 212 | 728 | 1210 |
|  |  |  | ALHF_06574.g4339 | 111 | 532 | 777 |
|  |  |  | ALHF_06764.g4442 | 115 | 725 | 1377 |
|  |  |  | ALHF_07005.g4571 | 233 | 923 | 1091 |
|  |  |  | ALHF_07030.g4587 | 151 | 552 | 902 |
|  |  |  | ALHF_07096.g4631 | 314 | 1151 | 1042 |
|  |  |  | ALHF_07102.g4633 | 144 | 516 | 965 |
|  |  |  | ALHF_07652.g4900 | 104 | 454 | 875 |
|  |  |  | ALHF_07842.g5000 | 113 | 800 | 1666 |
|  |  |  | ALHF_08181.g5165 | 58 | 641 | 1190 |
|  |  |  | ALHF_08634.g5369 | 56 | 231 | 521 |
|  |  |  | ALHF_09288.g5600 | 108 | 1337 | 3373 |
|  |  |  | ALHF_10405.g5877 | 86 | 300 | 437 |
|  |  |  | ALHF_10714.g5976 | 88 | 603 | 957 |
|  |  |  | ALHF_11058.g6142 | 566 | 3412 | 7198 |
|  |  |  | ALHF_11138.g6191 | 197 | 1892 | 1719 |
|  |  |  | ALHF_11145.g6195 | 136 | 1191 | 2073 |
|  |  |  | ALHF_11243.g6251 | 434 | 1453 | 1913 |
|  |  |  | ALHF_11320.g6301 | 88 | 603 | 957 |
|  |  |  | ALHF_11446.g6388 | 735 | 4085 | 5643 |
|  |  |  | ALHF_11462.g6399 | 735 | 4085 | 5643 |
|  |  |  | ALHF_12054.g6734 | 288 | 1711 | 3592 |
|  |  |  | ALHF_05630.g3849 | 336 | 1672 | 2984 |
|  |  |  | ALHF_06608.g4358 | 46 | 240 | 535 |
|  |  |  | ALHF_06796.g4458 | 262 | 1761 | 3356 |
|  |  |  | ALHF_11560.g6468 | 654 | 4674 | 2924 |
|  |  |  | ALHF_12526.g6768 | 51 | 215 | 306 |
|  |  | Surp module (SWAP domain) | ALHF_00276.g131 | 600 | 2203 | 3040 |
|  |  | TROVE domain-like | ALHF_08186.g5168 | 162 | 660 | 1126 |
|  | Signal transduction | C2 domain (Calcium/lipid-binding domain, CaLB) | ALHF_08827.g5440 | 61 | 222 | 358 |
|  |  | DBL homology domain (DH-domain) | ALHF_09133.g5545 | 113 | 525 | 674 |
|  |  |  | ALHF_08452.g5292 | 221 | 1152 | 1495 |
|  |  | Growth factor receptor domain | ALHF_03715.g2338 | 175 | 653 | 742 |
|  |  | GTPase activation domain, GAP | ALHF_03607.g2253 | 765 | 2751 | 4606 |
|  |  |  | ALHF_06557.g4332 | 730 | 2640 | 4599 |
|  |  |  | ALHF_08262.g5199 | 70 | 599 | 1355 |
|  |  | N-terminal domain of cbl (N-cbl) | ALHF_13820.g6824 | 17 | 135 | 387 |
|  |  | Nuclear receptor ligand-binding domain | ALHF_02162.g1237 | 298 | 1200 | 1659 |
|  |  | PDZ domain-like | ALHF_00842.g463 | 105 | 481 | 663 |
|  |  |  | ALHF_01058.g582 | 180 | 733 | 997 |
|  |  |  | ALHF_01361.g743 | 118 | 475 | 591 |
|  |  |  | ALHF_01743.g977 | 126 | 1078 | 132 |
|  |  |  | ALHF_02255.g1307 | 1565 | 8744 | 4672 |
|  |  |  | ALHF_02320.g1346 | 143 | 559 | 1160 |
|  |  |  | ALHF_04197.g2733 | 1842 | 6430 | 7623 |
|  |  |  | ALHF_05218.g3572 | 6981 | 23092 | 22739 |
|  |  |  | ALHF_05363.g3683 | 2188 | 8928 | 6860 |
|  |  |  | ALHF_05393.g3703 | 321 | 1231 | 923 |
|  |  |  | ALHF_06532.g4320 | 92 | 321 | 562 |
|  |  |  | ALHF_06833.g4482 | 382 | 1883 | 2821 |
|  |  |  | ALHF_06914.g4523 | 124 | 461 | 887 |
|  |  |  | ALHF_07306.g4729 | 344 | 1897 | 2307 |
|  |  |  | ALHF_07837.g4996 | 31 | 122 | 110 |
|  |  |  | ALHF_08667.g5384 | 490 | 2055 | 3064 |
|  |  |  | ALHF_09097.g5528 | 366 | 1305 | 2816 |
|  |  |  | ALHF_11638.g6528 | 200 | 3520 | 5476 |
|  |  | PH domain-like | ALHF_00063.g37 | 203 | 1157 | 2236 |
|  |  |  | ALHF_00200.g101 | 261 | 990 | 1855 |
|  |  |  | ALHF_00208.g102 | 456 | 2709 | 2695 |
|  |  |  | ALHF_00516.g285 | 245 | 952 | 1578 |
|  |  |  | ALHF_03135.g1909 | 1310 | 9012 | 15077 |
|  |  |  | ALHF_03788.g2396 | 457 | 1755 | 1894 |
|  |  |  | ALHF_04652.g3113 | 935 | 5335 | 6315 |
|  |  |  | ALHF_04688.g3144 | 1359 | 4442 | 4270 |
|  |  |  | ALHF_07782.g4969 | 144 | 503 | 846 |
|  |  |  | ALHF_09258.g5591 | 54 | 1200 | 2158 |
|  |  |  | ALHF_12160.g6747 | 138 | 574 | 797 |
|  |  |  | ALHF_12212.g6754 | 138 | 574 | 797 |
|  |  |  | ALHF_07664.g4908 | 89 | 406 | 587 |
|  |  | PX domain | ALHF_01543.g848 | 187 | 757 | 1224 |
|  |  | Rap/Ran-GAP | ALHF_10942.g6080 | 308 | 1629 | 1867 |
|  |  | Ras GEF | ALHF_09282.g5598 | 82 | 377 | 746 |
|  |  | Regulator of G-protein signaling, RGS | ALHF_08510.g5315 | 85 | 350 | 650 |
|  |  | RUN domain-like | ALHF_08736.g5407 | 504 | 1775 | 2709 |
|  |  | SH2 domain | ALHF_01599.g885 | 216 | 849 | 1032 |
|  |  |  | ALHF_02179.g1248 | 118 | 427 | 661 |
|  |  |  | ALHF_06651.g4380 | 163 | 546 | 830 |
|  |  |  | ALHF_07038.g4592 | 217 | 1395 | 2176 |
|  |  |  | ALHF_08254.g5196 | 31 | 146 | 243 |
|  |  | SH3-domain | ALHF_00359.g183 | 365 | 1210 | 2046 |
|  |  |  | ALHF_01309.g715 | 532 | 1830 | 2320 |
|  |  |  | ALHF_03022.g1829 | 690 | 3092 | 3494 |
|  |  |  | ALHF_03677.g2307 | 320 | 1246 | 1722 |
|  |  |  | ALHF_08603.g5357 | 259 | 2139 | 3275 |
|  |  | Stathmin | ALHF_03108.g1895 | 67 | 581 | 1290 |
|  |  | Toll/Interleukin receptor TIR domain | ALHF_11150.g6200 | 42 | 292 | 525 |
|  |  | Ypt/Rab-GAP domain of gyp1p | ALHF_00683.g379 | 58 | 405 | 665 |
|  |  |  | ALHF_06893.g4515 | 192 | 988 | 1542 |
|  |  |  | ALHF_08422.g5273 | 111 | 425 | 809 |
| Other | Unknown function | alpha/beta knot | ALHF_07977.g5067 | 134 | 1372 | 891 |
|  |  | beta-sandwich domain of Sec23/24 | ALHF_07286.g4719 | 115 | 581 | 1267 |
|  |  |  | ALHF_08018.g5090 | 76 | 362 | 639 |
|  |  | DEK C-terminal domain | ALHF_04288.g2810 | 528 | 2222 | 2744 |
|  |  | E set domains | ALHF_06382.g4241 | 84 | 365 | 478 |
|  |  |  | ALHF_10968.g6091 | 223 | 1269 | 1611 |
|  |  |  | ALHF_12008.g6724 | 151 | 589 | 804 |
|  |  |  | ALHF_12087.g6742 | 151 | 589 | 804 |
|  |  | FF domain | ALHF_06435.g4268 | 256 | 1132 | 1817 |
|  |  | Gamma-glutamyl cyclotransferase-like | ALHF_02940.g1762 | 391 | 3240 | 2944 |
|  |  | HCP-like | ALHF_01613.g895 | 269 | 977 | 1414 |
|  |  | Hook domain | ALHF_07881.g5015 | 311 | 1276 | 2399 |
|  |  | Hypothetical protein PH1602 | ALHF_03987.g2560 | 146 | 634 | 521 |
|  |  |  | ALHF_11627.g6517 | 40 | 230 | 289 |
|  |  | PIN domain-like | ALHF_08090.g5127 | 73 | 811 | 1461 |
|  |  | Prim-pol domain | ALHF_00778.g425 | 96 | 365 | 1112 |
|  |  | SpoIIaa-like | ALHF_02114.g1207 | 411 | 2143 | 1925 |
|  |  | YebC-like | ALHF_12592.g6772 | 41 | 231 | 506 |
|  | Viral proteins | Retrovirus zinc finger-like domains | ALHF_00162.g88 | 286 | 2133 | 1371 |
|  |  |  | ALHF_05267.g3610 | 1317 | 4298 | 4896 |
|  |  |  | ALHF_09154.g5555 | 41 | 170 | 360 |
|  |  |  | ALHF_08233.g5188 | 167 | 1229 | 844 |
| NONA | not annotated | NONA | ALHF_00029.g22 | 188 | 712 | 1475 |
|  |  |  | ALHF_00031.g24 | 176 | 801 | 1623 |
|  |  |  | ALHF_00080.g49 | 120 | 411 | 504 |
|  |  |  | ALHF_00096.g57 | 84 | 521 | 823 |
|  |  |  | ALHF_00143.g76 | 96 | 342 | 363 |
|  |  |  | ALHF_00221.g107 | 720 | 2998 | 2535 |
|  |  |  | ALHF_00354.g179 | 405 | 1916 | 3801 |
|  |  |  | ALHF_00446.g236 | 270 | 2003 | 3731 |
|  |  |  | ALHF_00451.g239 | 363 | 4687 | 1068 |
|  |  |  | ALHF_00474.g255 | 991 | 6449 | 9900 |
|  |  |  | ALHF_00548.g308 | 146 | 581 | 1154 |
|  |  |  | ALHF_00610.g340 | 478 | 1684 | 2461 |
|  |  |  | ALHF_00624.g346 | 100 | 433 | 1300 |
|  |  |  | ALHF_00625.g347 | 150 | 523 | 485 |
|  |  |  | ALHF_00649.g362 | 68 | 515 | 792 |
|  |  |  | ALHF_00657.g367 | 242 | 1430 | 2142 |
|  |  |  | ALHF_00730.g397 | 66 | 394 | 512 |
|  |  |  | ALHF_00732.g398 | 564 | 2721 | 6548 |
|  |  |  | ALHF_00796.g436 | 160 | 1664 | 2552 |
|  |  |  | ALHF_00861.g474 | 132 | 509 | 821 |
|  |  |  | ALHF_00885.g488 | 614 | 2113 | 2011 |
|  |  |  | ALHF_00905.g502 | 365 | 1975 | 3982 |
|  |  |  | ALHF_00921.g512 | 63 | 240 | 470 |
|  |  |  | ALHF_00932.g517 | 27 | 134 | 271 |
|  |  |  | ALHF_00958.g530 | 85 | 97 | 277 |
|  |  |  | ALHF_01022.g563 | 1032 | 1182 | 4681 |
|  |  |  | ALHF_01026.g566 | 177 | 998 | 1458 |
|  |  |  | ALHF_01098.g607 | 155 | 575 | 1375 |
|  |  |  | ALHF_01138.g625 | 192 | 780 | 1530 |
|  |  |  | ALHF_01152.g631 | 510 | 1655 | 2335 |
|  |  |  | ALHF_01161.g635 | 520 | 2585 | 1458 |
|  |  |  | ALHF_01168.g639 | 872 | 2953 | 7002 |
|  |  |  | ALHF_01174.g642 | 162 | 623 | 807 |
|  |  |  | ALHF_01201.g658 | 139 | 722 | 939 |
|  |  |  | ALHF_01223.g670 | 151 | 569 | 936 |
|  |  |  | ALHF_01224.g671 | 190 | 1315 | 1917 |
|  |  |  | ALHF_01264.g689 | 187 | 649 | 1530 |
|  |  |  | ALHF_01313.g717 | 74 | 263 | 287 |
|  |  |  | ALHF_01329.g725 | 141 | 918 | 1495 |
|  |  |  | ALHF_01337.g729 | 172 | 636 | 1088 |
|  |  |  | ALHF_01345.g733 | 238 | 909 | 1152 |
|  |  |  | ALHF_01353.g739 | 1057 | 3974 | 2965 |
|  |  |  | ALHF_01366.g745 | 578 | 2709 | 2932 |
|  |  |  | ALHF_01375.g752 | 98 | 620 | 1134 |
|  |  |  | ALHF_01399.g766 | 310 | 1327 | 1207 |
|  |  |  | ALHF_01403.g768 | 1274 | 9728 | 20383 |
|  |  |  | ALHF_01422.g777 | 598 | 3756 | 6429 |
|  |  |  | ALHF_01439.g789 | 571 | 2441 | 5740 |
|  |  |  | ALHF_01453.g794 | 620 | 668 | 676 |
|  |  |  | ALHF_01504.g825 | 365 | 1434 | 3177 |
|  |  |  | ALHF_01513.g829 | 90 | 1650 | 2565 |
|  |  |  | ALHF_01525.g836 | 290 | 1022 | 1944 |
|  |  |  | ALHF_01553.g855 | 94 | 593 | 1138 |
|  |  |  | ALHF_01576.g870 | 371 | 2815 | 3310 |
|  |  |  | ALHF_01583.g875 | 194 | 961 | 1195 |
|  |  |  | ALHF_01612.g894 | 415 | 1690 | 2228 |
|  |  |  | ALHF_01639.g914 | 219 | 734 | 1359 |
|  |  |  | ALHF_01677.g941 | 118 | 758 | 1432 |
|  |  |  | ALHF_01696.g953 | 153 | 571 | 601 |
|  |  |  | ALHF_01813.g1017 | 621 | 2106 | 2865 |
|  |  |  | ALHF_01846.g1041 | 315 | 1346 | 1333 |
|  |  |  | ALHF_01881.g1062 | 420 | 1372 | 2401 |
|  |  |  | ALHF_01889.g1066 | 408 | 1475 | 1981 |
|  |  |  | ALHF_01916.g1082 | 703 | 829 | 738 |
|  |  |  | ALHF_01920.g1085 | 1423 | 4619 | 7065 |
|  |  |  | ALHF_01923.g1087 | 186 | 1048 | 1406 |
|  |  |  | ALHF_01951.g1102 | 181 | 982 | 861 |
|  |  |  | ALHF_01972.g1112 | 346 | 1217 | 1967 |
|  |  |  | ALHF_01974.g1113 | 614 | 15239 | 20553 |
|  |  |  | ALHF_01988.g1122 | 314 | 1109 | 2340 |
|  |  |  | ALHF_01994.g1126 | 228 | 996 | 2217 |
|  |  |  | ALHF_02070.g1177 | 291 | 353 | 828 |
|  |  |  | ALHF_02078.g1182 | 87 | 650 | 1326 |
|  |  |  | ALHF_02083.g1185 | 112 | 596 | 903 |
|  |  |  | ALHF_02084.g1186 | 537 | 1797 | 2587 |
|  |  |  | ALHF_02101.g1198 | 1058 | 4206 | 6937 |
|  |  |  | ALHF_02123.g1212 | 357 | 2302 | 3628 |
|  |  |  | ALHF_02161.g1236 | 315 | 1593 | 2285 |
|  |  |  | ALHF_02190.g1256 | 570 | 7048 | 3374 |
|  |  |  | ALHF_02213.g1270 | 389 | 2338 | 4594 |
|  |  |  | ALHF_02231.g1286 | 51 | 7495 | 14593 |
|  |  |  | ALHF_02244.g1297 | 150 | 529 | 1064 |
|  |  |  | ALHF_02248.g1301 | 231 | 811 | 820 |
|  |  |  | ALHF_02257.g1309 | 303 | 1338 | 1432 |
|  |  |  | ALHF_02288.g1328 | 222 | 902 | 4113 |
|  |  |  | ALHF_02328.g1351 | 242 | 980 | 777 |
|  |  |  | ALHF_02376.g1381 | 349 | 1598 | 1564 |
|  |  |  | ALHF_02411.g1399 | 1400 | 5120 | 11373 |
|  |  |  | ALHF_02422.g1406 | 430 | 1536 | 3207 |
|  |  |  | ALHF_02433.g1411 | 1199 | 4215 | 3716 |
|  |  |  | ALHF_02493.g1451 | 2215 | 8528 | 10049 |
|  |  |  | ALHF_02524.g1471 | 352 | 1163 | 1351 |
|  |  |  | ALHF_02530.g1474 | 336 | 1218 | 1433 |
|  |  |  | ALHF_02533.g1477 | 415 | 2788 | 3122 |
|  |  |  | ALHF_02549.g1490 | 198 | 761 | 757 |
|  |  |  | ALHF_02599.g1520 | 454 | 2709 | 2679 |
|  |  |  | ALHF_02603.g1523 | 548 | 1802 | 2052 |
|  |  |  | ALHF_02615.g1530 | 2223 | 7892 | 10035 |
|  |  |  | ALHF_02627.g1537 | 157 | 548 | 850 |
|  |  |  | ALHF_02648.g1548 | 472 | 1758 | 4831 |
|  |  |  | ALHF_02690.g1569 | 395 | 1771 | 3358 |
|  |  |  | ALHF_02707.g1582 | 339 | 3421 | 5134 |
|  |  |  | ALHF_02735.g1607 | 234 | 1162 | 2471 |
|  |  |  | ALHF_02787.g1647 | 797 | 3426 | 6764 |
|  |  |  | ALHF_02788.g1648 | 348 | 1901 | 2793 |
|  |  |  | ALHF_02814.g1669 | 455 | 2173 | 5220 |
|  |  |  | ALHF_02815.g1670 | 409 | 1766 | 1802 |
|  |  |  | ALHF_02817.g1672 | 755 | 4796 | 7453 |
|  |  |  | ALHF_02842.g1693 | 457 | 3408 | 3819 |
|  |  |  | ALHF_02860.g1709 | 569 | 3316 | 5287 |
|  |  |  | ALHF_02909.g1738 | 1006 | 1197 | 1048 |
|  |  |  | ALHF_03146.g1919 | 79 | 277 | 448 |
|  |  |  | ALHF_03193.g1953 | 0 | 64 | 98 |
|  |  |  | ALHF_03221.g1974 | 443 | 1779 | 2644 |
|  |  |  | ALHF_03229.g1981 | 147 | 690 | 1208 |
|  |  |  | ALHF_03323.g2048 | 967 | 12721 | 18784 |
|  |  |  | ALHF_03330.g2054 | 891 | 4726 | 7221 |
|  |  |  | ALHF_03414.g2115 | 654 | 6416 | 8010 |
|  |  |  | ALHF_03419.g2119 | 640 | 2908 | 1946 |
|  |  |  | ALHF_03443.g2132 | 1020 | 5284 | 6514 |
|  |  |  | ALHF_03457.g2142 | 245 | 1146 | 2747 |
|  |  |  | ALHF_03538.g2198 | 915 | 4139 | 7025 |
|  |  |  | ALHF_03572.g2226 | 213 | 795 | 1115 |
|  |  |  | ALHF_03583.g2235 | 44 | 235 | 302 |
|  |  |  | ALHF_03594.g2244 | 513 | 1687 | 2867 |
|  |  |  | ALHF_03632.g2273 | 1027 | 6021 | 13110 |
|  |  |  | ALHF_03660.g2296 | 902 | 2968 | 4711 |
|  |  |  | ALHF_03696.g2323 | 208 | 949 | 810 |
|  |  |  | ALHF_03699.g2326 | 421 | 1608 | 1178 |
|  |  |  | ALHF_03736.g2353 | 342 | 1239 | 1323 |
|  |  |  | ALHF_03806.g2411 | 644 | 2187 | 2396 |
|  |  |  | ALHF_03902.g2492 | 698 | 8588 | 11891 |
|  |  |  | ALHF_03903.g2493 | 429 | 3552 | 5001 |
|  |  |  | ALHF_03920.g2506 | 197 | 673 | 1526 |
|  |  |  | ALHF_03926.g2511 | 874 | 1062 | 933 |
|  |  |  | ALHF_03930.g2515 | 118 | 410 | 633 |
|  |  |  | ALHF_03950.g2534 | 607 | 4289 | 5406 |
|  |  |  | ALHF_04017.g2582 | 48 | 326 | 637 |
|  |  |  | ALHF_04080.g2635 | 1140 | 3997 | 3902 |
|  |  |  | ALHF_04130.g2676 | 804 | 2654 | 2859 |
|  |  |  | ALHF_04158.g2701 | 120 | 516 | 894 |
|  |  |  | ALHF_04263.g2789 | 471 | 1871 | 4258 |
|  |  |  | ALHF_04310.g2832 | 416 | 1809 | 2207 |
|  |  |  | ALHF_04325.g2846 | 680 | 2557 | 2454 |
|  |  |  | ALHF_04348.g2864 | 204 | 815 | 665 |
|  |  |  | ALHF_04356.g2869 | 896 | 2966 | 3369 |
|  |  |  | ALHF_04389.g2890 | 0 | 1181 | 1070 |
|  |  |  | ALHF_04428.g2923 | 488 | 2508 | 1927 |
|  |  |  | ALHF_04513.g2998 | 358 | 2693 | 4213 |
|  |  |  | ALHF_04527.g3010 | 73 | 274 | 302 |
|  |  |  | ALHF_04545.g3028 | 1040 | 3980 | 3783 |
|  |  |  | ALHF_04600.g3072 | 784 | 26983 | 11402 |
|  |  |  | ALHF_04651.g3111 | 706 | 2330 | 2287 |
|  |  |  | ALHF_04724.g3170 | 1550 | 5763 | 4533 |
|  |  |  | ALHF_04746.g3191 | 105 | 567 | 734 |
|  |  |  | ALHF_04778.g3222 | 670 | 2656 | 4234 |
|  |  |  | ALHF_04782.g3225 | 823 | 2720 | 4720 |
|  |  |  | ALHF_04819.g3257 | 1060 | 4282 | 4955 |
|  |  |  | ALHF_04826.g3263 | 496 | 1633 | 1607 |
|  |  |  | ALHF_04832.g3269 | 416 | 1509 | 1489 |
|  |  |  | ALHF_04860.g3294 | 1057 | 1240 | 1141 |
|  |  |  | ALHF_04862.g3296 | 203 | 2397 | 3434 |
|  |  |  | ALHF_04865.g3297 | 773 | 2868 | 3743 |
|  |  |  | ALHF_04872.g3303 | 1303 | 5189 | 5185 |
|  |  |  | ALHF_04905.g3333 | 248 | 1030 | 1050 |
|  |  |  | ALHF_04954.g3369 | 112 | 416 | 835 |
|  |  |  | ALHF_05023.g3419 | 754 | 2746 | 4224 |
|  |  |  | ALHF_05032.g3425 | 1497 | 6374 | 7848 |
|  |  |  | ALHF_05297.g3632 | 471 | 8622 | 6783 |
|  |  |  | ALHF_05598.g3831 | 382 | 1432 | 2265 |
|  |  |  | ALHF_05721.g3904 | 766 | 4541 | 7178 |
|  |  |  | ALHF_05847.g3970 | 133 | 1736 | 430 |
|  |  |  | ALHF_05889.g3990 | 118 | 819 | 2235 |
|  |  |  | ALHF_05963.g4036 | 241 | 1002 | 2315 |
|  |  |  | ALHF_05982.g4044 | 610 | 3040 | 5397 |
|  |  |  | ALHF_06184.g4136 | 688 | 3829 | 4977 |
|  |  |  | ALHF_06191.g4139 | 265 | 1159 | 2014 |
|  |  |  | ALHF_06274.g4182 | 382 | 1302 | 1191 |
|  |  |  | ALHF_06344.g4223 | 223 | 854 | 1201 |
|  |  |  | ALHF_06366.g4234 | 112 | 700 | 927 |
|  |  |  | ALHF_06405.g4253 | 247 | 907 | 1161 |
|  |  |  | ALHF_06461.g4281 | 0 | 44 | 53 |
|  |  |  | ALHF_06470.g4285 | 146 | 523 | 1103 |
|  |  |  | ALHF_06554.g4331 | 111 | 472 | 730 |
|  |  |  | ALHF_06606.g4356 | 286 | 299 | 322 |
|  |  |  | ALHF_06661.g4387 | 326 | 1433 | 1636 |
|  |  |  | ALHF_06665.g4389 | 518 | 3051 | 4880 |
|  |  |  | ALHF_06680.g4398 | 184 | 638 | 1242 |
|  |  |  | ALHF_06704.g4408 | 270 | 1081 | 1793 |
|  |  |  | ALHF_06706.g4409 | 275 | 916 | 1703 |
|  |  |  | ALHF_06750.g4438 | 119 | 489 | 1081 |
|  |  |  | ALHF_06775.g4448 | 271 | 990 | 1851 |
|  |  |  | ALHF_06785.g4452 | 19 | 110 | 198 |
|  |  |  | ALHF_06797.g4459 | 212 | 1342 | 1790 |
|  |  |  | ALHF_06819.g4473 | 155 | 524 | 1067 |
|  |  |  | ALHF_06822.g4474 | 95 | 350 | 411 |
|  |  |  | ALHF_06841.g4488 | 272 | 1051 | 1266 |
|  |  |  | ALHF_06863.g4500 | 220 | 910 | 1397 |
|  |  |  | ALHF_06867.g4502 | 262 | 907 | 1319 |
|  |  |  | ALHF_06870.g4503 | 157 | 537 | 916 |
|  |  |  | ALHF_06879.g4509 | 315 | 1903 | 2872 |
|  |  |  | ALHF_06888.g4512 | 215 | 717 | 778 |
|  |  |  | ALHF_06906.g4519 | 306 | 1503 | 3490 |
|  |  |  | ALHF_06910.g4521 | 108 | 419 | 507 |
|  |  |  | ALHF_06918.g4524 | 257 | 1032 | 1634 |
|  |  |  | ALHF_06933.g4535 | 117 | 486 | 656 |
|  |  |  | ALHF_06941.g4538 | 100 | 1248 | 3204 |
|  |  |  | ALHF_06948.g4541 | 421 | 1561 | 1651 |
|  |  |  | ALHF_06951.g4542 | 116 | 753 | 891 |
|  |  |  | ALHF_06965.g4550 | 121 | 468 | 671 |
|  |  |  | ALHF_06981.g4559 | 254 | 3894 | 4997 |
|  |  |  | ALHF_06987.g4561 | 56 | 293 | 358 |
|  |  |  | ALHF_06992.g4564 | 133 | 820 | 1310 |
|  |  |  | ALHF_07001.g4568 | 101 | 452 | 635 |
|  |  |  | ALHF_07007.g4572 | 168 | 1390 | 2204 |
|  |  |  | ALHF_07056.g4603 | 212 | 899 | 1669 |
|  |  |  | ALHF_07064.g4609 | 72 | 491 | 773 |
|  |  |  | ALHF_07066.g4610 | 532 | 2658 | 4841 |
|  |  |  | ALHF_07073.g4616 | 285 | 1099 | 1742 |
|  |  |  | ALHF_07077.g4618 | 227 | 3088 | 6214 |
|  |  |  | ALHF_07100.g4632 | 159 | 1389 | 2291 |
|  |  |  | ALHF_07120.g4641 | 517 | 2709 | 7165 |
|  |  |  | ALHF_07153.g4654 | 262 | 1162 | 1892 |
|  |  |  | ALHF_07158.g4657 | 292 | 1415 | 2810 |
|  |  |  | ALHF_07162.g4658 | 80 | 460 | 692 |
|  |  |  | ALHF_07250.g4701 | 258 | 947 | 1831 |
|  |  |  | ALHF_07265.g4707 | 97 | 345 | 476 |
|  |  |  | ALHF_07273.g4712 | 83 | 667 | 1259 |
|  |  |  | ALHF_07290.g4721 | 387 | 3111 | 4587 |
|  |  |  | ALHF_07302.g4727 | 225 | 849 | 790 |
|  |  |  | ALHF_07318.g4733 | 262 | 954 | 1635 |
|  |  |  | ALHF_07336.g4740 | 152 | 569 | 920 |
|  |  |  | ALHF_07340.g4742 | 300 | 1088 | 2337 |
|  |  |  | ALHF_07345.g4745 | 131 | 459 | 621 |
|  |  |  | ALHF_07350.g4748 | 331 | 1631 | 1532 |
|  |  |  | ALHF_07366.g4756 | 312 | 1951 | 5198 |
|  |  |  | ALHF_07399.g4779 | 73 | 287 | 461 |
|  |  |  | ALHF_07407.g4782 | 148 | 1175 | 1971 |
|  |  |  | ALHF_07409.g4784 | 94 | 429 | 694 |
|  |  |  | ALHF_07430.g4794 | 462 | 2293 | 3725 |
|  |  |  | ALHF_07467.g4820 | 287 | 1906 | 3526 |
|  |  |  | ALHF_07478.g4825 | 70 | 341 | 294 |
|  |  |  | ALHF_07525.g4842 | 156 | 921 | 1194 |
|  |  |  | ALHF_07533.g4846 | 82 | 586 | 1193 |
|  |  |  | ALHF_07541.g4849 | 185 | 2210 | 3173 |
|  |  |  | ALHF_07545.g4853 | 313 | 1212 | 2621 |
|  |  |  | ALHF_07546.g4854 | 137 | 960 | 3535 |
|  |  |  | ALHF_07551.g4856 | 46 | 342 | 502 |
|  |  |  | ALHF_07561.g4861 | 89 | 725 | 1029 |
|  |  |  | ALHF_07586.g4873 | 233 | 857 | 1360 |
|  |  |  | ALHF_07617.g4888 | 184 | 666 | 1022 |
|  |  |  | ALHF_07642.g4896 | 143 | 1126 | 1922 |
|  |  |  | ALHF_07663.g4907 | 269 | 1720 | 3036 |
|  |  |  | ALHF_07677.g4914 | 264 | 1104 | 1332 |
|  |  |  | ALHF_07702.g4926 | 133 | 552 | 982 |
|  |  |  | ALHF_07703.g4927 | 100 | 904 | 2742 |
|  |  |  | ALHF_07711.g4929 | 82 | 436 | 867 |
|  |  |  | ALHF_07762.g4957 | 205 | 1224 | 2110 |
|  |  |  | ALHF_07786.g4971 | 116 | 481 | 762 |
|  |  |  | ALHF_07787.g4972 | 111 | 415 | 734 |
|  |  |  | ALHF_07857.g5007 | 107 | 638 | 404 |
|  |  |  | ALHF_07872.g5012 | 237 | 915 | 1114 |
|  |  |  | ALHF_07886.g5017 | 257 | 935 | 2502 |
|  |  |  | ALHF_07941.g5045 | 144 | 814 | 1373 |
|  |  |  | ALHF_07961.g5056 | 112 | 1435 | 2110 |
|  |  |  | ALHF_07966.g5060 | 141 | 727 | 1447 |
|  |  |  | ALHF_07972.g5063 | 97 | 336 | 584 |
|  |  |  | ALHF_07981.g5070 | 40 | 236 | 455 |
|  |  |  | ALHF_07994.g5078 | 134 | 633 | 1030 |
|  |  |  | ALHF_08042.g5100 | 113 | 392 | 632 |
|  |  |  | ALHF_08066.g5114 | 195 | 1811 | 3119 |
|  |  |  | ALHF_08076.g5121 | 132 | 675 | 1111 |
|  |  |  | ALHF_08130.g5141 | 148 | 572 | 1552 |
|  |  |  | ALHF_08165.g5159 | 76 | 727 | 996 |
|  |  |  | ALHF_08205.g5174 | 108 | 407 | 800 |
|  |  |  | ALHF_08237.g5190 | 192 | 752 | 1046 |
|  |  |  | ALHF_08263.g5200 | 124 | 880 | 1027 |
|  |  |  | ALHF_08270.g5205 | 107 | 365 | 876 |
|  |  |  | ALHF_08282.g5211 | 307 | 1774 | 3419 |
|  |  |  | ALHF_08290.g5213 | 66 | 319 | 619 |
|  |  |  | ALHF_08295.g5215 | 48 | 194 | 242 |
|  |  |  | ALHF_08322.g5231 | 140 | 707 | 1153 |
|  |  |  | ALHF_08344.g5241 | 81 | 475 | 1173 |
|  |  |  | ALHF_08381.g5255 | 235 | 1306 | 2940 |
|  |  |  | ALHF_08398.g5263 | 103 | 362 | 774 |
|  |  |  | ALHF_08417.g5271 | 312 | 1637 | 1859 |
|  |  |  | ALHF_08433.g5280 | 181 | 1232 | 818 |
|  |  |  | ALHF_08498.g5308 | 69 | 408 | 664 |
|  |  |  | ALHF_08530.g5324 | 246 | 1116 | 1361 |
|  |  |  | ALHF_08553.g5334 | 77 | 284 | 400 |
|  |  |  | ALHF_08564.g5338 | 77 | 353 | 773 |
|  |  |  | ALHF_08590.g5350 | 166 | 652 | 1671 |
|  |  |  | ALHF_08592.g5351 | 733 | 4893 | 6598 |
|  |  |  | ALHF_08602.g5356 | 119 | 981 | 1802 |
|  |  |  | ALHF_08606.g5358 | 59 | 236 | 201 |
|  |  |  | ALHF_08618.g5363 | 21 | 88 | 129 |
|  |  |  | ALHF_08635.g5370 | 281 | 922 | 1141 |
|  |  |  | ALHF_08715.g5403 | 69 | 429 | 677 |
|  |  |  | ALHF_08737.g5408 | 49 | 279 | 440 |
|  |  |  | ALHF_08797.g5430 | 98 | 102 | 326 |
|  |  |  | ALHF_08801.g5432 | 77 | 344 | 300 |
|  |  |  | ALHF_08809.g5434 | 262 | 1336 | 2672 |
|  |  |  | ALHF_08822.g5438 | 200 | 4489 | 9568 |
|  |  |  | ALHF_08863.g5451 | 52 | 3141 | 7872 |
|  |  |  | ALHF_08923.g5473 | 220 | 855 | 1504 |
|  |  |  | ALHF_08942.g5482 | 49 | 203 | 167 |
|  |  |  | ALHF_08944.g5483 | 18 | 1154 | 2026 |
|  |  |  | ALHF_09073.g5519 | 105 | 468 | 686 |
|  |  |  | ALHF_09075.g5520 | 184 | 647 | 1434 |
|  |  |  | ALHF_09080.g5522 | 122 | 688 | 2393 |
|  |  |  | ALHF_09152.g5554 | 254 | 1047 | 1824 |
|  |  |  | ALHF_09220.g5579 | 61 | 431 | 862 |
|  |  |  | ALHF_09232.g5584 | 81 | 349 | 857 |
|  |  |  | ALHF_09239.g5586 | 259 | 1046 | 1349 |
|  |  |  | ALHF_09257.g5590 | 127 | 741 | 1715 |
|  |  |  | ALHF_09265.g5594 | 62 | 263 | 593 |
|  |  |  | ALHF_09309.g5608 | 201 | 834 | 1659 |
|  |  |  | ALHF_09573.g5689 | 39 | 190 | 556 |
|  |  |  | ALHF_09653.g5699 | 30 | 167 | 800 |
|  |  |  | ALHF_09670.g5703 | 65 | 268 | 414 |
|  |  |  | ALHF_09924.g5731 | 715 | 3058 | 6966 |
|  |  |  | ALHF_10035.g5773 | 643 | 5795 | 2157 |
|  |  |  | ALHF_10036.g5774 | 162 | 4087 | 6546 |
|  |  |  | ALHF_10046.g5779 | 21 | 160 | 223 |
|  |  |  | ALHF_10108.g5795 | 58 | 378 | 507 |
|  |  |  | ALHF_10133.g5798 | 110 | 581 | 870 |
|  |  |  | ALHF_10134.g5799 | 174 | 903 | 1849 |
|  |  |  | ALHF_10154.g5805 | 68 | 451 | 582 |
|  |  |  | ALHF_10189.g5819 | 53 | 201 | 487 |
|  |  |  | ALHF_10198.g5822 | 614 | 2113 | 2011 |
|  |  |  | ALHF_10267.g5842 | 52 | 579 | 210 |
|  |  |  | ALHF_10383.g5873 | 1140 | 3997 | 3902 |
|  |  |  | ALHF_10425.g5882 | 300 | 2193 | 3703 |
|  |  |  | ALHF_10443.g5887 | 162 | 4087 | 6546 |
|  |  |  | ALHF_10506.g5910 | 62 | 463 | 549 |
|  |  |  | ALHF_10508.g5912 | 199 | 1099 | 660 |
|  |  |  | ALHF_10549.g5929 | 189 | 760 | 774 |
|  |  |  | ALHF_10550.g5930 | 49 | 680 | 197 |
|  |  |  | ALHF_10571.g5933 | 105 | 883 | 392 |
|  |  |  | ALHF_10597.g5942 | 206 | 804 | 996 |
|  |  |  | ALHF_10868.g6050 | 405 | 2575 | 2775 |
|  |  |  | ALHF_10972.g6094 | 259 | 1861 | 1219 |
|  |  |  | ALHF_11004.g6116 | 1016 | 4375 | 4236 |
|  |  |  | ALHF_11025.g6126 | 115 | 431 | 774 |
|  |  |  | ALHF_11031.g6127 | 927 | 3873 | 5322 |
|  |  |  | ALHF_11170.g6213 | 82 | 415 | 338 |
|  |  |  | ALHF_11187.g6221 | 1062 | 3660 | 5478 |
|  |  |  | ALHF_11234.g6247 | 391 | 13791 | 1397 |
|  |  |  | ALHF_11262.g6259 | 321 | 1136 | 1939 |
|  |  |  | ALHF_11279.g6271 | 213 | 795 | 1115 |
|  |  |  | ALHF_11327.g6305 | 667 | 2591 | 4430 |
|  |  |  | ALHF_11336.g6315 | 14 | 235 | 169 |
|  |  |  | ALHF_11337.g6316 | 14 | 235 | 169 |
|  |  |  | ALHF_11385.g6352 | 96 | 339 | 526 |
|  |  |  | ALHF_11399.g6357 | 118 | 410 | 633 |
|  |  |  | ALHF_11400.g6358 | 118 | 410 | 633 |
|  |  |  | ALHF_11536.g6458 | 108 | 393 | 668 |
|  |  |  | ALHF_11568.g6476 | 213 | 1118 | 627 |
|  |  |  | ALHF_11574.g6481 | 62 | 577 | 485 |
|  |  |  | ALHF_11580.g6487 | 96 | 339 | 526 |
|  |  |  | ALHF_11628.g6518 | 522 | 2459 | 5222 |
|  |  |  | ALHF_11637.g6527 | 694 | 3846 | 4869 |
|  |  |  | ALHF_11641.g6529 | 44 | 235 | 302 |
|  |  |  | ALHF_11697.g6560 | 129 | 955 | 585 |
|  |  |  | ALHF_11753.g6602 | 410 | 2603 | 2198 |
|  |  |  | ALHF_11774.g6617 | 104 | 404 | 433 |
|  |  |  | ALHF_11775.g6618 | 104 | 404 | 433 |
|  |  |  | ALHF_11789.g6624 | 1057 | 1240 | 1141 |
|  |  |  | ALHF_11804.g6633 | 112 | 416 | 835 |
|  |  |  | ALHF_11888.g6687 | 643 | 5795 | 2157 |
|  |  |  | ALHF_11921.g6697 | 21 | 160 | 223 |
|  |  |  | ALHF_11978.g6721 | 284 | 3082 | 4218 |
|  |  |  | ALHF_12015.g6727 | 0 | 78 | 50 |
|  |  |  | ALHF_12130.g6746 | 81 | 403 | 996 |
|  |  |  | ALHF_12287.g6759 | 33 | 160 | 153 |
|  |  |  | ALHF_12606.g6774 | 40 | 156 | 385 |
|  |  |  | ALHF_12900.g6788 | 24 | 111 | 136 |
|  |  |  | ALHF_12943.g6789 | 17 | 171 | 186 |
|  |  |  | ALHF_12974.g6793 | 18 | 81 | 102 |
|  |  |  | ALHF_13335.g6807 | 12 | 135 | 312 |
|  |  |  | ALHF_14262.g6837 | 34 | 350 | 566 |
|  |  |  | ALHF_00005.g4 | 80 | 1421 | 2216 |
|  |  |  | ALHF_00027.g20 | 141 | 477 | 732 |
|  |  |  | ALHF_00130.g69 | 200 | 935 | 1432 |
|  |  |  | ALHF_00386.g202 | 31 | 308 | 374 |
|  |  |  | ALHF_00402.g211 | 161 | 648 | 1077 |
|  |  |  | ALHF_00814.g445 | 93 | 483 | 908 |
|  |  |  | ALHF_00912.g506 | 281 | 936 | 1333 |
|  |  |  | ALHF_00939.g521 | 140 | 547 | 572 |
|  |  |  | ALHF_01012.g558 | 393 | 1925 | 3075 |
|  |  |  | ALHF_01216.g667 | 58 | 554 | 908 |
|  |  |  | ALHF_01241.g678 | 461 | 1585 | 1428 |
|  |  |  | ALHF_01411.g773 | 26 | 163 | 277 |
|  |  |  | ALHF_01582.g874 | 82 | 470 | 898 |
|  |  |  | ALHF_01650.g922 | 195 | 1340 | 2560 |
|  |  |  | ALHF_01675.g939 | 205 | 945 | 1027 |
|  |  |  | ALHF_01702.g955 | 476 | 1640 | 2597 |
|  |  |  | ALHF_01703.g956 | 39 | 346 | 864 |
|  |  |  | ALHF_02003.g1133 | 180 | 775 | 1599 |
|  |  |  | ALHF_02009.g1138 | 115 | 452 | 869 |
|  |  |  | ALHF_02303.g1337 | 180 | 861 | 1147 |
|  |  |  | ALHF_02693.g1572 | 211 | 891 | 1348 |
|  |  |  | ALHF_02843.g1694 | 144 | 1057 | 1122 |
|  |  |  | ALHF_02900.g1734 | 192 | 799 | 1321 |
|  |  |  | ALHF_02916.g1744 | 436 | 1715 | 3002 |
|  |  |  | ALHF_02938.g1760 | 66 | 290 | 389 |
|  |  |  | ALHF_03049.g1849 | 80 | 314 | 526 |
|  |  |  | ALHF_03296.g2026 | 336 | 1828 | 1761 |
|  |  |  | ALHF_03581.g2233 | 54 | 457 | 445 |
|  |  |  | ALHF_03982.g2555 | 115 | 468 | 749 |
|  |  |  | ALHF_04314.g2836 | 1555 | 5699 | 7433 |
|  |  |  | ALHF_04414.g2911 | 533 | 1816 | 2131 |
|  |  |  | ALHF_04546.g3029 | 214 | 741 | 628 |
|  |  |  | ALHF_05021.g3417 | 593 | 1955 | 2034 |
|  |  |  | ALHF_05215.g3570 | 5338 | 6782 | 5821 |
|  |  |  | ALHF_06132.g4115 | 232 | 820 | 787 |
|  |  |  | ALHF_06239.g4167 | 219 | 1099 | 1452 |
|  |  |  | ALHF_06332.g4218 | 95 | 385 | 707 |
|  |  |  | ALHF_06747.g4436 | 192 | 781 | 770 |
|  |  |  | ALHF_07029.g4586 | 257 | 1071 | 1775 |
|  |  |  | ALHF_07196.g4677 | 106 | 360 | 657 |
|  |  |  | ALHF_07335.g4739 | 28 | 191 | 329 |
|  |  |  | ALHF_07372.g4761 | 175 | 819 | 1317 |
|  |  |  | ALHF_07603.g4884 | 71 | 696 | 212 |
|  |  |  | ALHF_07621.g4889 | 133 | 620 | 1051 |
|  |  |  | ALHF_07641.g4895 | 36 | 160 | 212 |
|  |  |  | ALHF_07761.g4956 | 32 | 179 | 212 |
|  |  |  | ALHF_07778.g4965 | 130 | 1463 | 3044 |
|  |  |  | ALHF_07858.g5008 | 71 | 306 | 492 |
|  |  |  | ALHF_08193.g5171 | 115 | 418 | 697 |
|  |  |  | ALHF_08238.g5191 | 85 | 335 | 455 |
|  |  |  | ALHF_08391.g5260 | 171 | 695 | 1012 |
|  |  |  | ALHF_08397.g5262 | 95 | 348 | 506 |
|  |  |  | ALHF_08571.g5342 | 63 | 288 | 404 |
|  |  |  | ALHF_08657.g5379 | 176 | 747 | 1492 |
|  |  |  | ALHF_08743.g5409 | 41 | 190 | 450 |
|  |  |  | ALHF_08837.g5443 | 62 | 291 | 449 |
|  |  |  | ALHF_08941.g5481 | 69 | 326 | 751 |
|  |  |  | ALHF_08953.g5486 | 82 | 706 | 1737 |
|  |  |  | ALHF_08958.g5489 | 150 | 515 | 1004 |
|  |  |  | ALHF_09114.g5536 | 85 | 1428 | 3440 |
|  |  |  | ALHF_09380.g5629 | 26 | 183 | 345 |
|  |  |  | ALHF_09537.g5678 | 164 | 787 | 1145 |
|  |  |  | ALHF_09715.g5705 | 15 | 81 | 116 |
|  |  |  | ALHF_10769.g6003 | 126 | 445 | 484 |
|  |  |  | ALHF_10773.g6005 | 8 | 78 | 52 |
|  |  |  | ALHF_10846.g6037 | 841 | 2912 | 4801 |
|  |  |  | ALHF_10870.g6051 | 58 | 321 | 596 |
|  |  |  | ALHF_10873.g6052 | 49 | 183 | 430 |
|  |  |  | ALHF_10891.g6062 | 962 | 1194 | 1100 |
|  |  |  | ALHF_10915.g6070 | 115 | 468 | 654 |
|  |  |  | ALHF_11330.g6309 | 130 | 693 | 1613 |
|  |  |  | ALHF_11713.g6574 | 257 | 1444 | 1800 |
|  |  |  | ALHF_12972.g6792 | 19 | 185 | 314 |
|  |  |  | ALHF_13122.g6798 | 17 | 110 | 163 |
|  |  |  | ALHF_13444.g6812 | 47 | 199 | 270 |
|  |  |  | ALHF_13764.g6822 | 25 | 275 | 558 |
|  |  |  | ALHF_13836.g6826 | 26 | 193 | 488 |

^†^Structural Classification of Proteins (SCOP) database for the *D. melanogaster* proteome (v1.73). http://supfam.cs.bris.ac.uk/SUPERFAMILY/

^§^Gene predicted function as evidenced by the Pfam HMM-A scanning (http://pfam.sanger.ac.uk/)

^††^m/tr = metabolism/transport

*Fragments Per Kilobases of gene length per Million RNASeq reads mapped

**Tested for relative gene expression in the different *M. domestica* autosomal combination lines in Table 4
